# From discovery to delivery: public sector development of the *r*VSV-ZEBOV Ebola vaccine

**DOI:** 10.1093/jlb/lsz019

**Published:** 2020-01-16

**Authors:** Matthew Herder, Janice E Graham, Richard Gold

**Affiliations:** 1 Health Law Institute, Schulich School of Law, Dalhousie University; 2 Department of Pharmacology, Faculty of Medicine, Dalhousie University; 3 Department of Pediatrics (Infectious Diseases), Faculty of Medicine, Dalhousie University; 4 Faculty of Law and Faculty of Medicine, McGill University

**Keywords:** Ebola vaccine, patents, commercialization, open science

## Abstract

The discovery and development of the Ebola *r*VSV-ZEBOV vaccine challenge the common assumption that the research and development for innovative therapeutic products and vaccines is best carried out by the private sector. Using internal government documents obtained through an access to information request, we analyze the development of *r*VSV-ZEBOV by researchers at Canada’s National Microbiology Laboratory beyond its patenting and licensing to a biotech company in the United States in 2010. According to government documentation, the company failed to make any progress toward a phase 1 clinical trial until after the WHO Public Health Emergency of International Concern freed substantial donor and public funds for the vaccine’s further development. The development of *r*VSV-ZEBOV, from sponsoring early stage research through to carrying out clinical trials during the epidemic, was instead the result of the combined efforts of the Canadian government, its researchers, and other publicly funded institutions. This case study of *r*VSV-ZEBOV underscores the significant public contribution to the R&D of vaccines even under conditions of precarity, and suggests that an alternative approach to generating knowledge and developing interventions, such as open science, is required in order to fully realize the public sector’s contribution to improved global health.

It is an open secret that the public sector has long contributed substantially—in terms of scientific labor, know-how, and direct financing—to the development of many important drugs and vaccines^1^. These contributions often only come to light after the fact, once a publicly developed drug is priced exorbitantly under the control of a private company^2^. Acting on the theory that only the private sector can successfully commercialize a drug or vaccine, government laboratories and universities risk achieving the opposite: delays, increased prices, and decreased access.

The academic literature contains a substantial critique of this standard approach to commercialization,^3^ but concrete examples are needed to motivate policy change. In this analysis, we provide an in-depth examination of the development of a promising experimental Ebola vaccine, *r*VSV-ZEBOV, showing how the private sector was not only unnecessary to its development, but also likely slowed it down. Using over 1600 pages of internal government records obtained through an access to information request,^4^ we trace the development of *r*VSV-ZEBOV between the early 2000s to its licensing to a small US-based biotech company in 2010^5^ through to the 2014–2015 Ebola epidemic in West Africa (see Figure 1) and beyond. We reveal how Canadian government scientists drove the development of *r*VSV-ZEBOV, from laboratory bench to a commercial grade product for use in clinical trials, while private sector partners failed to substantively advance development in the years leading up to the West African epidemic.

**Figure 1:**
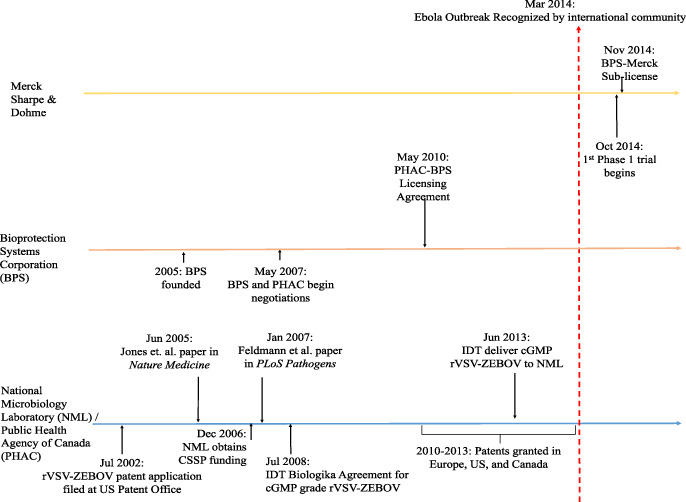
Key milestones and events during the development of *r*VSV-ZEBOV in the lead up to the 2014–2015 Ebola epidemic.

The analysis shows how the standard approach to commercialization also works to reproduce the status quo. Even as it advanced the *r*VSV-ZEBOV vaccine, reductions in human and financial resources undercut the government laboratory’s capacity to investigate alternative pathways to development, assess the track record of private sector partners, and enforce certain terms of the patent licensing agreement. Despite resolving scientific, technical, and logistical challenges during the research and development process as well as acquiring expertise about manufacturing clinical grade quality vaccine, the government laboratory transferred the knowledge and vaccine supply that it had amassed to a small company that had never brought a product to market.

We conclude that not only does the dominant account of the private sector’s importance to commercialization ignore the facts behind the *r*VSV-ZEBOV vaccine development, but that sole reliance on the private sector sets a concrete ceiling on how far public sector science can reach, pre-empting experimentation with new approaches to knowledge production. Rather than explore alternative pathways to developing the vaccine, the public sector largely handed over responsibility for development to a private sector unequal to the task. More than 5 years after *r*VSV-ZEBOV was first deployed in the West African epidemic and trialed in thousands of Ebola patients, the private sector firm controlling the patent finally obtained approval to market the vaccine from European and United States regulators.^6^ Instead of celebrating this milestone, our analysis raises the question as to whether *r*VSV-ZEBOV could have been available earlier if public laboratories had taken a different approach to the vaccine’s development.

## Public Financing, Discovery, and Development of VSV-ZEBOV

As with other neglected diseases, public and philanthropic sectors have been at the forefront of funding Ebola research. Public sources contributed over 73% (USD$758.8 million) of the USD$1.035 billion allocated to Ebola and other filovirus research from 1997 to 2015^7^. Much of this public funding followed from political concerns over bioterrorism in the early 2000s^8^. For its part, Canada created a special stream of biosecurity funding led by Defence Research and Development Canada, later known as the Canadian Safety and Security Program (CSSP)^9^.

The beginning of the discovery work on the *r*VSV-ZEBOV vaccine traces back to 1999 when the National Microbiology Laboratory (NML) in Winnipeg, Manitoba recruited Heinz Feldmann from the University of Marburg to become chief of its special pathogens program. With an exceptional background in hemorrhagic fever virus of the Filoviridae family, within three years Feldmann and his colleagues developed an Ebola vaccine candidate: *r*VSV-ZEBOV^10^. NML later became part of the Public Health Agency of Canada (PHAC).

As required under Canadian law applicable to government scientists^11^, Feldmann and colleagues Steven Jones and Ute Stroher disclosed the invention to the NML. The Canadian government filed a provisional US patent application in July 2002 entitled “Recombinant Vesicular Stomatitis Virus Vaccines for Viral Hemorrhagic Fevers”^12^. Patent offices in Europe, the United States, and Canada eventually granted the *r*VSV-ZEBOV patent in 2010, 2011, and 2013, respectively.

The theory behind the standard approach to commercialization holds that patents will facilitate follow-on research and development^13^. Despite the patent application and a series of publications by Feldmann’s team indicating that the *r*VSV-ZEBOV vaccine was highly effective in mice and nonhuman primates^14^, there was initially no commercial interest in the vaccine. In 2005, PHAC entered into a material transfer agreement with the US Army Medical Research Institute of Infectious Disease (USAMRIID); however, the agreement was for non-commercial research only.

**Figure 2:**
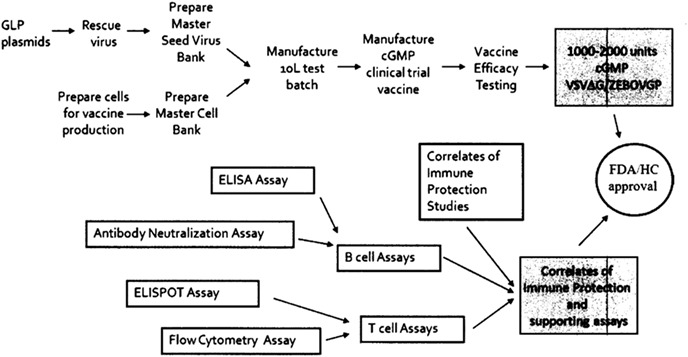
The two main branches of the NML’s *r*VSV-ZEBOV project. *Notes:* (1) This figure was included in the NML’s Project Completion Report, dated March 2014.

To develop the vaccine, NML scientists instead secured funding under the government’s CSSP program in 2006 in pursuit of two objectives. The first was to manufacture 1000–2000 doses of “cGMP grade” vaccine for use in clinical trials. Second, the scientists sought to understand the immune correlates of protection for an Ebola virus infection and develop treatment protocols and assays in order to obtain regulatory approval for *r*VSV-ZEBOV from Health Canada (HC) and the US Food and Drug Administration (FDA) (see Figure 2). A total of Cdn$5.9 million (including in-kind contributions from NML as well as USAMRIID) was budgeted for the project during 2007–2013, which was later extended to March 2014.^15^

Within months of securing the CSSP funding, NML began discussions over a possible patent license agreement with BioProtection Systems Inc. (BPS), a small start-up located in Iowa State University’s research park. While the then Canadian Minister of Health stated in Parliament that there had been an open and transparent call for proposals, there is no evidence of this in the record nor any suggestion that any other company submitted or was asked to submit a proposal.

In December 2007, BPS submitted a development plan to PHAC, claiming that it and its “developmental partners have the capability and capacity to manufacture, to bring drugs to the clinic and to follow the development of a drug through to [regulatory] approval”.^16^ BPS may have been relying on the capacity of its partner, NewLink Genetics, another Iowa State University spin-off company; however, neither BPS nor NewLink, which acquired BPS in 2011, has ever developed a product through to regulatory approval. Nevertheless, BPS proposed a work plan in which it would carry out additional preclinical vaccine testing, perform safety and toxicology studies, assist with the manufacturing of the vaccine, and conduct clinical trials with *r*VSV-ZEBOV.^17^

PHAC forwarded patent license terms to BPS in 2008.^18^ While normal practice for a request for proposals would have required that the terms of the license be agreed to as part of BPS’s proposal, PHAC continued to negotiate the terms of the license over the next two years. During this time, the NML continued its work on the vaccine. In 2008, the NML oversaw the production of plasmids for the reverse genetics system used to generate *r*VSV-ZEBOV^19^ and also arranged for the production of cGMP grade *r*VSV-ZEBOV through a contract with IDT Biologika, a German contract research organization specializing in vaccine manufacturing.

PHAC and BPS formally concluded a license on May 4, 2010. ^20^ The agreement provided BPS with a “sole” license to the vaccine but with specific carve-outs protecting PHAC’s ability to use the patent rights for non-commercial, educational, and research purposes and rights to use the invention in the context of a humanitarian emergency in Canada or internationally^21^. BPS was given the power to sub-license the vaccine without prior approval from the Canadian government, but PHAC’s protections would run with any sub-licensing deal.

In conformity with good licensing practices, BPS agreed to make “reasonable efforts” to commercialize the vaccine and deliver “written reports” on its progress. Further, the license clearly provided that shelving the vaccine would be tantamount to a “fundamental breach” of the contract. While the publicly available version of the license blacked out financial terms, NewLink’s regulatory filings show that BPS had agreed to pay for all patent-related fees and costs, pay milestone payments of approximately Cdn$205,000 per regulatory-approved product, and a royalty as a low single-digit percentage of sales to Canada^22^. Consistent with standard practices of today,^23^ the agreement was silent with respect to the pricing of any resulting product.

After signing the agreement, PHAC claimed that BPS “had the corporate capabilities to develop the *r*VSV-ZEBOV vaccine into a fully regulatory-approved product”^24^ even though the company had no experience of this stage of vaccine development.

## Transferring VSV-ZEBOV to BPS

During the same period as the negotiations, the NML’s internal capacity to drive *r*VSV-ZEBOV’s development was diminishing. The scientists that had led the basic and preclinical research on *r*VSV-ZEBOV, notably Feldmann, Jones, and Stroher, had departed the NML by late 2010. Remaining high profile researchers prioritized other Ebola therapeutic leads^25^ (24). Further, scientists in the civil service were, under the then government, subject to increasing constraints and resource uncertainties^26^. Midway through the NML’s *r*VSV-ZEBOV project, for instance, the flexibility to carry over unused CSSP funds to the next fiscal year disappeared.^27^ With financial and human resources in flux, the NML contemplated canceling the *r*VSV-ZEBOV project altogether. Judie Alimonti, employed by the NML on a limited term contract, volunteered to take over as project manager.

During 2010–2014, Alimonti worked through myriad challenges and delays with the NML’s partners. A poorly drafted contract with IDT resulted in a “constant stream of negotiations” over who was responsible for each deliverable.^28^ Early on, IDT struggled to create the Master Seed Virus Bank, an important preliminary step in the manufacturing process.^29^ Later, Alimonti expressed growing frustrations with various delays on IDT’s end, especially as the CSSP funding was set to end in March 2013.^30^ After two failed efforts to generate the “clinical trial material,” IDT finally delivered 1350 doses of the vaccine to the NML in June 2013.^31^ Canada paid IDT Biologika Cdn$877,422.15 for the vaccine’s production^32^.

BPS’ contributions during the 4 years leading up to the West African epidemic are less clear. Contrary to the terms of license, BPS failed to deliver a single written report of its commercialization progress during 2010–2014. Other documentation betrays a pattern of delay. Originally, BPS had planned to use a 10 L test batch of the vaccine (produced by IDT) for further pre-clinical testing.^33^ However, BPS elected to wait for PHAC’s order of IDT cGMP grade vaccine for such testing, ostensibly to be more in line with the FDA’s guidance for Phase 1 trials involving experimental vaccines^34^.

BPS’ lack of alacrity in running pre-clinical experiments was matched by its lack of diligence in meeting with the FDA. In May 2011, IDT suggested that BPS ask the FDA what types of testing would be needed to support an “Investigational New Drug” (IND) application to conduct a Phase 1 trial. ^35^ It took, however, another year and half for BPS to arrange a “pre-IND” meeting with the FDA to clarify the regulator’s expectations. ^36^ In terms of experimental contributions, BPS also failed to advance the file. It had planned in 2011 to proceed with both the vaccine pre-exposure and post-exposure protocols for testing the efficacy of the cGMP grade *r*VSV-ZEBOV once it was in hand. Yet, by the time IDT had delivered the vaccine to NML in June 2013, BPS bowed out, leaving it to the NML to perform efficacy testing September–October 2013.^37^ In short, BPS did not complete any of the experimental objectives outlined in its vaccine development plan.

With the CSSP funding coming to an end, the NML prepared a final project report just as the government of Guinea reported tens of Ebola deaths in March 2014^38^. Despite BPS’ meager efforts, the NML’s report was optimistic, emphasizing that there was to be “life after the project.” The NML planned to transfer to BPS not only the immunological assays and accompanying treatment protocols, but also the cGMP vaccine that the NML had secured from IDT. BPS would then finally commence Phase 1 and 2 clinical trials, albeit in partnership with the US National Institutes of Health.

## Epidemics as Epilog

The escalation of the Ebola outbreak over the summer of 2014 interrupted the knowledge transfer from NML to BPS. Instead of sending all of the cGMP vaccine to BPS, the Canadian government donated 800 vials of *r*VSV-ZEBOV to the WHO for use in West Africa^39^. Questions began to be raised publicly about the delay in development of a vaccine that had been shown to be 100% effective in animal models as early as 2004^40^. In November 2014, BPS sublicensed the patent rights to *r*VSV-ZEBOV to Merck, Sharp & Dohme^41^. Meanwhile, using the IDT Biologika-sourced cGMP grade vaccine, the public sector paid for the first Phase 1 clinical trials, which began in the United States in October 2014 (NCT02269423) and in Geneva, Hamburg, and Kilifi in November 2014^42^. The Canadian Center for Vaccinology also began a Phase 1 trial, funded by the Canadian government, in November 2014 in Halifax (NCT02374385)^43^.

**Table 1:** Funding received by BioProtection Systems Inc. 2008–2016 in USD$.

Fiscal Year	Dept. of Health & Human Services	Dept. of Defense	Dept. of Agriculture	Total	Pre- & Post-Epidemic Total
2008	462,742	69,990	-	532,732	$9,688,115
2009	43,062	630,738	-	4,947,313	(2008–2013)
	535,681	29,995			
		3,707,837			
2010	299,975	119,261	-	419,236	
2011	299,920	3,388,914	100,000	3,788,834	
2012	-	-	-	-	
2013	-	-	-	-	
2014	-	1,000,000	-	2,885,133	$119,355,036
		1,885,871			(2014–2016)
		(738)			
2015	29,967,985	1,025,080	(10,000)	63,969,873	
	17,883,564	8,168,814			
	4,467,990	1,504,482			
		961,958			
2016	24,752,733	2,794,840	-	52,500,031	
	2,227,245	534,323			
	593,685	(15,458)			
	21,681,676	(69,013)			
**Total**	$103,216,258	$25,736,893	$90,000	$129,043,151	

*Notes:* (1) Financial figures in the table are derived from https://www.usaspending.gov/; (2) Figures that appear in parentheses () reflect a liability; (3) Multiple figures in a given fiscal year are indicative of separate grants or contracts; (4) One figure (USD$29,967,984 in fiscal year 2015 from the Dept. of Health & Human Services is actually dated Dec. 19, 2014; however, the website lists this contract under fiscal year 2015; and (5) Not all figures are related to Ebola research. For example, the figures listed under Dept. of Health & Human Services for fiscal years 2010 and 2011 are grants for research into yellow fever and adenoviruses. Other figures, especially prior to the 2014–2015 epidemic, may be unrelated to Ebola research as well but this information was not available.

BPS (now owned by NewLink) received USD$50 million from its sublicense to Merck^44^ as well as an injection of USD$119,355,036 in funding from US government sources (See Table 1). After assuming *de jure* control of the *r*VSV-ZEBOV, Merck earned recognition for its in-kind support of the clinical trials that were run during the 2014–2015 epidemic, most notably, the WHO-led consortium “*Ebola Ça Suffit!*” Phase 3 trial in Guinea involving 11,841 participants^45^. It was unclear what Merck did during this period other than provide permission to use the Canadian procured and financed *r*VSV-ZEBOV clinical grade vaccine. What the record does establish is that it was the public sector, not Merck, that provided all of the financing, including for clinical trials, during the West African epidemic (see Table 2)^46^ in addition to providing the technical expertise, human resources, and infrastructure that was necessary to carry out the trials.

**Table 2:** Clinical Trials of *r*VSV-ZEBOV Initiated During West Africa Ebola Epidemic.

Identifier	Sponsor (s)	Collaborator (s)	Phase	Start date	Completion date	#Participants
NCT02269423	**Merck Sharp & Dohme Corp.**	**BioProtection Systems Corporation**	1	Oct. 2014	Aug. 2015	39
		United States Department of Defense				
NCT02280408	**Merck Sharp & Dohme Corp.**	National Institute of Allergy and Infectious Diseases (NIAID)	1	Oct. 2014	Dec. 2015	39
		**BioProtection Systems Corporation**				
NCT02374385	Dalhousie University	Canadian Institutes of Health Research (CIHR)	1	Nov. 2014	Jun. 2015	40
		**NewLink Genetics Corporation**				
NCT02283099	Universitätsklinikum Hamburg- Eppendorf	German Center for Infection Research	1	Nov. 2014	Nov. 2015	30
		Philipps University Marburg Medical Center World Health Organization				
		Clinical Trial Center North University Hospital, Geneva Albert Schweitzer Hospital				
		Institute of Tropical Medicine, University of Tuebingen Wellcome Trust				
		KEMRI-Wellcome Trust Collaborative Research Program				
NCT02287480	University Hospital, Geneva	World Health Organization	1	Nov. 2014	Jan. 2016	115
		Wellcome Trust				
		Universitätsklinikum Hamburg-Eppendorf Philipps University Marburg Medical Center Albert Schweitzer Hospital				
		Institute of Tropical Medicine, University of Tuebingen KEMRI-Wellcome Trust Collaborative Research Program				
NCT02296983	University of Oxford	World Health Organization	1	Dec. 2014	Sep. 2016	40^*^
		Wellcome Trust				
		Institute of Tropical Medicine, University of Tuebingen Albert Schweitzer Hospital				
		Philipps University Marburg Medical Center Universitätsklinikum Hamburg-Eppendorf University Hospital, Geneva				
NCT02314923	**Merck Sharp & Dohme Corp.**	**BioProtection Systems Corporation**	1	Dec. 2014	Jun. 2016	512
		US Department of Health and Human Services				
NCT02344407 “PREVAIL”	National Institute of Allergy and Infectious Diseases (NIAID)	None	2	Jan. 20, 2015	Jun. 1, 2020^*^	1500^*^
PACTR201503001057193						
“Ebola ca suffit!”	World Health Organization	Ministry of Health Guinea	3	Mar. 3, 2015	Jan. 20, 2016	8851
		Medecins Sans Frontieres Epicentre				
		Norwegian Institute of Public Health				
		Institute of Social and Preventive Medicine and Centre for clinical trials University of Bern				
		London School of Hygiene and Tropical Medicine University of Florida				
		Centre for Vaccine Development, Mali/Maryland Public Health England				
		Public Health Agency of Canada				
		**NewLink/Merck**				
NCT02378753 “STRIVE”	Centers for Disease Control and	University of Sierra Leone	2/3	Apr. 2015	Dec. 5, 2016	8651
	Prevention	Ministry of Health and Sanitation, Sierra Leone				
		Department of Health and Human Services eHealth Africa				
NCT02503202	**Merck Sharp & Dohme Corp.**	None	3	Aug. 17, 2015	Sep. 29, 2017^*^	1198
NCT02933931	University Hospital, Geneva	None	1	Nov. 2016	Apr. 2020^*^	100^*^
NCT02876328	National Institute of Allergy and	The Liberia-US Clinical Trials Partnership Program, Partnership	2	Mar. 31, 2017	Mar. 2019^*^	5500^*^
	Infectious Diseases (NIAID)	for Research on Ebola Virus in Liberia Project (PREVAIL)				
		Institut National de la Santé Et de la Recherche Médicale, France				
		London School of Hygiene and Tropical Medicine				

*Notes:* (1) All data in this table are derived from https://clinicaltrials.gov; (2) Entries in bold indicate that a private sector entity was involved, either as a sponsor or collaborator, in a given clinical trial; (3) Despite being named a sponsor in four trials, the publications that have resulted from these trials do not state that Merck, Sharp & Dohme provided financing for those trials; (4) Figures with an asterisk (^*^) reflect estimated figures at the time of data extraction from clinicaltrials.gov; (5) Although the final trial in the table was initiated in March 2017, after the epidemic had subsided, it is included here because it builds upon a previous trial known as the “PREVAIL” trial (NCT02344407) that was initiated during the epidemic.

Through 2016–2017 multilateral (Gavi, the Vaccine Alliance USD$5 million^47^) and government (US Department of Health and Human Services, USD$39.2 million^48^) sources continued to fund Merck’s late stage development of the vaccine. The funding from Gavi reportedly came with an obligation to create stockpile doses of *r*VSV-ZEBOV and ensure the vaccine was priced affordably in developing countries,^49^ but the precise terms of the Gavi-Merck agreement are not publicly available.

Merck missed the 2017 target specified in the Gavi agreement for the vaccine’s submission to the FDA^50^. But by November 2018, when over 42,000 frontline health workers and Ebola contacts had (at that time) been vaccinated through a ring vaccination strategy in the outbreak that was continuing to unfold in the Democratic Republic of the Congo,^51^ and the data from 12 *r*VSV-ZEBOV trials now in hand, Merck finally initiated a “rolling submission” to the FDA.^52^ Importantly, though, according to some sources, Merck’s supply of clinical grade *r*VSV-ZEBOV has not kept pace with public health needs to address the outbreak in the Congo as the company has thus far not been able to produce the vaccine at its industrial scale facility.^53^

## The Security to Experiment with New Modes of Knowledge Production

Our analysis of *r*VSV-ZEBOV illustrates that the conventional wisdom of licensing out patents to the private sector for the purpose of commercialization permeates the furthest corners of public sector science. Even in the global health research context where there is little market incentive to develop a pharmaceutical intervention^54^, control over a promising and now-approved vaccine is transferred to a company on the strength of the assumption that the company will be better positioned to bring it to market. Yet, the development of *r*VSV-ZEBOV suggests otherwise: the NML performed the bulk of the experimental work and problem-solved various technical challenges encountered by IDT in the manufacturing process, from quality assurance to characterizing the vaccine’s toxicity, potency, and immunogenicity, and producing cGMP-grade lots of the vaccine. Beyond advising NML scientists about the FDA’s standards, BPS did little to advance *r*VSV-ZEBOV prior to the 2014–2015 epidemic.

At the same time, the public sector’s invaluable contributions to *r*VSV-ZEBOV’s development occurred under precarious conditions. The CSSP funding for the project took years to secure only to be later undercut by a change in federal government support for civil servant scientists. The inventors of *r*VSV-ZEBOV all departed the NML by 2010, leaving Alimonti—herself precariously employed—to run the project with minimal institutional support.

These two aspects of *r*VSV-ZEBOV’s development are mutually constitutive. Public sector institutions such as the NML struggle to identify private sector partners with the capacity to further the development of promising products. The licensing agreement was struck with BPS, which had no track record of bringing a product to market. And when the partners (BPS and IDT Biologika) failed to perform as promised, the public sector lacked the capacity to enforce the terms of the contract. The NML’s decisions were, in other words, clouded by the conventional logic of commercialization. And, just as that logic inspires licensing patent rights out to the private sector, it renders precarious funding and labor in public sector science while simultaneously obscuring the actual contributions of publicly funded research to therapeutic interventions such as *r*VSV-ZEBOV. This is how commercialization practice works to entrench the status quo.

To disrupt this pattern, alternative approaches to knowledge production and diffusion, particularly in the context of global health where market interest is often lacking, are needed; so too must the state better support publicly funded institutions and scientists to pursue translational research. That is, rather than assume that the only way to advance vaccines and other products is by patenting and licensing to the private sector, government laboratories, funding agencies, and universities ought to consider a broader range of options.

One option, which is gaining interest, is to create a “public option,” that is, a public sector led supply of essential, affordably priced medicines from discovery all the way through manufacturing and regulatory approval.^55^ Indeed, the case of *r*VSV-ZEBOV shows that such a public option is not simply theoretical; rather, as demonstrated by the NML, the public sector has the capacity to do much more than pure discovery research. Armed with sufficient resources, public sector labs can conduct phase 1, 2, and 3 clinical trials as well as solve many of the technical challenges that manufacturing a clinical grade vaccine will entail.

Pursuing such an alternative approach to research and development can begin with changing *how* the work of science is done. Here, open science collaborations, where publications and data are widely shared without restrictions imposed by intellectual property, appear promising. Rather than supplying an additional market reward, open science collaborations change the way that science is practiced and knowledge is generated^56^. The global influenza research network, which succeeded in producing the information needed to generate seasonal influenza vaccines for decades, stands as a powerful example of high-cost, high-risk open science.^57^ Other innovative research models exist in the neglected disease realm as well^58^. Given the modest outcomes from relying on incentives to industry as observed in the case of *r*VSV-ZEBOV, secure funding and employment for public sector scientists to experiment with these new modes of knowledge production may prove a far better investment.

While the high prices of many publicly funded and developed drugs and vaccines continue to command policy attention, it is critical that we attend to the ways in which the standard logic of commercialization and the division of scientific labor that it commands as between the public and private sectors tends to reproduce the status quo. Unless the norms and practices of knowledge generation begin to change, the contributions and capacity of public sector science to the development of important health interventions like *r*VSV-ZEBOV will continue to be under-realized and, if and when interventions result, subject to pricing abuse.

## Supplementary Material

JLB_Ebola_submission_Supp_File_FINAL_lsz019

## Footnotes

1 Ashley J. Stevens et al., *The Role of Public-Sector Research in the Discovery of Drugs and Vaccines*, 364 N.E.J.M. 535 (2011); Ekaterina G. Cleary et al., *Contribution of NIH Funding to New Drug Approvals 2010–2016*, 115 P.N.A.S., 2329 (2018); Aaron S. Kesselheim et al., *The Roles of Academia., Rare Diseases, and Repurposing in the Development of the Most Transformative Drugs*, 34 HEALTH AFFAIRS 286 (2015).

2 Christopher Rowland, *An HIV Treatment Costs Taxpayers Millions. The Government Patented It. But A Pharma Giant Is Making Millions*, WASHINGTON POST (March 26, 2019), https://www.washingtonpost.com/business/economy/pharma-giant-profits-from-hiv-treatment-funded-by-taxpayers-and-patented-by-the-government/2019/03/26/cee5afb4-40fc-11e9-9361-301ffb5bd5e6_story.html (accessed Sep. 9, 2019).

3 Mariana Mazzucato, THE ENTREPENURIAL STATE (New York: Anthem Press, 2014); Mark A. Lemley, *Ex Ante Versus Ex Post Justifications for Intellectual Property*, 71 U. CHI. L. REV. 129 (2004); Janice E. Graham, *Ebola Vaccine Innovation: A Case Study of Pseudoscapes in Global Health*, 29 CRITICAL PUB. HEALTH 401 (2019).

4 See Supplementary File—Methods.

5 See Supplementary File—BPS Agreement.

6 Ewen Callaway, ‘Make Ebola a thing of the past’: first vaccine against deadly virus approved, Nature (Nov. 12, 2019), https://www.nature.com/articles/d41586-019-03490-8 (accessed Nov. 25, 2019). The vaccine was also submitted by Merck to the US Food and Drug Administration and subsequently approved. See Merck, *Merck Begins Rolling Submission of Licensure Application for V920 (rVSV∆G-ZEBOV-GP) to U.S. Food and Drug Administration* (Nov. 13, 2018), https://investors.merck.com/news/press-release-details/2018/Merck-Begins-Rolling-Submission-of-Licensure-Application-for-V920-rVSVG-ZEBOV-GP-to-US-Food-and-Drug-Administration/default.aspx (accessed Sep. 9, 2019); Shelby Lin Erdman, ‘FDA approves first-ever vaccine for prevention of Ebola virus’, CNN health (Dec. 20, 2019), https://www.cnn.com/2019/12/20/health/fda-approves-first-ever-vaccine-ebola-virus/index.html (accessed Dec. 29, 2019).

7 Joseph R. A. Fitchett et al., *Ebola Research Funding: A Systematic Analysis, 1997–2015*, 6 J. GLOB. HEALTH, 020703 (2016).

8 MELANIE ARMSTRONG, GERM WARS: THE POLITICS OF MICROBES AND AMERICA’S LANDSCAPE OF FEAR (Oakland, California: University of California Press, 2017).

9 Government of Canada, *Defence Research and Development Canada, Foresight Helped Make Canada a World Leader in Ebola Research* (Sep. 10, 2014), http://www.drdc-rddc.gc.ca/en/dynamic-article.page?doc=foresight-helped-make-canada-a-world-leader-in-ebola-research/hzvlqma8 (accessed Sep. 9, 2019).

10 Heinz Feldmann et al., *Ebola Virus: From Discovery to Vaccine*, 3 NAT. REV. IMMUN. 677 (2003).

11 Public Servants Inventions Act, RSC 1985, c P-32.

12 U.S. Patent No. 8,012,489. Recombinant Vesicular Stomatitis Virus Vaccines for Viral Hemorrhagic Fevers (published February 5, 2004).

13 STAFF OF S. COMM. ON THE JUDICIARY, 85TH CONG., SUBCOMM. ON PATENTS, TRADEMARKS, & COPYRIGHTS, STUDY 15: *An Economic Review of the Patent System* (Comm. Print 1958 prepared by Fritz Machlup); Edmund W. Kitch, *The Nature and Function of the Patent System*, 20 J.L. & ECON. 265 (1977).

14 Michael Garbutt et al., *Properties of Replication-Competent Vesicular Stomatitis Virus Vectors Expressing Glycoproteins of Filoviruses and Arenaviruses*, 78 J. VIROL. 5458 (2004); Steven M. Jones et al, *Live Attenuated Recombinant Vaccine Protects Nonhuman Primates against Ebola and Marburg Viruses*, 11 NATURE MED. 786 (2005); Heinz Feldmann et al, *Effective Post-Exposure Treatment of Ebola Infection*, 3 P.L.O.S. PATHOGENS e2 (2007).

15 See Supplementary File—Project Completion Report.

16 See Supplementary File—BPS Development Plan.

17 See Supplementary File—BPS Development Plan.

18 See Supplementary File—PHAC License Negotiation Term Sheet.

19 See Supplementary File—BPS Project Updates A.

20 See Supplementary File—BPS Agreement.

21 NewLink Genetics Corp., *Sole License Agreement for Recombinant Stomatitis Virus Vaccines for Viral Hemorrhagic Fevers* (2010), https://www.sec.gov/Archives/edgar/data/1126234/000104746911009169/a2206169zex-10_67.htm (accessed Sep. 9, 2019).

22 NewLink Genetics Corp., *Form 10-K* (Dec. 31, 2011), https://www.sec.gov/Archives/edgar/data/1126234/000112623412000014/nlnk-20111231x10k.htm (accessed Sep. 9, 2019).

23 It is worth noting that there are important historical exceptions to today’s practice of not stipulating a commitment to ‘reasonable pricing’ in patent licensing agreements. See, for e.g., Maurice Cassier & Christiane Sinding, *‘Patenting in the Public Interest:’ Administration of Insulin Patents by the University of Toronto,* 24 Hist. & Tech. 153 (2008).

24 Pursuant to Standing Order 32(2), Mr. Van Loan (Leader of the Government in the House of Commons) laid upon the Table,—Answer to question Q-1155 on the Order Paper.—Sessional Paper No. 8530–412-25.

25 Qiu Xiangguo et al., *Sustained Protection against Ebola Virus Infection Following Treatment of Infected Nonhuman Primates with ZMAb,* 3 SCIENTIFIC REPORTS 3365 (2013).

26 Allain J. Barnett & Melanie G. Wiber, *What Scientists Say about the Changing Risk Calculation in the Marine Environment under the Harper Government of Canada (2006–2015),* 44 S.T. & H.V. 29 (2018). See also Supplementary File—Project Completion Report.

27 See Supplementary File—Project Completion Report.

28 See Supplementary File—Project Completion Report.

29 See Supplementary File—Key Emails A, Project Updates B.

30 See Supplementary File—Key Emails B.

31 See Supplementary File—Key Emails C.

32
*Sessional Paper*, *supra* note 24.

33 See Supplementary File—Project Completion Report.

34 U.S. Dep’t of Health and Human Servs. et al., *Guidance for Industry: CGMP for Phase 1 Investigational Drugs* (July 2008), https://www.fda.gov/downloads/drugs/guidances/ucm070273.pdf (accessed Sep. 9, 2019). See also Supplementary File—Project Completion Report.

35 See Supplementary File—Project Updates C.

36 See Supplementary File—Key Emails D, Project Updates C.

37 See Supplementary File—Key Emails E, Project Updates D.

38 World Health Org., *Origins of the 2014 Ebola Epidemic* (Jan. 2015), https://www.who.int/csr/disease/ebola/one-year-report/virus-origin/en (accessed Sep. 9, 2019).

39 Thomas Walkom, *The Strange Tale of Canada’s Ebola Vaccine*, TORONTO STAR (Nov. 25, 2014), https://www.thestar.com/news/canada/2014/11/25/the_strange_tale_of_canadas_ebola_vaccine_walkom.html (accessed Sep. 9, 2019).

40 Denise Grady, *Ebola Vaccine, Ready for Test, Sat on the Shelf,* N.Y.T. (Oct. 23, 2014), https://www.nytimes.com/2014/10/24/health/without-lucrative-market-potential-ebola-vaccine-was-shelved-for-years.html (accessed Sep. 9, 2019); Amir Attaran & Jason W. Nickerson, *Is Canada Patent Deal Obstructing Ebola Vaccine Development?* 384 THE LANCET e61 (2014).

41 NewLink Genetics Corp., *Exhibit 10.105, License and Collaboration Agreement* (2014), https://www.sec.gov/Archives/edgar/data/1126234/000112623415000054/nlnk-20141231xex10105.htm (accessed Sep. 9, 2019).

42 The National Academies of Sciences, Engineering, Medicine*, Integrating Clinical Research into Epidemic Response: The Ebola Experience* (Apr. 12, 2017), (http://nationalacademies.org/hmd/Reports/2017/integrating-clinical-research-into-epidemic-response-the-ebola-experience.aspx (accessed Sep. 9, 2019).

43 May S. ElSherif et al., *Assessing the Safety and Immunogenicity of Recombining Vesicular Stomatitis Virus Ebola Vaccine in Heatlhy Adults: A Randomized Clinical Trial*, 189 CAN. MED. ASSOC. J. e819 (2017).

44
*Exhibit 10.105*, *supra* note 22.

45 Ana Maria Henao-Restrepoet et al., *Efficacy and Effectiveness of an Rvsv-Vectored Vaccine in Preventing Ebola Virus Disease: Final Results from the Guinea Ring Vaccination, Open-Label, Cluster-Randomised Trial (Ebola Ça Suffit!),* 389 THE LANCET 505 (2016). It is important to note that the number of participants reported here (11,841) encompasses all the individuals whom the WHO-led consortium recruited for potential enrollment in the trial. As explained in the published version of the trial, the total number of participants that were found eligible to enroll (8334) or actually gave consent to take part in the trial (6367) was substantially lower. Further, these numbers also differ from the number of participants reported in Table 2 below for the same *Ebola Ça Suffit!* trial. However, inconsistencies between published clinical studies and data reported in Clinicaltrials.gov (the source for all of the data in Table 2) are common, and the number of participants eligible to enroll (8334) is relatively close to the number of participants reported on Clinicaltrials.gov (8851).

46 Merck has received funding from multiple government agencies in the US and Europe. See for e.g., Innovative medicines initiative, Europe’s partnership for health. *VSV-EBOPLUS: Vaccine safety and immunogenicity signatures of human responses to VSV-ZEBOV*, https://www.imi.europa.eu/projects-results/project-factsheets/vsv-eboplus (accessed Sep. 16, 2019).

47 Gavi, *Ebola Vaccine Purchasing Commitment from Gavi to Prepare for Future Outbreaks* (Jan. 2016), https://www.gavi.org/library/news/press-releases/2016/ebola-vaccine-purchasing-commitment-from-gavi-to-prepare-for-future-outbreaks/ (accessed Sep. 9, 2019).

48 Department of Health and Human Services, *HHS Accelerates Development of First Ebola Vaccines and Drugs* (Sep. 29, 2017), https://www.hhs.gov/about/news/2017/09/29/hhs-accelerates-development-first-ebola-vaccines-and-drugs.html (accessed Sep. 9, 2019).

49 Gavi, *supra* note 47.

50 Eric Sagonowsky, *Merck Will Delay Filing Ebola Vaccine for Approval until 2018, Company Confirms*, FIERCEPHARMA (Nov. 21, 2017), https://www.fiercepharma.com/vaccines/merck-to-miss-2017-filing-target-for-ebola-vaccine-spokesperson (accessed Sep. 9, 2019).

51 John Gever, *Still no clarity on Merck’s Ebola vax in Congo*, Medpage Today (Oct. 30, 2018), https://www.medpagetoday.com/meetingcoverage/astmh/76018 (accessed Oct. 7, 2019).

52 Merck, *supra* note 6.

53 Jenny Lei Ravelo, *Merck to make more Ebola vaccine, WHO declines to declare global emergency,* devex (Jun. 14, 2019), https://www.devex.com/news/merck-to-make-more-ebola-vaccine-who-declines-to-declare-global-emergency-95114 (accessed Sep. 17, 2019).

54 Patrice Trouiller et al., *Drugs for Neglected Diseases: A Failure of The Market and A Public Health Failure?* 6 TROP. MED. INT. HEALTH 945 (2001).

55 Dana Brown, *Medicine for All: The Case for a Public Option in the Pharmaceutical Industry*, Democracy Collaborative (Sep. 2019), https://docs.google.com/viewerng/viewer?url=https://thenextsystem.org/sites/default/files/2019-09/MedicineforAll_WEB.pdf (accessed Sep. 17, 2019).

56 E. Richard Gold, *Accelerating Translational Research Through Open Science*, 14 P.L.O.S. BIOL. e2001259 (2016).

57 Amy Kapczynski, *Order Without Intellectual Property Law: Open Science in Influenza*, 102 CORNELL L. REV. 1539 (2017).

58 Michael P. Pollastri, *Finding New Collaboration Models for Enabling Neglected Tropical Disease Drug Discovery*, 8 P.L.O.S. e2866 (2014).

