# Supplementary material for: From discovery to delivery: public sector development of the rVSV-ZEBOV Ebola vaccine
Source: J Law Biosci. 2020 Jan 16;7(1):lsz019. doi: 10.1093/jlb/lsz019 (PMC8249092; doi:10.1093/jlb/lsz019)
Supplement: JLB_Ebola_submission_Supp_File_FINAL_lsz019 [file jlb_ebola_submission_supp_file_final_lsz019.pdf]

SUPPLEMENTARY FILE

METHODS

From Discovery to Delivery:
Public Sector Development of *r*VSV-ZEBOV Ebola Vaccine

Supplementary File - Methods

Herder and Gold submitted the following request under Canada's *Access to Information Act* in December 2014:

> "All records concerning any agreements or sub-agreements between the Government of Canada and any private sector company, including but not limited to BioProtection Systems Corporation, in respect of all sole or other licences for recombinant vesicular stomatitis virus vaccines for viral hemorrhagic fevers, including in particular all studies, reports and related records arising from any agreements or sub-agreements in the period from 2000 to the present."

In response, we received a disclosure package in March 2016 totalling 1,157 pages of internal correspondence, emails between government officials and outside parties, technical materials pertaining to *r*VSV-ZEBOV, and various other documents related to the licensing of the vaccine. While elements of the documents were redacted in accordance with Canada's Access to Information Act to protect personal information, trade secrets, and confidential business information, this would not have covered disclosure of the fact that any process, report or negotiation were underway. We assumed that the 1,157-page "2016 disclosure package" contained any and all records within the scope of our Access to Information Act request.

The 2016 disclosure package was initially reviewed by two research assistants under the close supervision of Gold. The research assistants generated an Excel spreadsheet that served as roadmap for further analysis of the disclosure by creating a chronological list of the

documentation, and pertinent information in the documentation such as the names of individuals involved in correspondence, the subject of the correspondence, and so forth. The spreadsheet was used by Herder, Gold, and Graham to carry out an in-depth analysis of the licensing negotiations and subsequent enforcement of the contract in comparison to the public record of events.

We complemented our analysis of the disclosure package with a search of disclosure documents available through the US Securities and Exchange Commission, including annual and quarterly reports and disclosures of contract terms. Herder, Graham and Gold also reviewed *Hansard* and other public records of Parliament of Canada debates, questions and answers. Finally, we conducted web searches and accessed scholarly publications in respect of the individuals and firms involved in the case study.

In addition, one of us (Graham) served in the role of Associate Director of the Canadian Center for Vaccinology of Dalhousie University, where the phase 1 Canadian trial was carried out. She also participated in a meeting organized by the World Health Organisation in Veyrier de Lac in January 2015 entitled “Ebola Vaccine: where are we? how to move forward?” This allowed Graham to have in depth conversations with researchers and global health officials where she gained substantial knowledge of the manufacturers and trials being planned. Further, as a member of the scientific advisory group for the Guinea Ebola vaccine trials, Graham was involved in multiple meetings with those who designed and carried out the trials. These qualitative details acted as data that guided analysis along with the disclosure package and the published literature.

In October 2018, one of us (Herder) was contacted by the Scientific Director General of the National Microbiology Laboratory (NML) in Winnipeg, Manitoba, where the rVSV-ZEBOV was

discovered, developed, and licensed to BioProtection Systems Corporation. The Director General learned of our article during the peer review process. He informed us that a number of documents in the possession of NML had, through an administrative error, not been disclosed to us in response to our original 2014 Access to Information Act request. A second disclosure package, totalling 479 pages and comprised of external and internal correspondence, key agreements, key reports, and key presentations, was therefore delivered to us in electronic and hard copy formats.

The “2018 disclosure package” was analysed by one research assistant as well as all three authors. The resulting article is based upon an analysis of both the 2016 and 2018 disclosure packages. The remainder of this Supplementary File is comprised of key documents, either contained in the disclosure packages or the public domain, that we refer to in the course of our analysis. Complete copies of both the 2016 and 2018 disclosure packages are available upon request.

BPS AGREEMENT

EX-10.67 24 a2206169zex-10_67.htm EX-10.67

Exhibit 10.67

CERTAIN CONFIDENTIAL INFORMATION CONTAINED IN THIS DOCUMENT, MARKED BY BRACKETS, HAS BEEN OMITTED AND FILED SEPARATELY WITH THE SECURITIES AND EXCHANGE COMMISSION PURSUANT TO RULE 406 OF THE SECURITIES ACT OF 1933, AS AMENDED.

SOLE LICENSE AGREEMENT
FOR
RECOMBINANT VESICULAR STOMATITIS VIRUS VACCINES FOR VIRAL HEMORRHAGIC FEVERS

BETWEEN:

HER MAJESTY THE QUEEN IN RIGHT OF CANADA,
as represented by the Minister of Health,
acting through the Public Health Agency of Canada

("Canada")

AND:

BIOPROTECTION SYSTEMS CORPORATION,
a company incorporated as a subchapter C corporation under the laws of Delaware, having its registered office at Iowa State University Research Park, 2901 South Loop Drive, Suite 3360, Ames, Iowa, USA 50010

("Company")

INTRODUCTION:

A. **WHEREAS** Canada is one of the major performers in Canada of vaccine research relating to viral hemorrhagic fever ("*VHF*") viruses;

B. **WHEREAS** Canada has developed the technology known as the "Recombinant vesicular stomatitis virus vaccine for viral hemorrhagic fevers";

C. **WHEREAS** the main features of the technology include [*];

D. **WHEREAS** the Company has requested a license from Canada to develop and ***Commercialize*** the technology;

E. **WHEREAS** Canada is willing to grant to the Company a license to develop and ***Commercialize*** the technology on the terms and conditions set out in this ***License Agreement***;

F. **WHEREAS** the fundamental principles underlying this ***License Agreement*** are that:

i) Canada surrenders its commercial self-interest to the Company; and

ii) In exchange, in good faith, the Company uses its discretion and experience in product development and regulatory affairs, its commercial resources and business savvy and, assuming that any relevant statutory, regulatory or administrative authorizations or permits for a vaccine product are obtained, its marketing, sale and distribution savvy for the benefit of both ***Parties***.

G. **WHEREAS** the salient elements of this ***License Agreement*** are:

i) Canada grants to the Company sole, worldwide, revocable and royalty-bearing license to make, use, improve, develop and ***Commercialize*** the technology in the field of prevention and prophylaxis against and treatment of VHF viruses in humans, whether before or after exposure;

ii) Canada will retain non-commercial rights in the technology, including rights to use and further develop the technology for educational and research purposes;

iii) The Company grants to Canada a non-exclusive and royalty-free license to make, use, manufacture and sell the VHF vaccine products developed by the Company in the exercise of the ***Licensed Rights***, in the event of a public health emergency:

iv) The Company will make good faith efforts to collaborate with Canada on [*] of the Company's basic research and development activities related to *VHF* virus vaccines; and

v) The ***Parties*** agree to maintain the confidentiality of each other's ***Confidential Information*** provided under this ***License Agreement***.

H. **WHEREAS** the expectations of the Parties are that the Company will use commercially reasonable efforts to develop a *VHF* vaccine and, assuming that any relevant and necessary statutory, regulatory and administrative authorizations or permits that may be required for a vaccine product are obtained, ***Commercialize*** it; and

I. **WHEREAS** the Parties have agreed to their commercial relationship on the terms and conditions set out in this ***License Agreement***.

NOW THEREFORE in consideration of the premises, the terms and conditions hereinafter contained and other good and valuable consideration, the receipt of which is hereby acknowledged by each party, the Parties hereto covenant and agree as follows:

1.0 DEFINITIONS

1.1 "Affiliate"

means any corporation, subsidiary, partnership or other entity which the Company, directly or indirectly, controls (or has common control of) or which, directly or indirectly, controls the Company:

1.1.1 through the ownership of more than 50% of the voting share capital, and the votes attached to those securities are sufficient, if exercised, to elect a majority of the directors of the body corporate; or

1.1.2 otherwise has the possession, direct or indirect, of the powers to direct or cause the direction of the management or policies of a person or entity; whether through ownership of equity participation, voting securities, or beneficial interests; by contract, by agreement, or otherwise.

Identified in appendix D ("Affiliates") are the ***Affiliates*** of the Company In existence on the ***Execution Date***.

1.2 "Commercialization" or "Commercialize"

means:

1.2.1 the commercial making, using, ***Sale*** or offering to sell;

1.2.2 of the products resulting from the exercise of the ***Licensed Rights***;

1.2.3 by the Company, its ***Affiliates*** or its sub-licensees;

1.2.4 in the ***Territory***;

1.2.5 within the ***Field of Use***; and

1.2.6 for the maximum commercial return to the Company and Canada in accordance with Article 4 (Exploitation of ***Licensed Rights***) including:

1.2.6.2 the Company obtaining any statutory, regulatory or administrative authorizations or permits that may be required in order for the Company to legally carry out all of its activities under the ***License Agreement***.

1.3 "Confidential Information"

means, with respect to a ***Party***, all proprietary information of any type, or any part or portion thereof, that is disclosed by that ***Party*** to the other ***Party*** pursuant to this ***License Agreement***, whether or not such information is specifically marked or identified as confidential at the time of disclosure, which may include without limitation.

1.3.1 all scientific, technical, business, financial, legal, marketing or strategic information (including trade secrets and proprietary know-how);

1.3.2 all documented research, development, demonstration or engineering work, information that can be or is used to define a design or process or procure, produce, support or operate material and equipment, methods of production, regardless of its form:

1.3.3 all drawings, blueprints, patterns, plans, flow-charts, equipment, parts lists, software and procedures, specifications formulae, designs, technical data, descriptions, related instruction manuals, records and procedures;

1.3.4 information that is non-public, confidential, privileged or proprietary in nature.

which may have actual or potential economic value in part from not being known and may be positive (what works) or negative (what does not) information;

1.3.5 however fixed, stored, expressed or embodied (and includes, without limitation, samples, prototypes, specimens and derivatives);

1.3.6 and including information disclosed during discussions, meetings, tests, demonstrations, correspondence or otherwise.

1.4 "Confidentiality Agreements"

means the agreements previously executed between the ***Parties*** on the 1st day of May, 2007, November 1, 2008, and the amending letter of April 14, 2010 respectively, and contained in Appendix B (***Confidentiality Agreements***).

1.5 "Dispute"

for purposes of Article 16 (Alternate Dispute Resolution (ADR)), and paragraph 17.17 (Forum Conveniens)

1.5.1 includes without limitation any controversy, conflict, claim, disagreement or difference of opinion arising out of the ***License Agreement***, (irrespective of whether it is premised on contract, tort or trust / equity), including, without limitation, any issues concerning the breach, interpretation, rectification, termination, performance, enforcement or validity of the ***License Agreement*** or the rights and liabilities of the ***Parties*** in relation to the ***License Agreement***;

1.5.2 irrespective of the fact that there is no arguable defence under the ***License Agreement***, or that the facts or law are undisputable and subject to judicial summary proceedings;

but ***Dispute*** does not encompass

1.5.3 any controversy, conflict, claim, disagreement or difference of opinion or the rights and liabilities of the ***Parties***

1.5.3.2 under any collateral or antecedent agreements independent of the ***License Agreement***; or

1.5.3.3 with any emanation of Her Majesty the Queen in Right of Canada, other than the Public Health Agency of Canada.

1.6 "Execution Date"

means the date on which the last signature is affixed to this ***License Agreement***.

1.7 "Field of Use"

means the application and use of the ***Licensed Rights*** only with products to be sold or used by the Company, or its ***Affiliates*** or sublicensees or marketed through

specified trade channels in the field of prevention and prophylaxis against and treatment of VHF viruses in humans and for no other purposes whatsoever.

1.8 "Generally Accepted Accounted Principles (GAAP)"

means, at any time, accounting principles generally accepted in Canada as recommended in the Handbook of the Canadian Institute of Chartered Accountants at the relevant time, applied on a consistent basis (except for necessary or advisable changes in accordance with the promulgations of the Canadian Institute of Chartered Accountants). If and when Canadian GAAP does not address an accounting issue, then generally accepted accounting principles in the United States will apply.

1.9 "Improvement(s)"

means any modification, improvement, enhancement, variation, refinement, derivative or development relating to the ***Licensed Rights*** which

1.9.1 infringes any one or more claims of any of the ***Patents***; or

1.9.2 constitutes a technological advance of any degree using any of the ***Patents*** or ***Confidential Information*** (irrespective of whether it infringes one or more claims of the ***Patents***); and

1.9.3 was made and reduced into practice during the term of the ***License Agreement*** or within 12 (twelve) months of its termination or expiration by either ***Party***; and

1.9.4 when applicable, Canada is lawfully entitled to communicate and license to the Company without breaching any restrictions on use or disclosure to third parties.

1.10 "Intellectual Property"

means, as of the ***Execution Date***, all ***Patents***, trade-marks, copyrights, industrial designs, trade-names, trade secrets, ***Confidential Information*** and other intellectual property rights whether registered or not, whether proprietary or not

i) owned by or licensed to Canada, relating to the ***Licensed Rights***; or

ii) owned by or licensed to the Company, relating to the ***Improvements*** made by the Company, its ***Affiliates*** or sub-licensees, as the case may be.

1.11 "License Agreement"

means this agreement and including all attached appendices and future amendments, and refers to the whole of this agreement, not to any particular section or portion thereof.

1.12 **"Licensed Product(s)"**

means any product resulting from ***Commercialization*** under this ***License Agreement***.

1.13 "Licensed Rights"

means the exercise, as of the ***Execution Date***, in whole or in part, of

1.13.1 the ***Patents***; and

1.13.2 related ***Intellectual Property*** and ***Confidential Information*** and any subsequent changes thereto that are expressly incorporated into the ***License Agreement***,

within the ***Field of Use*** as listed in Appendix A (Description of the ***Licensed Rights***).

1.14 "Party"

means any one of the signatories to the ***License Agreement*** and "***Parties***" means both of them.

1.15 "Patents"

means

1.15.1 the patents and patent applications as listed in Appendix A (Description of the ***Licensed Rights***);

1.15.2 any author certificates, inventor certificates, utility certificates, improvement patents and models and certificates of addition, and includes any divisions, reissues, renewals, reexaminations and extensions thereof, and all continuations, continuations-in-part and divisionals of the applications for such patents, continuations, continuations-in-part, extensions, re-issues thereof for such patents, including, but not limited to, those patents listed in "Appendix A" and any subsequent patents whose priorities are derived from any patents listed in Appendix A; and

1.15.3 subsequently patented ***Improvements*** to ***Patents***.

1.16 "Sale"

means without limitation the act of transferring (conditionally or unconditionally, permanently or temporarily) the results of the exercise of the ***Licensed Rights*** for consideration including but not limited to sale, lease, gift, barter, exchange or other

disposition for value. (For greater clarity any internal corporate use / consumption whatsoever of the ***Licensed Rights*** by the Company or an ***Affiliate*** or sublicensee shall be deemed a ***Sale*** at the ***Sales Price*** at the lime of the use / consumption or allocation for internal use / consumption, whichever is the earlier).

1.17 "Sales Price"

means the aggregate gross price paid by an arm's length purchaser or lessee for any of the results of the exercise of the ***Licensed Rights*** sold or leased by the Company without deduction, rebate or pass throughs. If the gross price is less than the fair market value, then, for royalty calculation purposes, the gross price shall be the fair market value as set by Canada in its unfettered discretion.

1.18 "Taxes"

means taxes (including, without limitation, sates taxes, goods & services taxes, value added taxes, however described), levies, imposts, deductions, charges, license and registration fees, assessments, withholdings / withholding taxes and duties imposed by any jurisdiction or authority (including stamp and transaction taxes and duties) together with any related interest, penalties, fines and expenses in connection with them.

1.19 "Territory"

means the entire world, always subject to:

1.19.1 the United Nations Act, R.S.C. 1985, Chap. U-2:
1.19.2 the Export & Import Permits Act, R.S.C. 1985;
1.19.3 Chap. E-19, Special Economic Measures Act, S.C. 1992, Chap. 17;
1.19.4 Foreign Extra-Territorial Measures Act, R.S.C. 1985 c. F-29; and
1.19.5 any other pertinent Canadian statutory or regulatory strictures.

For greater clarity ***Territory*** means all countries and jurisdictions of the world.

1.20 "VHF"

means viral hemorrhagic fevers.

2.0 GRANT & RESERVATIONS

2.1 Grant:

Subject to:

2.1.1 the definitions, terms and conditions of the ***License Agreement***,

2.1.2 the Company complying with and not being in breach of any of the provisions of the ***License Agreement***, and

2.1.3 any third party peremptory rights,

Canada hereby grants to the Company a personal, non-transferable, sole, revocable, royalty-bearing license for ***Commercialization***.

Nothing herein shall constitute in any manner whatsoever:

2.1.4 an assignment or other transfer of proprietary rights in the ***Licensed Rights*** to the Company; or

2.1.5 any authorization or permission beyond that expressly given in this ***License Agreement***.

2.2 Carve Out

Notwithstanding anything to the contrary in the ***License Agreement***, Canada retains from the ***License Agreement***, any and all absolute and unfettered rights necessary to do the following:

2.2.1 improve the ***Licensed Rights*** or ***Patents***;

2.2.2 to carry out educational activities;

2.2.3 to pursue research and development, directly or indirectly, related to the ***Licensed Rights*** or ***Patents*** with or without the Company, collaborators or sponsors, with all attendant rights of publication;

2.2.4 to make, have made, manufacture, use, license sell and distribute and to administer (directly or through health care providers) to Canadians products resulting from the exercise of the ***Licensed Rights***, the ***Patents*** and the ***Improvements*** in the event of a public health emergency pertaining or related to *VHF* in Canada, for the purpose of prevention or treatment of *VHF*, where:

2.2.4.2 the Company has not obtained regulatory approval of its product(s) under the Food and Drugs Act of Canada at the time the emergency is identified by Canada; or

2.2.4.3 the Company is not able to satisfy the demand for its approved product(s) in Canada at the time the emergency is identified by Canada;

2.2.5 to make, have made, manufacture, use and distribute and to administer to Canada's staff products resulting from the exercise of the ***Licensed Rights***, the ***Patents*** and the ***Improvements***, for the purpose of prevention and treatment of *VHF*, whether in or outside a public health emergency in Canada or abroad, and

2.2.6 to make, have made, manufacture, use, license, sell and distribute and to administer (directly or through health care providers) products resulting from the exercise of the ***Licensed Rights***, the ***Patents*** and the ***Improvements***, outside of

Canada, for compassionate care purposes for the prevention or treatment of *VHF*, where:

2.2.6.2 the Company has not obtained regulatory approval of its product(s) under the laws of the foreign country in question at the time the compassion care is identified by Canada; or

2.2.6.3 the Company is not able to satisfy the demand for its approved products) in the foreign country in question at the time the compassionate care is identified by Canada.

2.3 Non Compete by Canada

Subject to clause 2.2, Canada shall not commercially compete with the Company, or grant a license to any third party for commercial purposes, within the ***Field of Use*** concerning the ***Licensed Rights*** in the ***Territory***.

2.4 Sublicensing Permitted

The Company is permitted to sub-license ***Affiliates*** and non-affiliated or non-controlled parties, on the same terms and conditions of this ***License Agreement***. The Company has no right to encumber any contractual, legal or equitable rights the Company may have against any ***Affiliate*** or sub-licensee in favour of any financial institution or any third party whatsoever.

2.5 Sublicensing Conditions

Any sub-license or any amendment to any sub-license granted by the Company to ***Affiliates*** and non-affiliated or non-controlled parties, shall:

2.5.1 be royalty-bearing, revocable, without the right to sub-sub-license, except with the prior written consent of Canada, which consent shall not be unreasonably withheld;

2.5.2 carry a royalty rate no less than that prescribed in the ***License Agreement***;

2.5.3 be only within the ***Territory*** or any portion thereof;

2.5.4 be only within the ***Field of Use*** or a subset thereof;

2.5.5 be subject to the same obligations and restrictions as those required of the Company under the ***License Agreement***;

2.5.6 be copied to Canada immediately following execution; and

2.5.7 not be a *de facto* assignment.

For greater clarity, Canada shall receive from the ***Affiliates*** and sub-licensees not less than the same amount of consideration Canada would have received from the Company,

had the Company conducted the ***Commercialization*** rather than the ***Affiliates*** or sub-licensees. The Company shall ensure that any monies owing to Canada from the ***Affiliates*** or sub-licensees are paid to Canada when due, and shall be liable for any such monies irrespective of whether or not the ***Affiliate*** or sub-licensee paid the Company.

2.6 Sub-Licensee Consideration

In addition to the royalties payable by the ***Affiliates*** and sub-licensees to Canada as contemplated in paragraph 2.5 (Sub-licensing Conditions), the Company shall also pay to Canada [*] paid by the ***Affiliates*** and sub-licensees to the Company.

2.7 Termination

Termination of the ***License Agreement*** shall also terminate any subsisting sub-licenses, but any consideration due or owing to Canada shall be paid promptly thereafter, and any and all unsatisfied obligations and rights shall subsist until satisfied.

3.0 TERM

3.1 Term

This ***License Agreement*** shall commence on the ***Execution Date*** and shall continue in force until the expiry of the last to expire of the ***Patents*** included in the ***Licensed Rights***, subject to:

3.1.1 early termination as prescribed under Article 15.0 (Termination); and

3.1.2 condition subsequent in paragraph 4.1 (Business Plan).

4.0 EXPLOITATION OF LICENSED RIGHTS

4.1 Business Plan

The Company shall submit a business plan to Canada within thirty (30) days of the ***Execution Date***. Canada shall have the right to request amendments to the business plan in order to ensure maximum commercial return to the Company and Canada in accordance with this Article 4 (Exploitation of Licensed Rights). Once Canada has accepted the business plan, the plan is then Appendix C (Business Plan) and all the Company's representations and statements in the plan are incorporated into the ***License Agreement***.

4.2 Disclosure Requirements

The business plan shall provide sufficient detail to show how the Company plans to diligently research, develop and promote and make commercially reasonable efforts to ***Commercialize***. This business plan shall also disclose any

4.2.1 distribution and agency arrangements contemplated by the Company;

4.2.2 market studies pertinent to the ***Licensed Rights***;

4.2.3 pro forma financial statements of sufficient detail to allow a thorough financial analysis of the Company's assumptions, projected revenue streams and costs.

4.3 Continuing Disclosure

During the term of the ***License Agreement***, the Company shall promptly provide to Canada any amendments or updates to the business plan.

4.4 Inducement

The Company acknowledges that the business plan as orally presented to Canada in a pre-contractual setting, and subsequently manifested in the written format under paragraph 4.1, as accepted by Canada is the major inducement for Canada to enter into the ***License Agreement*** on the terms and conditions prescribed herein.

4.5 Breach

If the Company

4.5.1 commits a misrepresentation, omission, concealment or incorrect statement of a material fact in the negotiations leading to the ***License Agreement*** in general or leading to or in the business plan in particular; or

4.5.2 breaches any representations or statements in the business plan,

then such failure is a material breach of the ***License Agreement*** which provides Canada with the discretionary election either to:

4.5.3 rescind the ***License Agreement*** and seek damages; or

4.5.4 maintain the ***License Agreement*** and seek damages alone.

4.6 Commercially Reasonable Efforts to Commercialize

As an inducement to Canada to enter into the ***License Agreement***, during the term (or the renewal) of the ***License Agreement***, the Company shall:

4.6.1 use commercially reasonable efforts to ***Commercialize***;

4.6.2 use commercially reasonable efforts to create and satisfy demand for the ***Licensed Rights***; and

4.6.3 not do, or assist anyone to do, anything inimical to the ***Commercialization***.

Payment of fees and royalties under Article 5 (Fees & Royalties) does not relieve the Company of its obligation under paragraph 4.6 (Commercially Reasonable Efforts to Commercialize).

4.7 Shelving a Fundamental Breach

Any "parking", "shelving" or other activity or inactivity concerning the ***Licensed Rights*** whereby the Company is not using its commercially reasonable efforts to diligently and aggressively ***Commercialize*** the ***Licensed Rights*** in the ***Territory***, is a fundamental breach of the ***License Agreement***.

4.8 Research Support Collaboration

In carrying out basic research and development activities concerning the ***Licensed Rights*** and *VHF* vaccine during the term of this ***License Agreement***, and any renewal thereof, the Company shall make good faith efforts to collaborate with Canada on [*] of such activities, under collaborative research agreements containing commercially reasonable terms and conditions as agreed to by the ***Parties*** at that time. Any payments made by the Company pursuant to such collaborations shall not diminish or affect the Company's obligation to pay fees and royalties under Article 5 (Fee and Royalties).

5.0 FEES AND ROYALTIES

5.1 Fees

The Company shall pay to Canada the following non-refundable lump sums:

5.1.1 PATENT FEES
[*], payable within thirty (30) calendar days of the ***Execution Date***, as a reimbursement of ***Patent*** costs incurred by Canada to date;

5.1.2 SIGNING FEE
[*], payable upon signing, as a non-creditable and non-refundable signing fee in consideration of the execution of the ***License Agreement***;

5.1.3 MILESTONE FEES [*] lump sum payable on the earlier of [*] or [*] years of the *Execution Date*, whichever comes first;
5.1.4 MILESTONE FEES [*] lump sum payable on the earlier of [*] or [*] years of the *Execution Date*, whichever comes first;
5.1.5 MILESTONE FEES [*] lump sum payable on the earlier of [*]or [*] years of

the *Execution Date*, whichever comes first;

5.1.6 MILEPOST FEES [*] lump sum payable on the earlier of [*] or [*] of the *Execution Date*, whichever comes first.

5.2 Royalty Percentage Rate

The Company shall pay to Canada a royalty rate of [*] of the ***Sales Price*** of ***Licensed Products*** sold by the Company, its ***Affiliate(s)*** or sublicensees.

5.2.1 The royalty rate shall be lowered to [*] if: a) an additional technology is required to ***Commercialize***; and b) the additional technology is actually licensed by the Company from a third party and the latter actually charges royalties to the

Company for such a license (as shown by documentation sufficient to establish the requirement and the actual license).

5.2.2 The rate shall be towered to [*] if: a) two (2) or more additional technologies are required to ***Commercialize***; and b) the additional technologies are actually licensed by the Company from one or more third parties and the latter actually charge royalties to the Company for such a license (as shown by documentation sufficient to establish the requirement and the actual license).

5.3 Minimum Royalty

Notwithstanding any other provision of the ***License Agreement***, the Company shall pay to Canada a minimum annual royalty of [*], payable on or before January during each year of the ***License Agreement***. Such amounts paid shall be creditable against royalties owed under clause 5.2 (Royalty Percentage Rate) and sub-license payments owed under clause 5.4 (Sub-Licensing Consideration) in the same year.

5.4 Sub-Licensing Consideration

The Company shall pay to Canada [*] paid by the ***Affiliates*** and the sublicensees to the Company. Such payments shall be over and above the royalty rate paragraph 5.2 (Royalty Percentage Rate) (whether or not such consideration was directly, indirectly or derivatively paid or provided) including without limitation any equity.

5.5 Sub-Licensee’s Fees

5.5.1 COLLECTION AND ENFORCEMENT BY THE COMPANY

The Company shall ensure that royalties payable to Canada from ***Affiliates*** and sub-licensees shall be remitted directly to the Receiver General for Canada, at the address provided in Article 20.1 (Notice). The Company shall take any necessary actions (at the Company's own cost) to collect, enforce and remit royalties or other consideration owing to Canada by the ***Affiliates*** and sub-licensees.

5.5.2 SUB-LICENSEE'S ARREARS PAID BY THE COMPANY

If an ***Affiliate*** or sub-licensee has royalties or other consideration owing to Canada under a sub-license for a period in excess of thirty (30) days, then the Company shall pay to Canada that amount owing within the next fourteen (14) days immediately following the aforementioned thirty (30) days.

5.6 Taxes

The Company shall pay ***Taxes*** at the applicable prevailing rates exigible on the Company's activities under the ***License Agreement***, including without limitation ***Commercialization*** or on the payment of royalties, including any withholding taxes that in the first instance are levied against Canada

5.7 Payment to Canada

Unless the ***License Agreement*** expressly provides otherwise, the Company shall pay any and all monies and consideration owing to Canada as follows:

5.7.1 TIME & MODE

quarterly, by cheque or money order, commencing on December 31, 2010 and thereafter on March 31, June 30, September 30 and December 31 of each year of this ***License Agreement***;

5.7.2 CURRENCY & ADDRESS

except for royalties generated from ***Commercialization*** within Canada, cheques for the payment of royalties shall be in U.S. funds (at the conversion rate stated in the Wall Street Journal on the day prior to the date payment is due) and made payable to the "Receiver General for Canada". The cheque(s) shall be sent to

Director, Intellectual Property Management & Business Development Public Health Agency of Canada
1015 Arlington Street, Suite 2420
Winnipeg, Manitoba, Canada
R3E 3R2;

5.7.3 ACCOMPANYING DOCUMENTATION

each cheque shall be accompanied by a statement bearing the name / identification of this ***License Agreement*** and the ***Licensed Rights***, and showing the period covered, the total sales, per country sales, the per country royalty applicable and the total royalty paid or consideration paid, as applicable.

5.8 Payments to Canada after Termination

The Company shall pay to Canada any consideration due and payable under the ***License Agreement***, whether incurred before termination or after, in accordance with Article 15 (Termination).

5.9 Payment after Expiry of Patents

The Company shall continue paying the amounts as prescribed in this Article, notwithstanding any impeachment proceedings, or the expiry, expungement or other nullification of the ***Patents***.

5.10 No Set-off

Notwithstanding any other provision of the ***License Agreement***, any consideration payable to Canada by the Company under the ***License Agreement*** is unconditional and non-cancelable. Further, the Company shall not have the right of set-off, deduct or counter-claim against any such consideration.

5.11 Accounting Approach

5.11.1 GAAP

The Company shall use GAAP in the calculation of consideration owing to Canada.

5.11.2 ACCRUAL

Royalties accrue on receipt of payment by the Company (or ***Affiliates*** or the sub-licensees) for the ***Licensed Rights***.

5.11.3 INTEREST OF OVERDUE ACCOUNTS

In the event the Company fails to make any payment under the ***License Agreement*** when due and payable, then interest on any unpaid amount shall accrue at a rate of four (4)% above the base rate of the Bank of Montreal, Toronto, from time to time in force during the period of non-payment.

5.11.4 OTHER BASIS FOR PAYMENTS

If the Company receives any lump sum or other payments, royalties (including royalty payments received from third parties), or any other income or consideration for, or in respect of the ***Commercialization*** of the ***Licensed Rights***, then the Company shall include such additional income in calculating the ***Sales Price***.

6.0 RECORDS AND AUDIT

6.1 Records Maintenance

The Company shall keep true and accurate records and maintain such records relating to ***Commercialization*** and all other obligations of the Company under the ***License Agreement*** during the term of the ***License Agreement*** and for ten (10) years following the termination or expiration of the ***License Agreement***.

6.2 Record Type

For greater clarity and without limiting the generality of the foregoing, records cited in paragraph 6.1 (Records) shall comprehensively address:

6.2.1 financial, business, manufacturing and technical support, including without limitation sales reports, inventory reports, subcontractor and distributor agreements, tax returns, catalogues, price lists, shipping records, invoice registers, invoices, financial statements and ledgers; and

6.2.2 quality standards and monitoring reports and records.

6.3 Records, Access to those held by Off Site Professionals

The Company irrevocably authorizes its independent accountants, KPMG LLP, to provide to Canada's independent accountants any information it may have with respect to the ***Commercialization.***

6.4 Audit Document Right

Upon the written request of Canada and with at least fifteen (15) calendar days prior notice, the Company shall permit an independent accountant, retained by Canada, to inspect all relevant records (whether held internally by the Company or at the offices of professional advisors or elsewhere) in order to ascertain the accuracy of such royalties, reports and ***Commercialization*** efforts. Such inspections shall be during business hours and in a manner that does not unduly disrupt the Company's business. The Company shall allow the accountant to make any necessary copies of the records that the independent accountant deems fit.

6.5 Audit Interview Right

In addition to the rights in paragraph 6.4, upon the written request of Canada, the Company shall allow the independent accountant to interview key personnel of the Company. The independent accountant, in its unfettered discretion, shall determine who the key personnel are for the purposes of the interview. The Company acknowledges that the independent accountant may have more than one interview with key personnel

6.6 Audit Confidentiality

The independent accountants retained by Canada shall inform Canada whether the Company has complied with its obligations under the ***License Agreement***, including without limitation whether all royalties and consideration due and payable were paid as prescribed to Canada and marketing efforts and any inaccuracies in such payments. Subject to the information contained in the foregoing audit reports, the independent accountants shall neither reveal to Canada any of the Company's internal documentation or records, nor disclose any notes or copies of the Company's records made by the independent accountants, excluding anything necessary for the report.

6.7 Duration

The auditing and verification provisions herein shall continue for 10 years following the expiry or termination of this ***License Agreement***.

6.8 No Waiver

Any royalty payment or report accepted by Canada shall not constitute a waiver by or estoppel against Canada concerning the contractual right to audit the Company, and Canada shall continue to have the right to audit as prescribed in the ***License***

Agreement.

Furthermore, an audit shall not preclude Canada from conducting subsequent audit or audits.

6.9 Discrepancy Percentage

With respect to the earned royalties (paragraph 5.2, Royalty Rate, paragraph 5.4. Sub-License Fees) in the event of any discrepancy uncovered by the audit, in excess of five percent (5.0%) of the amounts paid during the audited period, the Company shall pay forthwith to Canada both the discrepancy and the cost of the audit. Overpayments shall be credited against the next payment due by the Company to Canada.

6.10 Breach of Records Audit Article Material

The record and audit requirements are a material term of the ***License Agreement***.

7.0 REPORTS & QUALITY CONTROL

7.1 Report - Commercialization & Marketing

The Company shall, on or before the 45th day following each calendar quarter, during the term hereof and any renewal, submit to Canada written reports as to the Company's activities with respect to the exercise of ***Licensed Rights*** during the preceding twelve (12) months. Such reports shall contain:

7.1.1 a description of the steps taken by the Company to develop and ***Commercialize*** and sub-license;

7.1.2 a description of the marketing conditions for the products or processes created by the exercise of the ***Licensed Rights***; and

7.1.3 a report an the production, use and sales of the products or processes created by the exercise of the ***Licensed Rights***.

7.2 Report - Officer's Certificate

The report from the Company shall also contain a certificate from either the CEO or CFO of the Company attesting to the fact that the Company has been using commercially reasonable efforts to develop and ***Commercialize*** the products or processes created by the exercise of the ***Licensed Rights*** and that ***Commercialization*** is a material and active element of the Company's business.

7.3 Report - Audited Statement 8 Remittances

In addition to the requirements of paragraphs 7.1 (Report Contents General) and paragraph 7.2 (Report - Officer's Certificate), the report from the Company to Canada shall also contain an audited statement, which includes, without limitation:

7.3.1 an audited statement, including the amount of the products or processes created by the exercise of ***Licensed Rights*** sold by the Company and the amount of royalties or other consideration payable;

7.3.2 the names and addresses of all ***Affiliates*** and sub-licensees to whom the ***Licensed Rights*** has been sub-licensed;

7.3.3 a full account of all revenues generated by such ***Affiliates*** and sub-licenses, including the amount of products or processes created by the exercise of ***Licensed Rights*** sold;

7.3.4 a calculation of the amount due to Canada for the royalties and consideration as stipulated herein as required under paragraphs 2.5 (Sublicensing Conditions) and paragraph 2.6 (Sub-licensee Consideration); and

7.3.5 subject to paragraph 5.7 (Payment to Canada) any remittances then payable to Canada, payable to the Receiver General for Canada, of the amount of royalties or other consideration so payable.

7.4 Report— Quality Control

In addition to the foregoing, the report shall also contain internal audit results, conducted quarterly, showing the quality standards of the products or processes created by the exercise of the ***Licensed Rights*** at all production sites and at the major sale or distribution sites.

7.5 Quality Control Obligations

The Company shall comply with all quality requirements for the products or processes created by the exercise of the ***Licensed Rights*** that are prescribed by:

7.5.1 Canada from time to time in writing; and

7.5.2 any regulatory or statutory authority.

7.6 Quality Control Spot Audits by Canada

The Company shall allow Canada to conduct spot audits of the Company production and sales sites during operating hours anywhere in the ***Territory*** to ensure compliance with the prescribed quality standards.

7.7 Quality Control Spot Audits on behalf of Canada

Canada may ask the Company to conduct spot audits of the Company production and sales sites anywhere in the ***Territory*** and to disclose those results to Canada within 15 days of each audit.

7.8 Annual Report

The Company shall, on or before the 31st day of May of each calendar year, during the term hereof and any renewal, submit to Canada a copy of:

7.8.1 the Company's certified financial statements and evidence of renewal of the Company's insurance policy under section 13 of the License Agreement;
7.8.2 the Company's annual reports to shareholders; and
7.8.3 material revisions to the Company's business plan.

7.9 Annual Face-To-Face Meeting

The Company shall, on the 121st day of each calendar year, during the term of the ***License Agreement*** and any renewal, meet face-to-face with Canada to provide a progress report on the activities carried out by the Company under the ***License Agreement***.

7.10 Material terms

The reporting and quality requirements and audit rights are a material term of the ***License Agreement***.

8.0 OWNERSHIP OF TECHNOLOGY / IMPROVEMENTS

8.1 Canada Owns Licensed Rights

The Company agrees and is estopped from alleging otherwise that:

8.1.1 the ***Licensed Rights*** are vested in and are the sole property of Canada;

8.1.2 ownership and all rights related to, connected with, or arising out of the foregoing, including, without limitation:

8.1.2.2 ***Patents, Intellectual Property, Confidential Information***, copyright, the right to produce, publish or cause to be produced, and all published information material and documents;

8.1.2.3 the right to issue a license;

are vested in and are the sole property of Canada, and

8.1.3 the Company shall have no rights to the foregoing except as may be expressly granted under this ***License Agreement***, and the Company shall not apply for any proprietary or other right and shall not divulge or disclose, without the prior written consent of Canada, any information, material or documents concerning the foregoing or make available in any way or use the ***Licensed Rights***, except as expressly provided in the ***License Agreement***.

8.2 No Impeachment

The Company shall neither impeach, contest or otherwise attack, directly or indirectly, the validity, enforceability or ownership of the ***Patents*** or any ***Intellectual Property*** rights held by Canada, or Canada's right, title and interest in and to the ***Licensed Rights*** nor assist, counsel or procure any third party to do the same.

8.3 Inimical Use of Confidential Information

The Company shall not use any ***Confidential Information*** obtained from Canada in the negotiation of the ***License Agreement***, under due diligence searches or otherwise related to this ***License Agreement***, in any manner that either violates the Company's rights and obligations under the ***License Agreement*** or is inimical to the interests of Canada.

8.4 Improvements - Ownership

Unless expressly agreed to otherwise in writing by the ***Parties***, the ownership of any ***Improvement*** made by or on behalf of a ***Party*** shall immediately, after creation, vest exclusively in that ***Party***.

8.5 Company Improvements - Disclosure

The Company shall disclose to Canada forthwith all ***Improvements***, innovations and

discoveries developed or created by or on behalf of the Company, solely or jointly with others (including ***Affiliates*** and sub-licensees), which related to the ***Licensed Rights***, together with any ***Intellectual Property*** rights residing therein.

8.6 Company Improvements — License to Canada

The Company hereby grants to Canada a personal, non-transferable, non-exclusive, worldwide, perpetual, irrevocable, royalty-free and fully paid-up license for the ***Improvements*** (including data and reports related thereto), made by or on behalf of the Company under paragraph 8.4 (Improvements — Ownership) and disclosed to Canada under paragraph 8.5 (improvements — Disclosure) for the purposes set out in paragraph 2.2 (Carve Out). Further, Canada may sub-license such ***Improvements*** for the purposes of carrying out the purposes set out in paragraph 2.2 (Carve Out).

Termination of the ***License Agreement*** shall not terminate the foregoing license to Canada or any subsisting sub-licenses.

9.0 DISCLAIMERS

9.1 Estoppel Statement/Disclaimer of Express / Implied Warranties

The Company acknowledges that there is some question as to the integrity of ownership of the ***Licensed Rights*** and ***Patents*** and the Company accepts those risks.

The ***Licensed Rights*** and ***Patents*** are provided to the Company on an "as is" basis. Canada makes no warranties, representations or conditions, express or implied, of any nature, and Canada disclaims all warranties, representations or conditions, for the ***Licensed Rights***, the ***Patents***, the ***Intellectual Property*** or the ***Confidential Information*** including, without limitation:

9.1.1 merchantability;
9.1.2 quality (either as discussed or with respect to a sample / model);
9.1.3 fitness for any or a particular purpose:
9.1.4 commercial utility or practical purpose;
9.1.5 susceptibility of yielding valuable results or results are free of defects or otherwise harmless;
9.1.6 latent or other defects;
9.1.7 infringement or non-infringement of Patents or other third party rights;
9.1.8 conformity with the laws of any jurisdictions; or
9.1.9 fitness for the Company's corporate objectives (whether or not expressly or implicitly communicated to Canada).

For greater certainty, no information or advice given by Canada shall create a warranty or representation or condition other than as expressly stated in the ***License Agreement***. The Company hereby accepts the ***Licensed Rights*** and the ***Patents*** "as is", with all faults, and the entire risk as to satisfactory quality, performance, accuracy and effort is with the Company. In no event shall Canada be liable for any direct, indirect, incidental, special, exemplary, or consequential damages (including, but not limited to, procurement of substitute goods or services, loss of use, data or profits, or business interruption) however caused and on any theory of liability, whether in contract, strict liability, or tort (including negligence or otherwise) arising in any way out of the exercise of the ***Licensed Rights*** by the Company, its ***Affiliates*** or sub-licensees, even if advised of the possibility of such damage.

9.2 Disclaimer of Statutorily Implied Warranties

No legal or equitable warranties or conditions implied by law or convention under any domestic, foreign or international legal regime, or from a course of dealing or usage of trade, shall apply to the ***License Agreement***. The Company acknowledges this disclaimer and is estopped from relying on any such representations, warranties or conditions against Canada.

9.3 Confidential Information Without Warranty / No Reliance

The Company shall not rely in any way on the quality, accuracy or completeness of any ***Confidential Information*** provided by Canada under the ***License Agreement***. Any use of such ***Confidential Information*** shall be at the Company's sole risk and expense. Any ***Confidential Information*** provided to the Company by Canada is without any warranty or guarantee or representation or warranty of any kind whatsoever other than as expressly provided herein.

9.4 No Liability to Canada from Exercise of Rights

The Company undertakes to use the ***Licensed Rights*** and apply ***Confidential Information*** of Canada entirely at its own risk and under its own responsibility, and that the Company will have no recourse against Canada with respect to any consequences of such application.

9.5 Third Party Representations

The Company shall not represent to any ***Affiliate*** or sub-licensee the existence of any warranty or condition concerning the ***Licensed Rights***.

9.6 Disclosure & Due Diligence

The Company acknowledges that:

9.6.1 Canada has made full and frank disclosure of all facts the Company deemed relevant before executing the ***License Agreement***;

9.6.2 The Company has conducted a due diligence search of all matters relevant to the ***Licensed Rights***, the ***Patents*** and the ***License Agreement***;

9.6.3 Canada has made all best efforts to identify the significant characteristics of the ***Licensed Rights*** and that Canada makes no representation that all the characteristics both favorable and unfavorable have been identified; and

9.6.4 Canada is either under no duty to warn the Company or the Company unconditionally waives any such duty, about the ***Licensed Rights*** or ***Commercialization***.

10.0 PATENT PROTECTION & REGULATORY REQUIREMENTS

10.1 Patent Costs

The Company shall pay all costs related to and maintaining ***Patents*** (and shall reimburse Canada for any of these costs that Canada may pay during any term of the ***License Agreement***), as they are incurred, and within thirty (30) days of being invoiced for such costs.

10.2 Right to Patent

Nothing in the ***License Agreement*** shall limit or restrict Canada from seeking to patent ***Improvements*** made by Canada.

10.3 The Company Shall Obtain Regulatory Permissions

The Company shall use commercially reasonable efforts to obtain any authorizations, permits, certificates or other regulatory permissions which may be required in order for the Company to legally carry out all of its activities under the ***License Agreement***,

including but not limited to ***Commercialization***, at the Company’s sole cost and expense without right of set-off.

10.4 Her Majesty Not Obligated

Nothing in the ***License Agreement*** shall obligate any emanation of Her Majesty the Queen in Right of Canada to grant any required authorizations, permits, certificates or other regulatory permissions. Conversely, there is no implication by the execution of the ***License Agreement*** that the Company will be granted any required authorization, permits, certificates or other regulatory permissions necessary for the effective Commercialization of the ***Licensed Rights***.

11.0 CONFIDENTIALITY / FIDUCIARY OBLIGATIONS & EQUITABLE REMEDIES

11.1 Existing Confidentiality Agreements

The ***Confidentiality Agreements*** entered into by the Parties on May 1, 2007, and November 1, 2008, respectively, shall end on the ***Execution Date*** of the ***License Agreement***. However, all rights and obligations of the Parties under the ***Confidentiality Agreements*** that expressly or by their nature survive termination of those agreements shall continue in full force and effect until they expressly or by their nature expire.

11.2 Confidentiality Obligations

Commencing on the ***Execution Date*** of this ***License Agreement***, ***Confidential Information*** disclosed by one ***Party*** to the other ***Party*** under this ***License Agreement*** shall be:

11.2.1 held in confidence and in trust by the receiving ***Party***;

11.2.2 used by the receiving ***Party*** exclusively for the purposes authorized under the ***License Agreement*** and for no other purpose whatsoever;

11.2.3 safeguarded by the receiving ***Party*** using all reasonable measures and taking such action as may be appropriate to prevent the unauthorized access, use or disclosure of the ***Confidential Information***; and

11.2.4 not disclosed to third parties without the prior written consent of the disclosing ***Party***.

11.3 No Waiver of Privilege

Each ***Party*** acknowledges that the ***Confidential Information*** of the disclosing ***Party*** is the property of the disclosing ***Party*** or a third party and that none of the latter

intend to or do waive any rights, title or privilege they may have in respect of any of the ***Confidential Information***.

11.4 Common Law Duty of Confidentiality

Nothing in this ***License Agreement*** derogates, displaces or otherwise diminishes the common law or equitable duty of confidentiality vested in the receiving ***Party*** concerning the ***Confidential Information***.

11.5 Confidentiality Exclusions

Article 11.2 (Confidentiality Obligations) does not apply to information which, even if it may be marked "confidential', is not really confidential, in that

11.5.1 IN PUBLIC DOMAIN - the information was legally and legitimately in the public domain through no act or omission of the receiving ***Party*** at the time of disclosure by the receiving ***Party***;

11.5.2 PUBLISHED - the information was legally and legitimately published or otherwise becomes part of the public domain through no act or omission of the receiving ***Party*** at the time of disclosure by the receiving ***Party***;

11.5.3 ALREADY KNOWN TO THE RECEIVING PARTY - the information was already in the possession of the receiving ***Party*** at the time of disclosure and was not acquired by the receiving ***Party***, directly or indirectly, from the disclosing ***Party*** (as shown by documentation sufficient to establish the timing of such possession), and the receiving ***Party*** is free to disclose the information to others without breaching any contractual or trust obligations or common law duties;

11.5.4 THIRD PARTY DISCLOSES - the information becomes available from an outside source who has a lawful and legitimate right to disclose the information to others, and the receiving ***Party*** is free to disclose the information to others without breaching any contractual or trust obligations or common law duties;

11.5.5 INDEPENDENTLY DEVELOPED - the information was independently developed by the receiving ***Party*** without any of the ***Confidential Information*** being reviewed or accessed by the receiving ***Party*** (as shown by documentation sufficient to establish the timing of such development); or

11.5.6 JUDICIAL/ADMINISTRATIVE ORDER - the information was released due to a compulsory order under a judicial process or under a compulsory regulatory (including securities) requirement, none of which was invited by, or consented to, by the receiving ***Party*** and the receiving ***Party*** made all reasonable efforts to secure a court order to limit production, use and disclosure of the information to the narrowest class practical under the circumstances.

11.6 Secure Location

Each ***Party*** shall keep the ***Confidential Information*** of the other ***Party*** in a secure location accessible only to its employees specifically authorized to have access pursuant to this ***License Agreement***. Each ***Party*** shall ensure that its employees complies with the terms and conditions of this ***License Agreement*** and shall enter into agreements with such employees if necessary to give effect to this obligation.

11.7 Return of Confidential Information

If this ***License Agreement*** expires or is terminated, the ***Parties*** shall return to each other the ***Confidential Information*** disclosed to them under this ***License Agreement*** and any notes, reports and other materials prepared by the receiving ***Party*** from the disclosing ***Party***'s ***Confidential Information*** except that Canada shall be entitled to retain one copy of such records for the purposes of meeting Canada's obligations under the federal laws of Canada and for the purposes of paragraph 8.6 (Improvements - License to Canada).

11.8 Confidential Information is Proprietary

The ***Confidential Information*** of each ***Party*** is and shall remain the exclusive property of that ***Party*** or third parties and the receiving ***Party*** shall not claim any rights, title, interest or ownership in the ***Confidential Information***. The receiving ***Party*** shall not contest any such rights, title, interest or ownership.

11.9 Legal and Equitable Remedies

Should a ***Party*** commit or threaten to commit a serious or material breach of its confidentiality or fiduciary obligations under this Article 11, then the other ***Party*** may pursue any and alt legal and equitable remedies, including without limitation, injunctive relief, accounting for profits, redistribution, damages, constructive trust and disgorgement. Disgorgement means, for the purposes of the ***License Agreement***, the ejection of all benefits gained by the receiving ***Party***, traceable to the material breach, notwithstanding that such disgorgement

may exceed the damages directly suffered by the disclosing ***Party*** or deprivation suffered by the disclosing ***Party*** for such breach.

11.10 No Hiring of Canada's Employees

The Company shall not:

11.10.1 solicit, hire, retain or secure;

11.10.2 directly or indirectly, including without limitation, the use of consultants, ***Affiliates*** or third parties;

11.10.3 any of the agents, servants or employees of Canada;

11.10.4 which agents, servants or employees are employed or retained in connection with, or whose responsibilities relate in whole or in part, to the ***Confidential Information***, the ***Licensed Rights*** or the ***Patents***; or helped produce or create the ***Confidential Information***, the ***Licensed Rights*** or the ***Patents***;

to accept employment with the Company of any of its ***Affiliates***, unless

11.10.5 Canada grants in advance its written permission to such a solicitation or the employment of such a person; or

11.10.6 [*] have elapsed from the ***Execution Date***.

11.11 Exemption

The prohibition in paragraph 11.10 (No Hiring of Canada's Employees) does not apply to general solicitations of employment issued by the Company and any hiring resulting from such solicitations that are:

11.11.1 not directed towards the employees of Canada; and

11.11.2 do not involve the ***Confidential Information***, the ***Licensed Rights*** or the ***Patents***.

11.12 Contact Only Under License Agreement

The ***Parties*** shall have no discussions, correspondence or other contact with the other ***Party***, its licensees, confidants or any person concerning the ***License Agreement***, except through the designated representative of the other ***Party*** or

any delegates identified in writing by the designated representative from time to time.

11.13 Terms Of Agreement Confidential But Not Existence of Agreement

The ***Parties*** agree that terms of this ***License Agreement*** are confidential but not its existence. The terms of this ***License Agreement*** shall not be disclosed by a Party unless disclosure is required by law or if the other ***Party*** agrees to the disclosure in writing prior to disclosure.

12.0 CORPORATE REPRESENTATIONS & WARRANTIES

12.1 The Company Incorporated & Authorized 8 Bound

The Company represents and warrants to Canada that as of the ***Execution Date***:

12.1.1 ABILITY

it can ***Commercialize***, and the Company has or will have the necessary access to funds, resources, knowledge, facilities and personnel to perform its obligations under the ***License Agreement***, including to use commercially reasonable efforts to ***Commercialize***;

12.1.2 AUTHORIZATION

it is authorized and has the corporate power and authority to negotiate, execute, comply with and satisfy its obligations, without qualification, under the ***License Agreement***;

12.1.3 INCORPORATION JURISDICTION

it has been duly incorporated and organized under the laws of the state of Delaware and is validly existing under the laws of Iowa;

12.1.4 EXTRA-PROVINCIAL REGISTRATION

it is duly qualified, licensed or registered to carry on business in the Province or State of Delaware.

12.1.5 ENFORCEABLE

it is bound by the ***License Agreement***, upon execution, and the ***License Agreement*** constitutes a legal, valid and binding obligation on the Company,

enforceable against the Company in accordance with the terms of the ***License Agreement***, except as those terms may be limited by applicable bankruptcy laws and general principles of equity,

12.1.6 LITIGATION

it has no knowledge of any legal proceeding or order pending against or, to the knowledge of the Company, threatened against or affecting, the Company or any of its properties or otherwise that could adversely affect or restrict the ability of the Company to consummate fully the transactions contemplated by this ***License Agreement*** (including without limitation the ***Commercialization***) or that in any manner draws into question the validity of this ***License Agreement***;

12.1.7 VERACITY OF STATEMENTS

no representation or warranty by the Company contained in this ***License Agreement*** and no statement contained in any certificate, schedule or other instrument furnished to Canada pursuant hereto or in connection with the transactions contemplated hereby, contains any untrue statement of a material fact or omits to state a material fact;

12.1.8 INCONSISTENT AGREEMENTS / OBLIGATIONS

it has not given any understanding, express or implied, to any third party which would:

12.1.8.2 preclude the Company from fulfilling its obligations under the ***License Agreement***; or

12.1.8.3 cause the Company to breach an agreement with a third party;

12.1.9 NO MARCH IN RIGHTS

it is not subject any “march in” or third party rights, (contractual or statutory, contingent or vested) which would give that third party any rights to the ***Licensed Rights*** not otherwise explicitly described in the ***License Agreement***; and

12.1.10 NO BREACH OF THIRD PARTY AGREEMENTS

its execution of the ***License Agreement*** does not contravene its constituent documents or any law, regulation or official directive or any of its obligations or undertakings by which it or any of its assets are bound or cause a limitation

on its powers or the powers of its directors to be exceeded.

12.2 Canada Authorized

Canada represents and warrants to the Company as of the ***Execution Date***:

12.2.1 AUTHORIZATION

Canada has the power and authority to negotiate, execute and comply with the ***License Agreement***, subject to all applicable laws and the royal prerogative; and

12.2.1.2 no further action is required by or in respect of any governmental or regulatory authority; and

12.2.1.3 the ***License Agreement*** is legal, binding and enforceable in accordance with its terms.

13.0 INDEMNITY, INSURANCE AND LIABILITY ALLOCATION & CAPS

13.1 The Company's Indemnification

The Company shall;

13.1.1 indemnify; and

13.1.2 save harmless;

Canada (and her employees, servants and agents),

13.1.3 from and against all claims, demands, losses, penalties, damages, costs, (including reasonable solicitor and own-client costs and expert witness costs), actions, suits or other proceedings whatsoever, whether groundless or otherwise,

13.1.4 brought or prosecuted in any manner which heretofore or hereafter may be made by a third party against Canada or her employees, servants and agents;

13.1.5 however and whenever arising out of, relating to, occasioned by or attributed to,

a) any acts or conduct (including, without limitation, omissions, misrepresentations, errors and offences) of the Company, its employees, servants, agents, advisors, sub-licensees or ***Affiliates*** (whether by reason of negligence or otherwise) in the performance by the Company of the provisions of the ***License Agreement*** or any activity undertaken or purported to be undertaken under the authority or pursuant to the terms of the ***License Agreement***, including without limitation, exercise of the ***Licensed Rights*** and ***Commercialization***;

b) any infringement or alleged infringement by the ***Patents***, the ***Licensed Rights*** or ***Licensed Products*** of proprietary rights of any including, without limitation, patent, trade-mark, copyright or trade secret rights;

c) any claim the ***Patents***, the ***Licensed Rights*** or the ***Licensed Products*** or any aspect or use thereof by the Company infringes or constitutes misappropriation of the intellectual property rights of any third party; and

d) any claim or demand against the ***Patents***, the ***Licensed Rights***, the ***Licensed Products*** or the interest of Canada or the Company therein.

Further, the Company shall not third party Canada for any such claims, actions, suits or other proceedings taken solely against the Company and the Company hereby expressly waives any rights it has against Canada for claims of infringement.

13.2 Indemnity Separate / Continuing

The foregoing indemnity is a continuing obligation, separate and independent from the other obligations of the Company and survives termination of, expiration of, or the acceptance of repudiation of the ***License Agreement***. It is not necessary for Canada to incur expense or make payment before enforcing a right of indemnity conferred hereunder.

13.3 Insurance

The Company shall ensure that both the Company and each of its ***Affiliates*** and sub-licensees shall obtain and maintain, throughout the term of the ***License Agreement*** (and any renewal thereof) or duration of the sub-licenses (as the case may be), comprehensive general liability insurance for any and all claims, actions, liabilities and expenses resulting from the ***Commercialization*** of the ***License Rights***.

13.3.1 INSURANCE COMPANY

The insurance policy shall be obtained from a qualified insurance company licensed to do business in the applicable jurisdictions.

13.3.2 NAMED INSURED

The insurance policy shall name Her Majesty the Queen in Right of Canada and Her employees, servants and agents as "additional insureds".

13.3.3 LIMITS

As of the ***Execution Date***, the insurance policy shall include commercial general liability insurance, and shall have monetary limits in the amount not less than one million dollars ($1,000,000) for each single occurrence or claim. Following the submission of an Investigational New Drug covering a Licensed Product and prior to the beginning of a Phase 1 Clinical Study, the insurance policy shall include commercial general liability insurance, that includes products liability insurance, and shall have monetary limits in an amount not less than five million dollars ($5,000,000) for each single occurrence or claim. The minimum amount of insurance coverage required under this ***License Agreement*** shall not be construed as a limit of liability.

13.3.4 TERMINATION NOTICE

The insurance policy shall provide for thirty (30) business days written notice by the insurer to the Company and Canada by registered or certified mail in the event of any modification, cancellation or termination of the insurance policy.

13.3.5 COPY

The Company shall provide Canada a copy of the insurance policy not later than 30 days alter execution of the ***License Agreement***, and thereafter upon the written request of Canada. This obligation shall apply each time the monetary limits are increased pursuant to clause 13.3.3, in which case the copy shall be provided not later than 30 days after the monetary limits in the insurance policy are increased. This obligation shall survive termination or expiration of the ***License Agreement***.

13.3.6 INSURANCE UNAVAILABLE

If insurance required to meet the monetary limits in clause 13.3.3 is unavailable, the

Parties shall review the situation, and Canada may elect to either allow the Company to obtain the insurance that is available, or alternatively terminate the ***License Agreement***.

13.4 Canada's Liability Cap

Canada's liability for:

13.4.1 breach of the representations, conditions or warranties contained herein or any of the other provisions of the ***License Agreement*** or any other breach giving rise to liability, including a breach of a condition or fundamental term or fundamental breach or breaches; or

13.4.2 in any other way arising out of or related to the ***License Agreement***; or

13.4.3 for any cause of action whatsoever and regardless of the form of action (including breach of contract, trust, strict liability, tort [*], or any other legal or equitable theory);

shall be limited to the Company's actual direct (immediate and foreseeable at the time of negotiation to both ***Parties***), provable damages in an amount not to exceed in the aggregate a sum equal to or less than the net consideration received by Canada from the Company under paragraph 5.2 (Royalty Percentage Rate) for the time period commencing from the ***Execution Date*** up to and including the date of judicial judgment or arbitrator's decision.

13.5 Excluded Heads of Damage

Canada shall not be liable to the Company, its employees, servants, agents, successors, assigns, ***Affiliates*** or sub-licensees for damages in respect of:

13.5.1 incidental, indirect, special, punitive, exemplary damages;

13.5.2 any economic loss, consequential damages, relational loss, including but not limited to lost business revenue, lost profits, business interruption, failure to realize expected savings, loss of data, loss of business opportunity suffered by the Company or any claim whatsoever and whenever made against the Company by any other party;

(whether grounded in tort [*], strict liability, contract, trust or otherwise,) even if:

13.5.3 Canada was advised of the possibility of such damages, or

13.5.4 the damages encompassed by subparagraphs 13.5.1 and 13.5.2 were foreseeable by Canada, or

13.5.5 such damages resulted from a fundamental breach of the ***License Agreement***.

Further, Canada shall have no duty to warn the Company for matters arising directly or indirectly under the ***License Agreement***.

13.6 No Actions Against Employees

The Company acknowledges and estopped from and waives any rights the Company might have to commence and prosecute any action whatsoever, regardless of form or grounds (including without limitation negligence, misrepresentation, fiduciary, deceit) against any of Canada's employees, servants, agents or officers, arising out of any

13.6.1 claimed breach of the ***License Agreement***;
13.6.2 transactions under the ***License Agreement***;
13.6.3 negotiations leading to the ***License Agreement***; or
13.6.4 in any other way related to the ***License Agreement***.

For greater clarity, the Company's remedies for any breach of or ***Dispute*** under the ***License Agreement***, lies only against Canada, and only within the aforementioned parameters prescribed by the ***License Agreement***.

13.7 Notifications

Canada shall notify the Company of any claim that falls within the parameters of the respective indemnification obligations as soon as practical. In any case such notice shall be made forthwith upon notice that a claim may be prosecuted or a cause of action exists.

14.0 INFRINGEMENT

14.1 Third Party Suit

Subject to Article 13 (Indemnification), in the event of any threatened or actual suit against the Company in consequence of the exercise of any rights and licenses granted herein, the Company shall; promptly inform Canada and the ***Parties*** will Jointly decide on the steps to be taken in the circumstances. In any event, the Company will always have the sole right to defend itself as it determines against any suit or other action brought against the Company or its employees or agents.

14.2 Infringement Uncovered

Each ***Party*** will notify the other promptly in writing when any infringement of the ***Licensed Rights*** or ***Patents*** is uncovered or suspected.

14.3 Company May Sue

The Company shall have the right to enforce the ***Patents*** against any infringement or alleged infringement thereof, and shall at all times keep Canada informed as to the status thereof. Subject to Canada's prior written approval (which will not be unreasonably withheld), the Company may, at its own expense, institute suit against any such infringer or alleged infringer and prosecute such suit in a manner consistent with the terms and provisions hereof. Canada shall reasonably cooperate in any such litigation at the Company's expense, and the Company shall keep Canada apprised in a timely manner of all litigation activities. In any litigation under this article, the Company shall not have the right to settle or otherwise compromise Canada's position as a licensor or owner of the ***Patents*** without Canada's prior written consent.

14.4 Distribution of Company's Recovery

In the event of a recovery by the Company of punitive and non-punitive damages (net of legal fees and out of pocket costs of the action) under paragraph 14.3 for royalty-bearing products, the Company shall pay to Canada [*] of such recovery.

14.5 Canada May Sue

If the Company elects not to enforce the ***Patents*** as to any infringement or alleged infringement thereof, then the Company shall so notify Canada in writing within one (1) month of receiving notice that an infringement exists, and Canada may, in its sole judgment and at its own expense, take steps to enforce the ***Patents*** against such infringement or alleged infringement and control, settle, and defend such suit in a manner consistent with the terms and provisions hereof, and recover for its own account any damages, awards or settlements resulting therefrom.

15.0 TERMINATION

15.1 By Canada for Cause

The ***License Agreement***, at the option of Canada, without prejudice to any other rights in law of equity held by Canada (including any right of indemnity), may be

terminated forthwith by Canada without compensation to the Company if:

15.1.1 INSUFFICIENT EFFORTS

The Company fails to use its commercially reasonable efforts to develop or ***Commercialize*** and does not cure such failure within ninety (90) days of written notice from Canada;

15.1.2 NO PAYMENT

The Company fails to make any payment owed to Canada under the ***License Agreement*** and does not make such payment within sixty (60) days of the due date;

15.1.3 BREACH OF CONFIDENTIALITY

The Company uses or discloses ***Confidential Information*** of Canada in a manner inconsistent with its obligations under the ***License Agreement*** or fails to safeguard the ***Confidential Information*** of Canada;

15.1.4 BREACH OF BUSINESS PLAN

The Company fails, neglects, refuses or is unable to comply with the business plan created and submitted under paragraph 4.1 (Business Plan);

15.1.5 QUALITY CONTROL & AUDIT

The Company refuses, neglects or fails to meet quality standards or allow access for quality audit purposes contrary to paragraph 7.0 (Reports & Quality Control) or provide or allow the audit of the reports and records as required under Article 6.0 (Records and Audit);

15.1.6 CEASES BUSINESS

The Company ceases to actively carry on business;

15.1.7 MULTIPLE BREACHES

The Company breaches three or more provisions of the ***License Agreement*** within any consecutive twelve (12) month period, notwithstanding that such breaches were subsequently cured;

15.1.8 CROSS-DEFAULT

The Company breached a provision of another agreement with Canada that was executed with the Public Health Agency of Canada, and that breach occurred during the term of the ***License Agreement***;

15.1.9 CRIMINAL CONVICTION

The Company was convicted of a criminal or regulatory offence, the nature of which directly or indirectly affects the ability of the Company to conduct itself hereunder or to ***Commercialize*** in an effective and timely manner, or otherwise prejudices ***Commercialization***;

15.1.10 GENERAL BREACH

The Company commits or permits a breach of any of the other terms and conditions herein contained and does not remedy such breach within sixty (60) days after being required in writing to do so by Canada;

15.1.11 REPUDIATES

The Company expressly or implicitly repudiates the ***License Agreement*** by refusing or threatening to refuse to comply with any of the provisions of the ***License Agreement***.

15.2 Automatic Termination

The ***License Agreement*** and all rights granted to the Company pursuant to the ***License Agreement*** shall immediately terminate and revert to Canada by operation of contract, without prejudice to any other rights in law of equity held by Canada (including any right of indemnity) and without compensation to the Company, effective the business day prior to the applicable triggering event, namely if:

15.2.1 ASSIGNMENT

The Company assigns the ***License Agreement*** without the prior written consent of Canada, contrary to the provisions of paragraph 18.2 (No Assignment Without Consent); or

15.2.2 BANKRUPTCY

The Company becomes bankrupt or insolvent or otherwise

15.2.2.2 has a receiving or winding up order made or sought against it;

15.2.2.3 has a meeting proposed or convened, seeking or actually passing a resolution to appoint a trustee or official manager;

15.2.2.4 has a receiver, receiver-manager, liquidator, trustee in bankruptcy, custodian or any other officer with similar powers appointed for the Company or such an order is sought;

15.2.2.5 has any or all of its assets seized or otherwise attached for the benefit of creditors;

15.2.2.6 proposes or convenes a meeting to seek or passes a resolution for winding up;

15.2.2.7 takes the benefit of any statute, at the time in force, relating to bankrupt or insolvent debtors for the orderly payment of debts;

15.2.2.8 makes a general assignment for the benefit of creditors;

15.2.2.9 submits a proposal or arrangement under any debtor/creditor legislation;

15.2.2.10 is the subject of a petition or files an assignment under the Bankruptcy Act or any successor legislation; or

15.2.2.11 does or attempts anything analogous to the aforementioned events or having a substantially similar effect to any of the aforementioned events under the laws of any jurisdiction.

15.3 Termination Not A Penalty

The Company acknowledges, and is estopped from alleging otherwise, that the termination provisions in paragraph 15.2 do not constitute a penalty, and are otherwise fair, just and proportional given:

15.3.1 the nature of the ***Parties***;
15.3.2 their respective mandates and corporate objectives;
15.3.3 the allocation of risks under the ***License Agreement***;
15.3.4 the goals of the ***Parties***;
15.3.5 nature of the ***Licensed Rights***; and
15.3.6 the consequences to Canada if the Company commits the aforementioned breaches.

15.4 Procedure

Termination shall be implemented by a notice effective as of the date slated therein, but termination shall be subject to paragraph 15.6 (The Company's Duties on Termination) and be without prejudice:

15.4.1 to the right of Canada to sue for and recover any royalties or other sums due Canada; and

15.4.2 to the remedy of either ***Party*** in respect of any previous breach of the ***License Agreement***.

15.5 Effect on Sub-licensees

All sub-licenses, including those granted to ***Affiliates***, shall terminate with the ***License Agreement***.

15.6 The Company's Duties on Termination or Expiration

A) Upon termination of the ***License Agreement*** by Canada, the Company shall at its own cost:

15.6.1 return immediately to Canada all ***Licensed Rights*** and ***Confidential Information*** of Canada, including copies thereof;

15.6.2 certify in writing to Canada within thirty (30) days of termination, that to the best of the Company's knowledge, all of the ***Confidential Information*** (including copies) of Canada has been returned;

15.6.3 deliver a detailed statement to Canada of the inventory of the products made from the exercise of the ***Licensed Rights*** then existing, but not sold by the Company, as of the date of expiration or termination;

15.6.4 provide Canada the right of first refusal to purchase from the Company any products made from the exercise of the ***Licensed Rights*** inventory at fair market value;

15.6.5 dispose of any remaining products made from the exercise of the ***Licensed Rights*** in inventory as specified by Canada subject always to any obligations under Article 5.0 (Fees & Royalties);

15.6.6 pay all costs due under the ***License Agreement*** either by the Company on its behalf or a sub-licensee, up to and including the termination date, within thirty (30) days of the termination;

15.6.7 pay all costs due under the ***License Agreement***, subsequent to the termination, for any products made from the exercise of the ***Licensed Rights*** sold after termination, within thirty (30) days of the liability being incurred;

15.6.8 grant back to Canada any technology rights, clinical or research data arising from the ***Licensed Rights*** or otherwise under the ***License Agreement***;

15.6.9 assign to Canada (or her nominee) any equities, goodwill, or other rights which the Company has or alleges to have acquired in the ***Licensed Rights*** or derived in the ***Commercialization***. The Company shall also execute such further documentation as Canada may reasonably request m order to confirm such assignment;

15.6.10 pay immediately to Canada any royalties, fees, reimbursements or other financial obligations irrespective of the fact such monies are owed, but for the termination or expiration, not yet payable; and

15.6.11 assign or transfer for [*] in total consideration, any and all authorizations, permits, certificates or other regulatory permissions obtained in order to Commercialize, to any third party identified by Canada or to Canada itself, within ninety (90) days of termination or expiration, unless otherwise requested by Canada.

B) Upon expiration of the ***License Agreement***, the Company shall at its own cost, perform the duties set out in sections 15.6.1 to 15.6.8. Further, upon expiration, the Company shall grant to Canada the right to exercise an option to negotiate with the Company an agreement dealing with the matters set out in sections 15.6.9 to 15.6.11. The ***Parties*** shall negotiate the agreement in good faith. The agreement shall contain mutually acceptable terms and conditions and the consideration shall be commercially reasonable.

15.7 Surviving Obligations

All obligations of the ***Parties*** which expressly or by their nature survive termination or expiration, shall continue in full force and effect subsequent to and

notwithstanding such termination or expiration, until they are satisfied or by their nature expire. For greater clarity, and without restricting the generality of the foregoing, the following provisions survive termination or expiration:

15.7.1 Paragraph 2.2 (Carve Out);
15.7.2 Article 5.0 (Fees and Royalties);
15.7.3 Article 6.0 (Records & Audit);
15.7.4 Paragraphs 8.4 to 8.6 (Improvements — Ownership, Company Improvements - Disclosure, Company Improvements - License to Canada);
15.7.5 Article 11.0 (Confidentiality / Fiduciary & Equitable Remedies);
15.7.6 Article 13.0 (Indemnity, Insurance and Liability Allocation & Caps); and
15.7.7 Paragraph 15.6 (The Company's Duties on Termination or Expiration).

16.0 ALTERNATE DISPUTE RESOLUTION (ADR)

16.1 Negotiations

16.1.1 INFORMAL NEGOTIATIONS

If a ***Dispute*** arises between the ***Parties***, then: within 6 months from when the allegedly aggrieved ***Party*** knows or should know of the ***Dispute***, the contact individuals in Article 20.1 (Notice) shall,

16.1.1.2 contact their counterpart, and attempt bona fide efforts to diligently resolve the ***Dispute*** through amicable negotiations:

16.1.1.3 provide full, frank and timely disclosure of all relevant facts, information and documents to facilitate those negotiations;

16.1.1.4 resolve the ***Dispute*** within 7 days;

16.1.1.5 reduce the ***Dispute*** to writing, and if the contact persons cannot agree on the wording of the ***Dispute***, both contact persons shall submit to each other their written version of the ***Dispute***.

16.1.2 FORMAL NEGOTIATIONS

If the ***Parties*** are unable to resolve the ***Dispute*** within fourteen (14) calendar days from the receipt by the other ***Party*** of the written version of the ***Dispute***, then within the following thirty (30) days the Dispute shall be referred to the Chief Public Health Officer, on behalf of Canada, and to the CEO, on behalf of the Company (or their directly reporting designates), to negotiate a resolution.

16.1.2.2 These individuals may not delegate, substitute or direct, surrogates for them at these negotiations.

16.1.2.3 These individuals shall meet in person to negotiate and the ***Parties*** shall bear their own costs.

16.1.2.4 Unless otherwise agreed, the meetings shall alternate between Company, HQ, and Canada, HO, commencing in Ottawa, Ontario, for the first meeting for the first ***Dispute***. There shall be one meeting only per ***Dispute***, which meeting shall not exceed one (1) business day in length.

16.1.2.5 The ***Parties*** may bring no more than two consultants to a meeting. The two consultants shall not have a right of audience or otherwise to negotiate the ***Dispute***.

16.2 Mediation

If, within thirty (30) days following the close of the meeting under paragraph 16.1.2 (Formal Negotiations), the ***Parties*** have not succeeded in negotiating a resolution, then the ***Parties*** shall jointly submit the ***Dispute*** to mediation.

16.3 Skip Mediation - Direct to Arbitration

If the ***Parties*** cannot agree to jointly submit the ***Dispute*** to mediation, then either ***Party*** may submit the ***Dispute*** to binding arbitration.

16.4 Mediation Process

The Parties shall

16.4.1 APPOINTMENT OF MEDIATOR

appoint a mutually acceptable mediator with sixty (60) days from the close of the formal negotiation meeting under sub-paragraph 16.1.2 (Formal Negotiations);

16.4.2 GOOD FAITH EFFORTS

participate in good faith in the mediation and negotiations related thereto;

16.4.3 EMPOWERED REPRESENTATIVES

representatives sent to the mediation shall be empowered or have sufficient delegated authority to resolve, compromise, negotiate or settle the ***Dispute*** submitted to mediation, without seeking further instructions or approvals from any superiors or committees / corporate structures, unless the nature, seriousness or financial quantum of the ***Dispute*** by law or corporate policies or practices requires approval from the respective corporate or government structure. In such event, such approval shall be obtained within five (5) business days of the proffer of any settlement offer;

16.4.4 COSTS

bear the costs of the mediation equally, except that each ***Party*** shall bear its own personal costs of the mediation;

16.4.5 FULL DISCLOSURE

a full, frank and timely manner all relevant facts, information and documents to facilitate the mediation; and

16.4.6 LOCATION

The mediation shall take place in the city that was not the site of the formal negotiations for the ***Dispute***.

16.5 Unsuccessful Mediation —Remit to Arbitration

The ***Dispute*** shall be referred to binding arbitration by either or both ***Parties*** if the ***Parties*** are not successful in resolving the ***Dispute*** through mediation.

16.6 Arbitration - Structure

After negotiation and if applicable, mediation), any subsisting ***Dispute*** between the ***Parties***, shall be referred to arbitration by a written submission signed by either Canada or the Company.

16.6.1 FORUM LAWS PROCEDURAL RULES

The arbitration tribunal shall be governed by the UN Commercial Arbitration Code, referred to in the Commercial Arbitration Act, R.S.C. 1985, c.C-34.6 (“Code”).

16.6.2 NUMBER OF ARBITRATORS

The arbitration tribunal shall consist of one arbitrator chosen by the ***Parties***.

16.6.3 ISSUE BEFORE ARBITRATOR

The scope of the arbitration shall be limited to the resolution of the ***Dispute*** submitted to arbitration.

16.6.4 APPLICABLE SUBSTANTIVE LAW

The arbitration tribunal shall decide the ***Dispute*** (including limitations, set-off claims) in accordance with the laws in force in the Province of Ontario and any applicable federal laws.

16.6.5 NO EQUITY

The arbitration tribunal shall not be authorized to decide *ex aequo et bono* or as *amiable compositeur*.

16.6.6 ARBITRAL INTERIM ORDERS

Subject to subparagraph 16.6.5 (No Equity) the arbitration tribunal shall have all the powers of a court at law or in equity, including the power to make interim orders, orders of injunction (either mandatory or prohibitory), rectification, expungement and orders for interest. However in no case will the final decision breach the strictures of subparagraph 16.6.5 (No Equity).

16.6.7 LOCATION

The proceedings shall take place in the city that was not the site of the mediation (or if there was no mediation, in the city that was not the site of the negotiation meeting), unless the ***Parties*** agree otherwise.

16.6.8 LANGUAGE

The language used in the proceedings shall be English.

16.6.9 NOTICES

All written communication shall be delivered to the ***Parties*** hereto in the manner provided for in Article 20.1 (Notice).

16.6.10 COSTS

The costs of the tribunal's fees and expenses shall be shared equally by the ***Parties***. The ***Parties*** shall bear their own costs except that the losing ***Party*** shall pay all costs, fees, levies and ***Taxes*** arising from and necessitated by the enforcement of the arbitration tribunal's award, including, without limitation, registration enforcement charges or other judicial levies.

16.7 Emergencies / Judicial Jurisdiction

The ***Parties*** are not precluded from bringing an application to a Court having jurisdiction for interim or interlocutory relief, in law or in equity, including, without limitation, injunctive relief, if such relief is urgently required to preserve the rights or property of either or both of the ***Parties***, pending the final determination of those rights in a subsequent arbitral proceeding as contemplated in this Article.

16.8 Final & Binding

Subject to the Code, the ***Parties*** hereto agree that the award and determination of the arbitration tribunal shall be:

16.8.1 final and binding on both ***Parties***;

16.8.2 without right of appeal;

16.8.3 the exclusive remedy between the ***Parties***, regarding any claims, counterclaims, issues or accountings presented or pled to the arbitration tribunal, and

the judgment upon the award rendered by the arbitration tribunal may be entered in any Court having jurisdiction thereof or having jurisdiction over either of the ***Parties***.

16.9 Arbitral Decision Deadline

The arbitration tribunal retainer shall contain the obligation that the arbitration tribunal render a written decision with reasons within thirty (30) days from the close of the hearing or submission of written argument.

16.9.1 If the facts and law are either too complicated or voluminous to allow a properly considered decision within thirty (30) days, then the decision shall be rendered in not less than one hundred and eighty (180) days, but the arbitrator shall notify the

Parties of the longer decision period by not later than the close of final arguments.

16.10 Power to Settle

The ***Parties'*** representatives at any arbitration throughout the arbitration shall be empowered or have sufficient delegated authority to resolve, compromise, negotiate or settle the ***Dispute*** submitted to arbitration, without seeking further instructions or approvals from any superiors or committees / corporate structures. The representatives shall either be the same persons as in paragraph 16.1.2 (Formal Negotiations) or their immediate subordinates.

16.10.1 Notwithstanding the foregoing, if the nature, seriousness or financial quantum of the ***Dispute*** in law or corporate policies/practices requires approval from the Board of Directors, or the Chief Public Health Officer, as the case may be, then, such approval shall be obtained within five (5) business days of the proffer of any settlement offer.

16.10.2 If applicable, the arbitration tribunal shall withhold its final decision until the ***Parties*** have ceased negotiating a settlement.

16.11 Adjournment to Empower Representative

Breach of paragraph 16.10 (Power to Settle, [Duly empowered representative]), shall entitle the other ***Party*** to seek an adjournment of the arbitration proceedings, to give the breaching ***Party*** time to appoint a duly empowered representative within the thirty (30) days. All costs directly traceable to such delay, including arbitration tribunal costs and the non-breaching ***Party's*** costs, shall be paid forthwith by the breaching ***Party***.

16.12 Deemed Abandonment

Failure of the breaching ***Party*** to appoint such a representative within the thirty (30) day period shall be deemed a withdrawal or abandonment of the ***Dispute*** by the breaching ***Party*** and the arbitrator shall render a formal decision, finding in favour of the non-breaching ***Party***.

16.13 General ADR Conditions

16.13.1 NO LITIGATION

If either ***Party*** has submitted the ***Dispute*** to court, which ***Dispute*** should properly have been submitted for resolution by arbitration, then the court filing

Party shall discontinue the court proceedings forthwith, upon notice from the other ***Party***, and both ***Parties*** shall remit the ***Dispute*** to arbitration hereunder.

16.13.2 OBLIGATIONS DURING ALTERNATE DISPUTE RESOLUTION (ADR)

During the progress of ADR, the ***Parties*** hereto shall continue to diligently perform their obligations under the ***License Agreement***.

16.13.3 PRIVILEGE

Neither ***Party*** shall be required to disclose documents that are privileged or created in contemplation of litigation. If a ***Party*** does disclose such a document during ADR, that disclosure shall not be construed as a waiver of any privilege unless the disclosing ***Party*** so elects in writing.

16.13.4 CONFIDENTIALITY

The ***Parties*** shall keep confidential the existence of the proceeding under this article, and any element of the ADR (including, without limitation, all conduct, statements, promises, offers, views, pleadings, briefs, documents, testimonies, identity of witnesses, submissions, awards and opinions, whether oral or written), made in the course of the ADR, except as may be lawfully required in judicial or regulatory proceedings relating to the arbitration or otherwise. Without limiting the generality of the foregoing, and for greater clarity, neither ***Party*** may make any publicly accessible statements / publications nor any shareholder or press announcements concerning any element of the ADR beyond the fact of the ADR.

16.13.5 ADR DISCLOSURES NOT ADMISSIBLE IN SUBSEQUENT PROCEEDINGS

Subject to subparagraph 16.13.6 (Normally Admissible Evidence), all conduct, statements, promises, offers, views and opinions, whether oral or written, made in the course of the ADR by either ***Party***, or the mediator or arbitrator, are not discoverable or admissible for any purposes, including impeachment, in any subsequent litigation or other proceedings involving the ***Parties***.

16.13.6 NORMALLY ADMISSIBLE EVIDENCE

Evidence that would otherwise be discoverable or admissible and was not created for an ADR, is not excluded from discovery or admission solely as a result of its use in the ADR.

16.14 Limitation

All ***Disputes*** must be submitted to ADR within one (1) year from the time of the facts giving rise to the ***Dispute***. Failure to submit the ***Dispute*** within the one (1) year period means a loss of all rights to submit the ***Dispute*** to ADR or litigation.

16.15 Material Breach

The failure, neglect or unwillingness of a ***Party*** to use or diligently participate in and prosecute a ***Dispute*** through ADR is a material breach of the ***License Agreement***.

17.0 INTENT AND INTERPRETATION

17.1 Entire Agreement

The ***License Agreement*** constitutes the entire and exclusive agreement between the ***Parties*** pertaining to the ***Commercialization*** and licensing and supersedes all prior agreements, conditions, obligations, understandings, and negotiations both written and oral. The ***License Agreement*** sets forth all obligations, undertakings, conditions, representations and warranties forming part of, or in any way affecting or relating to the ***Commercialization***. The ***Parties*** acknowledge that with respect to the ***Commercialization*** there are no agreements, obligations, undertakings, representations or warranties whether collateral, oral or written, between the Company and Canada other than those expressly set out in the ***License Agreement***.

17.2 No Third Parties

Neither the ***License Agreement*** or any provision thereof is intended to confer upon any person other than the ***Parties***, any rights or remedies hereunder.

17.3 No Pre-Contractual Inducing Representations

The ***License Agreement*** supersedes and revokes all negotiations, arrangements, letters of intent, offers, proposals, brochures, term sheets, representations, memoranda of understandings and information conveyed, whether oral or in writing or

electronically, between the ***Parties***, or any other person purporting to represent the Company or Canada. Each of the ***Parties*** agrees that:

17.3.1 neither has been induced to enter into the ***License Agreement*** by any representations whatsoever not set expressly forth in the ***License Agreement***;

17.3.2 neither has relied on any such representations;

17.3.3 no such representations shall be used in the interpretation or construction of the ***License Agreement***; and

17.3.4 no claims (including, without limitation, loss of profits, indirect, incidental, consequential damages and economic loss) arising directly or indirectly, from any such representation, negligent or otherwise, shall accrue in law or equity, or be pursued by the Company, and Canada shall have no liability for any such claims.

17.4 Due Diligence Search

The Company agrees that it has conducted its own due diligence examinations in order to satisfy itself of the full, true and plain disclosure of all facts pertinent to the ***Licensed Rights*** and all representations made by Canada.

17.5 Independent Legal Advice

It is acknowledged by the ***Parties*** that each has had legal advice to the full extent deemed necessary by each ***Party***. Furthermore, the ***Parties*** acknowledge that neither acted under any duress in negotiating, drafting and executing the ***License Agreement***.

17.6 No Adverse Presumption in Case of Ambiguity

There shall be no presumption that any ambiguity in the ***License Agreement*** be resolved in favour of either of the ***Parties***. For greater certainty, the *contra proferentum* rule shall not be applied in any interpretation of the ***License Agreement***.

17.7 Severability

If a jurisdiction declares, finds or holds any part of the ***License Agreement*** invalid, void, unenforceable or contrary to public policy for any other reason, then:

17.7.1 NON-MATERIAL

if the invalid provision is not material or fundamental to the ***License Agreement***, the invalid provision shall not affect the validity of the remainder which remainder shall continue if full force and effect and be construed as if the ***License Agreement*** had been executed without the invalid provision in that jurisdiction only;

17.7.2 MATERIAL

if the invalid provision is material to the ***License Agreement*** then that provision shall be "read down" or replaced with a provision which accomplishes, to such extent as is possible, the original legal and business purpose of such provision in a valid and enforceable manner, in that jurisdiction and the remainder of the ***License Agreement*** shall remain binding on the ***Parties***; and

17.7.3 FUNDAMENTAL

if the invalid provision is fundamental to the ***License Agreement***, including any of the elements of a bare license, then:

17.7.3.2 the jurisdiction which found the invalidity shall be deleted from the ***Territory***; or

17.7.3.3 if the jurisdiction cannot be deleted from the ***Territory***, or there is more than one jurisdiction, then the ***License Agreement*** shall terminate.

17.8 Successors and Assigns

The ***License Agreement*** will be for the benefit of and be binding upon the heirs, executors, administrators, permitted successors, permitted assigns, and permitted ***Affiliates*** of the Company and other legal representatives, as the case may be, of each of the ***Parties***. Every reference in the ***License Agreement*** to any ***Party*** includes the heirs, executors, permitted administrators, permitted successors, permitted assigns, and ***Affiliates*** and other permitted legal representatives of the ***Party***.

17.9 Plurality and Gender

Reference to a ***Party*** will be read as if all required changes in the singular and plural and all grammatical changes rendered necessary by gender had been made.

17.10 Not a Joint Venture

The *Parties* expressly disclaim any intention to create a partnership, joint venture or joint enterprise. The ***Parties*** acknowledge and agree that:

17.10.1 nothing contained in the ***License Agreement*** nor any acts of any ***Party*** shall constitute or be deemed to constitute the ***Parties*** as partners, joint venturers or principal and agent in any way or for any purpose;

17.10.2 no ***Party*** has the authority to act for, or to assume any obligation or responsibility on behalf of any other ***Party***; and

17.10.3 the relationship between the ***Parties*** is that of licensor and licencee.

17.11 Minister Not Fettered

Nothing in the ***License Agreement*** shall derogate or otherwise fetter the ability of Canada to regulate, administer, manage or otherwise deal with public health and all attendant matters thereto.

17.12 Federal Legislation

The application to the ***License Agreement*** of any Federal act or regulation includes any subsequent amendment, revision, substitution, consolidation to that act or regulation, notwithstanding that such amendment, revision or substitution occurred after the execution of the ***License Agreement*** or may have a retroactive effect.

17.13 Right to Legislate

Nothing in the ***License Agreement*** shall prohibit, restrict or affect the right or power of the Parliament of Canada to enact any laws whatsoever with respect to any area of law

for which the Parliament of Canada has legislative jurisdiction, even if the enactment of any such law affects the ***License Agreement***, its interpretation, or the rights, obligations, liabilities, vested or not, accrued or accruing, of the ***Parties***.

17.14 Compliance with Law

The ***Parties*** shall comply with all applicable laws, as those laws may be amended, revised, consolidated, substituted, from time to time, even if such amendment, revision, consolidation, substitution derogates prospectively or retroactivity

from the ***Parties'*** vested or accrued rights, obligations and liabilities under the ***License Agreement***.

17.15 No Implied Obligations

No implied terms or obligations of any kind, by or on behalf of either of the ***Parties***, shall arise from anything in the ***License Agreement***. The express covenants and agreements herein contained and made by the ***Parties*** are the only covenants and agreements upon which any rights against either of the ***Parties*** may be founded.

17.16 Access to Information

Notwithstanding any provision to the contrary in the ***License Agreement***, the Company acknowledges that Canada is subject to the Access to Information Act, R.S.C. 1985, c.A-1 and related acts, and may be required to release, in whole or in part, the ***License Agreement*** and any other information or documents in Canada's possession or control relating to the ***License Agreement*** and the ***Parties***.

17.17 Forum Conveniens & Applicable Laws

Subject to Article 16 (ADR) any ***Dispute***, shall be governed firstly by applicable Canadian Federal laws, and secondly by the laws of the Province of Ontario.
The ***Parties*** expressly exclude from the ***License Agreement***:

17.17.1 application of the United Nations Convention on Contracts for the International Sale of Goods;
17.17.2 International Sales of Goods Act; and
17.17.3 any conflict of laws, venue, forum non-conveniens, rules or principles which might refer ***Disputes*** to the laws of another jurisdiction.

17.18 Attornment

The ***License Agreement*** shall be governed by and construed in accordance with the laws in force in the Province of Ontario, Canada and shall be treated in all respect as an Ontario, Canada contract. Subject to Article 16 (Alternate ***Dispute*** Resolution (ADR)) the ***Parties*** irrevocably and unconditionally attorn to and submit to the exclusive jurisdiction of the courts of Ontario, Canada and all courts competent to hear appeals therefrom with respect to any ***Dispute*** now or hereinafter arising under the ***License Agreement***. The Parties waive any right each may have to object to an action being

brought in those courts including, without limitation, by claiming that the action has been brought in an inconvenient forum or that those courts do not have jurisdiction.

17.19 USA Jury Trial

If the ***License Agreement*** or any aspect of it becomes a subject of judicial proceedings whether in contract, tort, equity or otherwise, in the United States of America despite the ADR article and Forum Conveniens (paragraph 17.17), then the Company irrevocably waives any and all rights it has to a trial by jury in the United States. The Company agrees and consents that due to the technical and legal nature, including cross jurisdictional issues of the ***License Agreement*** or any aspect thereof, any such proceedings will be heard before a judge sitting alone.

17.20 USA Jury Trial / Treble Damages Addendum

For greater clarity, the Company waives any right to a trial by jury of any claim, demand action or caution of action

17.20.1 arising under the ***License Agreement***; or
17.20.2 in any way connect with or related or incidental to the dealings of the ***Parties*** in respect of the ***License Agreement*** or any other agreements or the transactions related hereto or thereto in each case whether now existing or hereafter;
17.20.3 whether in contract, tort, equity or otherwise.

The Company agrees and consents that any such claim, demand, action or cause of action shall be decided by a court without a jury. Canada may file an original counterpart of the ***License Agreement*** with the court as written evidence of the consent of the ***Parties*** to the waiver of their right to a trial by jury. In addition, the Company irrevocably waives any rights to triple/treble damages or punitive damages under U.S. or any other law.

17.21 Waiver of Counterclaims

The Company waives any and all of its rights to interpose any claims, deductions, setoffs or counterclaims of any nature in any ***Dispute*** with respect to the ***License Agreement***.

17.22 Due Diligence Audits

If in a subsequent transaction a third ***Party*** requires to review this ***License Agreement*** as part of a due diligence chain of title search, the Company hereby authorizes the release of this ***License Agreement*** subject to deleting any financial or proprietary or other ***Confidential Information*** contained herein.

17.23 Recitals Accurate

The ***Parties*** acknowledge the truth and accuracy of the recitals and further acknowledge that the recitals may be used by a court, mediator or arbitrator to help resolve any ***Dispute***.

17.24 Force Majeure

17.24.1 EVENTS

Subject to making all payments required under the ***License Agreement***, neither ***Party*** shall be in breach of any of its obligations under the ***License Agreement*** where the failure to perform or delay in performing any obligation is due, wholly or in part, directly or indirectly to the occurrence of a force majeure event including, without limitation:

17.24.1.2 war, whether declared or not, civil war, revolution, acts of piracy / terrorism, acts of sabotage;

17.24.1.3 natural disasters such as violent or destructive storms, cyclones, earthquakes, tidal waves floods, destruction by lightning;

17.24.1.4 explosions, fires, destruction of machines, factories, and any kind of installation;

17.24.1.5 boycotts, strikes and lock-outs of all kinds, go-slows, occupation of factories and premises, and work stoppages which occur in the enterprise of the ***Party*** seeking relief;

17.24.1.6 acts of governmental bodies, agencies, boards, whether lawful or unlawful other than those of the Public Health Agency of Canada,

but does not include:

17.24.1.7 the lack of regulatory or other approvals, licenses, permits and authorizations necessary for the performance of the ***License Agreement*** which are issued by a public authority of any kind whatsoever for which the

Company did not apply for or diligently prosecute;

17.24.1.8 the inability of the affected ***Party*** to obtain financing or any other financial inability on the part of either ***Party*** to meet its obligations under the ***License Agreement***;

17.24.1.9 force majeure events that the affected ***Party*** knew or should have reasonably known at the time of negotiating the ***License Agreement*** were probable or avoidable or the effects of which could be minimized, and the affected ***Party*** took no steps to deal with such force majeure events, including without limitation obtaining the appropriate insurance, using updated machinery;

17.24.1.10 the portion of the breach or delay due to the failure of the affected ***Party*** to take all necessary reasonable steps to minimize, overcome or control the effects of the force majeure event.

17.24.2 DUTY TO NOTIFY

The ***Party*** affected by a force majeure event as contemplated in subparagraph 17.24.1 (Force Majeure) shall:

17.24.2.2 give notice to the other ***Party*** of such force majeure and its effects on the affected ***Party***'s ability to perform as soon as practicable after the force majeure and its effects upon the affected ***Party***'s ability to perform become known to that ***Party***. Notice shall be given when the ground of relief ceases;

17.24.2.3 take all reasonable efforts to correct, compensate or minimize the effect of the force majeure event.

17.24.3 COMMENCEMENT OF RELIEF

The affected ***Party*** shall in the affected jurisdiction only:

17.24.3.2 be excused of its obligations under the ***License Agreement*** to the extent necessitated by the force majeure event from the time of the force majeure event or if notice was not given as soon as practical, from the receipt of such notice. Failure to give notice makes the failing ***Party*** liable in damages for losses suffered by the other ***Party*** which otherwise could have been

avoided; and

17.24.3.3 complete or continue performance of its obligations and duties under the ***License Agreement*** as soon as practical after the cessation, removal, or overcoming of the force majeure event.

17.24.4 TERMINATION OF AGREEMENT

If the force majeure event continues in excess of sixty (60) consecutive days, or in the aggregate 60 days over any consecutive 200 days, then at any time thereafter Canada shall have the option to renegotiate the ***License Agreement*** with the Company reasonably and in good faith. If the ***Parties*** are unable to agree to the terms of the proposed amended ***License Agreement*** within 60 days from the notice to negotiate, then the ***License Agreement*** may be terminated by Canada on the 61st day.

17.24.5 POSTPONEMENT OF OBLIGATIONS

Any obligations of a ***Party*** under the ***License Agreement*** shall be postponed automatically to the extent and for the period and only within the jurisdiction or jurisdictions that the affected ***Party*** is prevented from meeting those obligations by reason of any cause beyond its reasonable control (other than lack of funds and applicable regulatory approval). The affected ***Party*** shall immediately notify the other ***Party*** of the commencement, nature of such cause and probable consequence. The affected ***Party*** shall also use its

reasonable best efforts to render performance in a timely manner, utilizing all resources reasonably required in the circumstances.

17.25 Waiver

No condoning, excusing, or overlooking by either of the ***Parties*** of any default by the other ***Party***, at any time or times, in performing or observing any of the ***Parties'*** respective covenants, will operate as a waiver renunciation, surrender, of or otherwise affect the rights of the ***Parties*** in respect of any continuing or subsequent default. No waiver of these rights will be inferred from anything done or omitted by the ***Parties***, except by an express waiver in writing.

17.26 No Estoppel Due to Third Party Practices

No custom, practice or usage regarding other ***License Agreements*** between Canada and other ***Parties*** shall preclude at any time the strict enforcement of the ***License Agreement*** by Canada or the Company.

17.27 Contract Always Speaks

Where a matter or thing is expressed in the present tense, it shall be applied to the circumstances as they arise, so that effect may be given to the ***License Agreement*** according to its true spirit, intent and meaning.

17.28 Time is of the Essence

Time is of the essence in the ***License Agreement*** with respect to the financial and ***Commercialization*** obligations of the Company.

17.29 Headings

17.29.1 All headings in the ***License Agreement*** have been inserted as a matter of convenience and for reference only, and in no way define, limit, enlarge, modify, the scope or meaning of the ***License Agreement*** or any of its provisions.

17.29.2 Nevertheless an arbitrator or Judge may use any or all of the table of contents, recitals, and headings when reviewing the covenants, statements, representations & warranties and conditions subsequent to better understand the commercial and legal intent of the ***License Agreement's*** provisions.

17.30 Internal References

Any reference in the ***License Agreement*** to an Article, paragraph, sub-paragraph, will mean an Article, paragraph or sub-paragraph of the ***License Agreement***, unless otherwise expressly provided.

17.31 Precedence Over Appendices

If there is a conflict or ambiguity between the ***License Agreement*** proper and any appendix thereto, the interpretation consistent with ***License Agreement*** proper (taking into consideration the statements in the recitals and headings) shall prevail and apply, notwithstanding any wording to the contrary in the applicable appendix.

17.32 Appendices

Subject to paragraph 17.31 (Precedent Over Appendices) the documents attached hereto as Appendix A, B, C and D form an integral part of this ***License Agreement*** as fully as if they were set forth herein *in extenso*, and consist of:

Appendix “A” — DESCRIPTION OF THE LICENSED RIGHTS Appendix “B” — CONFIDENTIALITY AGREEMENTS Appendix “C” — BUSINESS PLAN Appendix “D” — AFFILIATES

18.0 LEGAL RIGHTS

18.1 Amendments

No modification or waiver of any provision of the ***License Agreement*** will be inferred from anything done or omitted by either of the ***Parties***, except by an express amendment in writing, duly executed by the ***Parties*** in advance.

18.2 No Assignment Without Consent

The ***License Agreement*** is personal to the Company. The Company shall not assign the ***License Agreement*** or any of the Company’s rights, duties or obligations under the ***License Agreement*** to a third party without the prior written consent of Canada, such consent not to be unreasonably withheld. Any attempt to assign this ***License Agreement*** or any of the Company’s rights, duties or obligations under the ***License Agreement*** without the prior written consent of Canada is void.

18.3 Mode of Assignment / Approval Conditions

Without derogating from paragraph 18.2 (Assignment), the Company shall not assign (or transfer, sell, encumber, pledge, grant a security interest sub-license or otherwise deal) or permit any such assignment, in whole or in part, of the ***License Agreement*** or any of its interest, rights or obligations hereunder, whether such assignment takes place by way of:

18.3.1 sale of assets;

18.3.2 sale of shares;

18.3.3 amalgamation, merger or other reorganization of the Company;

18.3.4 merger, transfer, conversion, assignment, redemption, issuance, sale, cancellation, pledge, conversion or other dealings with any securities of the Company;

18.3.5 operation of law;

18.3.6 acquisition by a person or persons acting in concert of a majority interest of the securities of the Company by a person or persons acting in concert who did not hold such a majority interest at the time of the initial public offering (IPO) or at any time after the IPO.

18.3.7 operation of contract; or

18.3.8 otherwise in any manner or structure whatsoever;

without the prior written consent of Canada, which consent subject to subparagraph 18.3.9 will not be unreasonably withheld.

18.3.9 Any consent from Canada shall be contingent and effective only upon receipt by Canada of payment of [*].

18.3.10 Consent to any assignment will not be construed as consent to any other assignment.

18.4 No Consent — Material Breach

Failure of the Company to obtain the prior written consent of Canada to any assignment shall be deemed to be a material breach of the ***License Agreement***.

18.5 Assignment Prejudicial - Compensation

It will not be unreasonable for Canada to refuse to consent to any assignment if it is foreseeable that the assignment might negatively affect Canada in any way, or put Canada in breach of any contract with a third party or derogate from the ***Commercialization***. Notwithstanding the foregoing, Canada may still consent in exchange for payment of both [*].

18.6 No Comfort Letter

Notwithstanding anything to the contrary in the ***License Agreement***, Canada shall be under no obligation whatsoever to sign any a comfort letter or other undertaking to a third party for the benefit of the Company. If Canada so elects pursuant to

its unfettered discretion, then the Company shall pay or provide security in the amount of liability so accepted or incurred by Canada.

18.7 Subcontracting

The Company has the right to subcontract any portion, but not all, of the ***License Agreement,*** subject to the following:

18.7.1 subcontracting activities (including subcontracts entered into with contract research organizations) shall be carried out by the Company in a manner that is consistent with the Company's obligations under paragraphs 2.4 to 2.7 of the ***License Agreement***;

18.7.2 the Company shall notify Canada in writing of any significant subcontracts or subcontractors of whom the Company is aware may have an interest in the technology or a collaboration with Canada;

18.7.3 the subcontract cannot be a *de facto* assignment; and

18.7.4 no rights, obligations, power or control vested in the Company shall be contingently or otherwise transferred to any third party.

18.8 No Third Party Rights

Nothing expressed or implied in the ***License Agreement*** is intended to, or shall be construed to confer on or give to, any person other than the ***Parties***, any rights or remedies under or by reason of the ***License Agreement***.

18.9 Remedies Cumulative

All rights, powers and remedies provided by the ***License Agreement*** are cumulative with, and not exclusive of, the rights, powers or remedies provided by law or equity independently of the ***License Agreement***.

18.10 Mutual Assistance

The ***Parties*** will at all times hereafter, upon every reasonable request of the other, make, do, and execute or cause to be procured, made, done, and executed, all such further acts, deeds and assurances for the carrying out of the terms, covenants and agreements of the ***License Agreement***, according to the true intent and meaning of the ***License Agreement***. These obligations shall continue post termination or expiry until all pre and post termination obligations are satisfied.

18.11 Counterpart

The ***License Agreement*** may be executed simultaneously in counterpart, each of which shall be deemed an original, but all of which together shall constitute one and the same instrument.

19.0 CROWN GENERAL

19.1 No Bribes

The Company warrants that no bribe, gift, or other inducement has been paid, given, promised or offered to any Government official or employee for the obtaining of this ***License Agreement***.

19.2 No Share to Members of Parliament

Pursuant to the Parliament of Canada Act, R.S.C. 1985, c.P-1, no member of the House of Commons or Senate will be admitted to any share or part of the ***License Agreement*** or to any benefit arises from the ***License Agreement***.

19.3 Public Office Holders

It is a term of this ***License Agreement*** that no former public Office holder, who is not in compliance with the post employment provisions of the Conflict of Interest and Post Employment Code for Public Office Holders, shall derive a direct benefit from this ***License Agreement***.

20.0 NOTICE

20.1 Addresses / Contacts

Wherever in this ***License Agreement*** it is required or permitted that notice or demand be given, or served by either ***Party*** to or on the other ***Party***, such notice or demand will be in writing and will be validly given or sufficiently communicated if hand delivered or forwarded by certified mail, priority post mail, telegram, or facsimile or sent by overnight delivery by a nationally recognized courier as follows:

The addresses for delivery are:

To the Company:

Nicholas Vahanian Chief Medical Officer BioProtection Systems Corporation 2901 S.

Loop Dr., Suite 3360 Ames, IA, USA 50010 Telephone: (515) 598-2922 Facsimile: (515) 296-3820 Email: nvahanian@linkp.com

To Canada:

Dorothea Blandford, PhD Director, Intellectual Property Management & Business Development Operations Public Health Agency of Canada 1015 Arlington Street, Suite 2420 Winnipeg, Manitoba Canada R3E 3R2 Telephone: (204) 789-2096 Facsimile: (204) 789-2097 Email: dorothea_blanciford@hc-sc.dc.ca

The Parties shall send an e-mail version of the notice or demand at least 24 hours prior to the hard or facsimile copy, but failure to send the email version does not invalidate or otherwise make subsequent service of the notice defective,

20.2 Deemed Delivery

Notice will be deemed to have been delivered:

20.2.1 if delivered by hand, upon receipt;

20.2.2 if sent by electronic transmission, forty-eight (48) hours after the time of confirmed transmission, excluding from the calculation weekends and public holidays;

20.2.3 if sent by certified mail, four (4) days after the mailing thereof, provided that if there is a postal strike or other disruption, such notice will be delivered by hand or electronic transmission.

20.3 Change of Address

The ***Parties*** may change their respective addresses for delivery by delivering notice of change as provided in this paragraph.

21.0 EXECUTION

IN WITNESS WHEREOF this ***License Agreement*** has been executed in duplicate by the duly authorized representatives of the ***Parties***, on the date(s) set out below.

FOR HER MAJESTY THE QUEEN IN RIGHT OF CANADA:

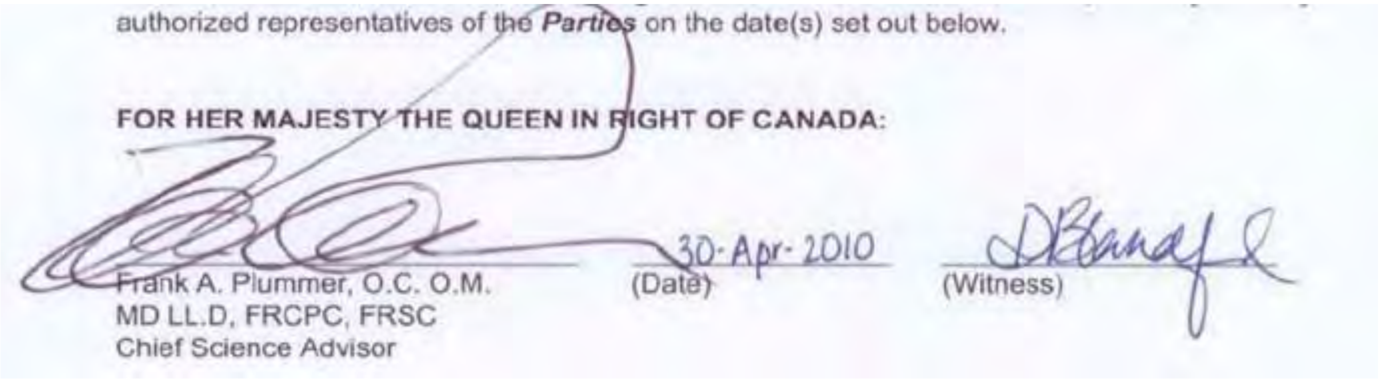
authorized representatives of the *Parties* on the date(s) set out below.

FOR HER MAJESTY THE QUEEN IN RIGHT OF CANADA:

30-Apr-2010

Frank A. Plummer, O.C. O.M. (Date) (Witness)
MD LL.D, FRCPC, FRSC
Chief Science Advisor

Frank A. Plummer, O.C. O.M.
MD LL.D, FRCPC, FRSC
Chief Science Advisor

FOR THE COMPANY:

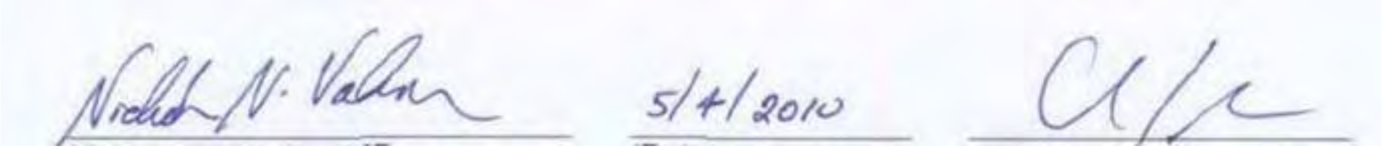

Nicholas Vahanian, MD
Chief Medical Officer

I have authority to bind the corporation

APPENDIX "A" DESCRIPTION OF THE *LICENSED RIGHTS*

[*]Recombinant Vesicular Stomatitis Virus Vaccines for Viral Hemorrhagic Fevers **[*]**.

[*]Recombinant Vesicular Stomatitis Virus Vaccines for Viral Hemorrhagic Fevers **[*]**.

[*]Recombinant Vesicular Stomatitis Virus Vaccines for Viral Hemorrhagic Fevers **[*]**.

APPENDIX "B" CONFIDENTIALITY AGREEMENTS

(APPENDED OVER THE NEXT 8 PAGES)

Public Health Agency of Canada / Agence de la santé publique du Canada

Office of Director
Business Development and Operations
National Microbiology Laboratory
1015 Arlington Street
Winnipeg, Manitoba R3E 3R2

April 14, 2010

Nicholas Vahanian
BioProtection Systems Corporation
Iowa Stale University Research Park,
2901 South Loop Drive, Suite 3360,
Ames, Iowa 50010

RE: Non Disclosure Agreement dated, November 18, 2008 between the Public Health Agency of Canada (referred to as "PHAC"), and BioProtection Systems Corporation (referred to as "BPS").

Dear Dr Vahanian,

PHAC and BPS have executed a Non Disclosure Agreement dated November 18, 2008. The parties hereby agree to amend the Agreement as follows:

i) Section 9C, "The term of this Agreement shall commence on its effective date and remain in force for **eighteen (18)** months thereafter, except that the Agreement shall remain effective with respect to the confidential information disclosed under this Agreement for the remainder of any period of confidentiality pursuant to subparagraph 9b above."

Shall be replaced by

Section 9C "The term of this Agreement shall commence on its effective date and remain in force for **sixty (60)** months thereafter, except that the Agreement shall remain effective with respect to the confidential information disclosed under this Agreement for the remainder of any period of confidentiality pursuant to subparagraph 9b above."

All other terms and conditions of the Agreement will remain in full force and effect and

shall continue the duration of the Agreement. This letter, upon execution by both parties, shall form part of the Agreement and the two documents shall be read together.

If the foregoing amendment is satisfactory, please counter sign this letter on behalf of the Participants in the spaces provided, and return the signed letter to our office via electronic PDF copy to sabrina_choma@phac-aspc.gc.ca

Sincerely,

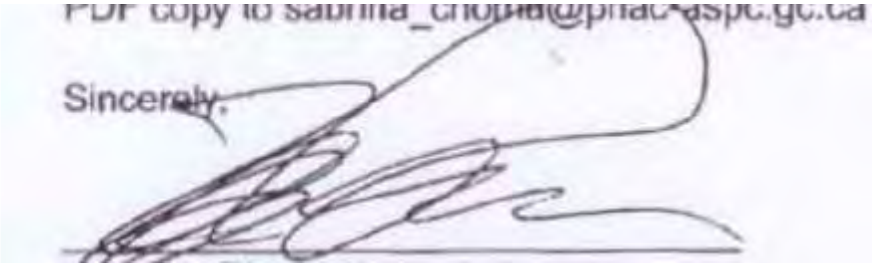

Frank A. Plummer OC, MD, LL.D, FRCP(C), FRSC
Scientific Director General
National Microbiology Laboratory

Acknowledged and agreed to on behalf of BPS:

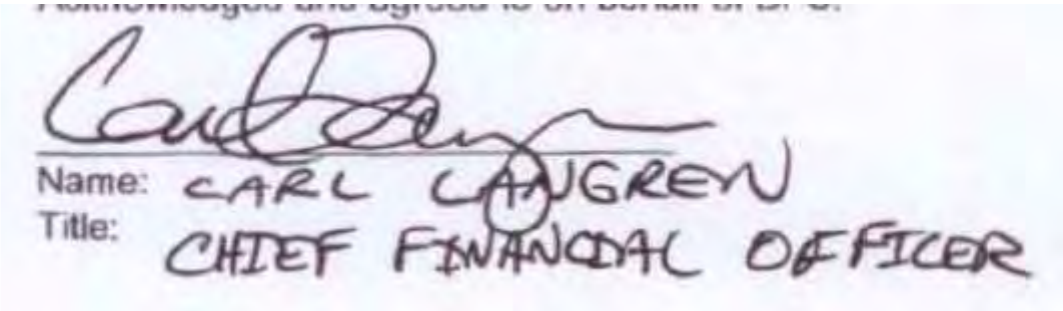

Name: Carl Langren
Title: Chief Financial Officer

Public Health Agency of Canada | Agence de santé publique du Canada

CONFIDENTIAL DISCLOSURE AGREEMENT

THE PARTIES ARE: Her Majesty the Queen in Right of Canada as represented by the Minister of Health ("Public Health Agency of Canada")

Whose address is:
National Microbiology Laboratory
Canadian Science Centre for Human and Animal Health Canada
1015 Arlington Street, Winnipeg, MB R3E 3R2, CANADA
(called "**PHAC**") OF THE FIRST PART

AND: BioProtection Systems Corporation

Whose address is
Iowa State University Research Park, 2901 South Loop Drive,
Suite 3360, Ames Iowa 50010
(called the "**Participant**") OF THE SECOND PART

Effective Date: November 1, 2008

In order to protect certain confidential information the Parties identified above, agree on terms about confidentiality which fairly protects both parties.

1. Disclosing Party: The party(ies) disclosing confidential information ("Disclosing Party") is/are: Public Health Agency at Canada, National Microbiology Laboratory, 1015 Arlington Street, Winnipeg. MB 133E 3R2, and BioProtection Systems Corporation, 2901 South Loop Drive, Suite 3360, Ames, Iowa 50010.

2. Primary Representative: The representative(s) of each party for coordinating the disclosure and/or receipt of confidential information are: Dr. Steven Jones and Dr. Dorothea Blandford and Dr. Nicholas Vahanian.

3. Description of Confidential Information: The subject matter of the confidential information disclosed under this Agreement is described as:
Public Health Agency of Canada: [*].
Participant: scientific and technical information relating to the pipeline products; business information.

4. Use of Confidential Information: The party receiving the confidential information ("Recipient") shall keep the confidential information in strict confidence and shall make use of the confidential information only for the following purpose: to discuss scientific and business arrangements in view of negotiating a license agreement.

5a. Standard of Care: Recipient shall protect the disclosed confidential information by using the same degree of care, but no less than a reasonable degree of care, to prevent the unauthorized use, dissemination, or publication of the confidential information, as Recipient uses to protect its own confidential information of a like nature.

5b. In particular, and without limiting the generality of the foregoing, Recipient shall not copy, reproduce, divulge, publish or circulate (or permit anyone else to do so) any of the confidential information disclosed to it by the Disclosing Party, except to such of its employees [and/or contractors and consultants] as may require access to the confidential information on a strict need-to-know basis for the uses contemplated in paragraph 4.

6. Markings: Recipient's obligations shall extend to confidential information that is described in paragraph 3, and that (a) if set out in written, graphical, photographic or other tangible form (including, without limitation thereto, machine readable object code), is marked "Confidential' or "Proprietary" by the Disclosing Party, or (b) if disclosed orally, is identified as confidential or proprietary at the time of disclosure and a written summary thereof marked 'Confidential" or 'Proprietary" is furnished by the Disclosing Party to Recipient within thirty (30) days after such oral disclosure.

7. Exclusions: This Agreement imposes no obligation upon Recipient with respect to information that: (a) was in Recipient's possession before receipt from the Disclosing Party; (b) is or becomes a matter of public knowledge through no fault of Recipient; (c) is rightfully received by Recipient from a third party without a duty of confidentiality; (d) is disclosed by the Disclosing Party to a third party without a duty of confidentiality on the third party; (e) is independently developed by Recipient; (f) is disclosed under operation of law, including the Access to Information Act of Canada; or (g) is disclosed by Recipient with the Disclosing Party's prior written approval.

8. Warranty: Each Disclosing Party warrants that it has the right to make the disclosures under this Agreement.

NO OTHER WARRANTIES ARE MADE BY EITHER PARTY UNDER THIS AGREEMENT. ANY INFORMATION EXCHANGED UNDER THIS AGREEMENT IS PROVIDED "AS IS".

NEITHER PARTY PROVIDES ANY OTHER REPRESENTATION, WARRANTY, ASSURANCE OR GUARANTEE OF ANY KIND WITH RESPECT TO THE

CONFIDENTIAL INFORMATION IT DISCLOSES.

9a. Rights: Neither party acquires any intellectual property rights under this Agreement except the limited rights necessary to carry out the purposes set forth in paragraph 4. This Agreement shall not restrict reassignment of Recipient's employees.

9b. The obligations set out in paragraphs 4 and 5 above shall become effective with respect to any confidential information immediately upon its disclosure by the Disclosing Party to Recipient and shall continue for a period of three (3) years thereafter.

9c. The term of this Agreement shall commence on its effective date and remain in force for 18 months thereafter, except that the Agreement shall remain effective with respect to the confidential information disclosed under this Agreement for the remainder of any period of confidentiality pursuant to subparagraph 9b above.

9d. Upon request made by the Disclosing Party during the term of the Agreement, Recipient shall return the confidential information and all copies thereof to the Disclosing Party or, at the option of the Disclosing Party, destroy the confidential information and all copies thereof, and Recipient shall certify in writing within five (5) days of the receipt of the request from the Disclosing Party that it has complied with that request.

Miscellaneous

10. The only terms concerning confidentiality relating to the information described in paragraph 3 are in this Agreement and in the Access to Information Act of Canada.

11. This Agreement imposes no obligation on either party to purchase, sell, licence, transfer or otherwise dispose of any technology, services or products, and neither this Agreement nor the disclosure or receipt of confidential information under this Agreement constitutes or implies any undertaking or commitment by either party to enter into any further activity, arrangement or course of action with the other party or with any third party.

12. Both parties shall adhere to all applicable laws, regulations and rules relating to the export of technical data, and shall not export or re-export any technical data, any products received from the Disclosing Party, or the direct product of such technical data to any prescribed country listed in such applicable laws, regulations and rules unless properly authorized.

13. This Agreement does not create any agency or partnership relationship.

14. This Agreement cannot be modified except by a document signed by both Parties that explicitly refers to this Agreement.

SIGNED by the Participant in duplicate at Ames, Iowa

This 18th day of November 2008

BioProtection Systems Corporation

Per [signature]

Nicholas N. Vahanian, M.D.
Chief Medical Officer,

SIGNED by the Public Health Agency of Canada in duplicate at Winnipeg, Manitoba

This 1 day of November 2008

Her Majesty the Queen in Right of Canada as Represented by the Minister of Health

Per: [signature]

Frank A Plummer, OC, MD, LLD, FRCP (C), FRSC
Scientific Director General
National Microbiology Laboratory

Public Health Agency of Canada | Agence de santé publique du Canada

CONFIDENTIAL DISCLOSURE AGREEMENT

THE PARTIES ARE: Her Majesty the Queen in Right of Canada as represented by the Minister of Health ("Public Health Agency of Canada")

Whose address is
National Microbiology Laboratory
Canadian Science Centre for Human and Animal Health Canada
1015 Arlington Street, Winnipeg, MB R3E 3R2, CANADA
(called "**PHAC**") OF THE FIRST PART

AND: BioProtection Systems Corporation

Whose address is
Iowa State University Research Park, 2901 South Loop Drive,
Suite 3360, Ames Iowa 50010
(called the "**Participant**') OF THE SECOND PART

Effective Date: May 1, 2007

In order to protect certain confidential information the Parties identified above, agree on terms about confidentiality which fairly protects both parties.

1. Disclosing Party: The party(ies) disclosing confidential information ("Disclosing Party") is/are: Public Health Agency of Canada, National Microbiology Laboratory, 1015 Arlington Street, Winnipeg, MB R3E 3R2, and BioProtection Systems Corporation, 2901 South Loop Drive, Suite 3360, Ames, Iowa 50010.

2. Primary Representative. The representative(s) of each party for coordinating the disclosure and/or receipt of confidential information are: Dr. Heinz Feldmann and Dr. Dorothea Blandford and Dr. Nicholas Vahanian.

3. Description of Confidential Information: The subject matter of the confidential information disclosed under this Agreement is described as:
Public Health Agency of Canada: [*]
Participant: scientific and technical information relating to the pipeline products: business information.

4. Use of Confidential Information: The party receiving the confidential information ("Recipient") shall keep the confidential information in strict confidence and shall make use of the confidential information only for the following purpose: to discuss scientific and business arrangements in view of negotiating a license agreement.

5a. Standard of Care: Recipient shall protect the disclosed confidential information by using the same degree of care, but no less than a reasonable degree of care, to prevent the unauthorized use, dissemination, or publication of the confidential information, as Recipient uses to protect its own confidential information of a like nature.

5b. In particular, and without limiting the generality of the foregoing, Recipient shall not copy, reproduce, divulge, publish or circulate (or permit anyone else to do so) any of the confidential information disclosed to it by the Disclosing Party, except to such of its employees [and/or contractors and consultants] as may require access to the confidential information on a strict need-to-know basis for the uses contemplated in paragraph 4.

6 Markings: Recipient's obligations shall extend to confidential information that is described in paragraph 3, and that (a) if set out in written, graphical, photographic or other tangible form (including, without limitation thereto, machine readable object code), is marked "Confidential" or "Proprietary" by the Disclosing Party, or (b) if disclosed orally, is identified as confidential or proprietary at the time of disclosure and a written

summary thereof marked “Confidential” or “Proprietary” is furnished by the Disclosing Party to Recipient within thirty (30) days after such oral disclosure.

7. Exclusions: This Agreement imposes no obligation upon Recipient with respect to information that: (a) was in Recipient’s possession before receipt from the Disclosing Party; (b) is or becomes a matter of public knowledge through no fault of Recipient; (c) is rightfully received by Recipient from a third party without a duty of confidentiality: (d) is disclosed by the Disclosing Party to a third party without a duty of confidentiality on the third party; (e) is independently developed by Recipient; (1) is disclosed under operation of law, including the Access to information Act of Canada; or (g) is disclosed by Recipient with the Disclosing Party’s prior written approval.

8. Warranty: Each Disclosing Party warrants that it has the right to make the disclosures under this Agreement.

NO OTHER WARRANTIES ARE MADE BY EITHER PARTY UNDER THIS AGREEMENT, ANY INFORMATION EXCHANGED UNDER THIS AGREEMENT IS PROVIDED “AS IS”.

NEITHER PARTY PROVIDES ANY OTHER REPRESENTATION, WARRANTY, ASSURANCE OR GUARANTEE OF ANY KIND WITH RESPECT TO THE CONFIDENTIAL INFORMATION IT DISCLOSES.

9a. Rights: Neither party acquires any intellectual property rights under this Agreement except the limited rights necessary to carry out the purposes set forth in paragraph 4. This Agreement shall not restrict reassignment of Recipient’s employees.

9b. The obligations set out in paragraphs 4 and 5 above shall become effective with respect to any confidential information immediately upon its disclosure by the Disclosing Party to Recipient and shall continue for a period of three (3) years thereafter.

9c. The term of this Agreement shall commence on its effective date and remain in force for 18 months thereafter, except that the Agreement shall remain effective with respect to the confidential information disclosed under this Agreement for the remainder of any period of confidentiality pursuant to subparagraph 9b above.

9d. Upon request made by the Disclosing Party during the term of the Agreement, Recipient shall return the confidential information and all copies thereof to the Disclosing Party or, at the option of the Disclosing Party, destroy the confidential information and all copies thereof, and Recipient shall certify in writing within five (5) days of the receipt of the request from the Disclosing Party that it has complied with that request.

Miscellaneous

10. The only terms concerning confidentiality relating to the information described in paragraph 3 are in this Agreement and in the Access to Information Act of Canada.

11. This Agreement imposes no obligation on either party to purchase, sell, licence, transfer or otherwise dispose of any technology, services or products, and neither this Agreement nor the disclosure or receipt of confidential information under this Agreement constitutes or implies any undertaking or commitment by either party to enter into any further activity, arrangement or course of action with the other party or with any third party.

12. Both parties shall adhere to all applicable laws, regulations and rules relating to the export of technical data, and shall not export or re-export any technical data, any products received from the Disclosing Party, or the direct product of such technical data to any prescribed country listed in such applicable laws, regulations and rules unless properly authorized.

13. This Agreement does not create any agency or partnership relationship

14. This Agreement cannot be modified except by a document signed by both Parties that explicitly refers to this Agreement.

SIGNED by the Participant in duplicate at Ames, Iowa	SIGNED by the Public Health Agency of Canada in duplicate at Winnipeg, Manitoba
This 30th day of April 2007	This 27 day of April 2007
**BioProtection Systems Corporation**	**Her Majesty the Queen in Right of Canada as Represented by the Minister of Health**
Per Nicholas N. Vahanian	Per: [signature]
Nicholas N. Vahanian, M.D.<br>Chief Medical Officer,	Frank A Plummer, OC, MD, LLD, FRCP (C), FRSC<br>Scientific Director General<br>National Microbiology Laboratory

APPENDIX "C" BUSINESS PLAN

(TO FOLLOW WITHIN 30 DAYS OF EXECUTION)

APPENDIX "D" AFFILIATES

NewLink Genetics Corporation, 2901 South Loop Drive, Suite 3900, Ames, Iowa, USA 50010

PROJECT COMPLETION REPORT

Canadian Safety and Security Program

Project Completion Report

Development of Live Replicating Viruses as Vaccines and Therapies for Viral Hemorrhagic Fever Viruses

CSSP 05-0078RD

24 March 2014

PROJECT SUMMARY

1. Background

Filoviruses [Zaire Ebola virus (ZEBOV) and Marburg virus (MARV)] are considered serious threats as agents of biological warfare for a number of reasons. The agents are highly infectious causing highly virulent hemorrhagic fevers in humans with a fatality rate approaching 90%. Although transmission is primarily through direct contact with infected bodily fluids, it has been reported that the former Soviet Union produced large quantities of MARV in a formulation directed to large-scale aerosol dissemination, and that the simple addition of glycerine to the virus preparation made MARV as stable as Influenza virus in aerosol phase. Furthermore, it has been shown experimentally that ZEBOV is infectious following oral and ocular exposure of nonhuman primates as well as by aerosol. Therefore, an effective MARV and ZEBOV vaccine should be capable of rapidly inducing both systemic and mucosal immunity, since the intentional use of these agents would probably cause mucosal infection after small particle aerosol dispersion. Unfortunately, there is no known clinically available treatment or effective vaccine, which makes this an absolute priority deliverable for biodefense.

We generated a live attenuated recombinant vesicular stomatitis virus (VSV) in which the native glycoprotein gene (VSVG) has been deleted and functionally replaced with the glycoprotein (GP) gene from ZEBOV (VSVΔG/ZEBOVGP) (Figure 1). One dose of (VSVΔG/ZEBOVGP resulted in complete protection of mice from challenge with a mouse-adapted variant of ZEBOV (MA-ZEBOV). Subsequently these vaccines were shown to protect nonhuman primates from challenge with 1000LD50 of ZEBOV demonstrating that these vaccines are an effective measure for the prevention of a filovirus infection. The purpose of this CSSP project was to take the VSVΔG/ZEBOVGP vaccine forward in the process of being approved for prevention of Ebola virus infections in humans. To that end the CSSP funded the production of a pharmaceutical current good manufacturing practices (cGMP) grade VSVΔG/ZEBOVGP vaccine that can be used in clinical trials. It also funded the continuing research into the determination of the correlates of immune protection of an Ebola virus infection, as well as the development of biological assays that would be needed in the pre-clinical and clinical trials.

Figure 1: *Design of the VSVΔG/ZEBOVGP vaccine.* The glycoprotein gene from VSV was replaced with the glycoprotein gene from ZEBOV. This produced a recombinant VSV that had the same bullet structure as VSV but with the ZEBOV glycoprotein on the surface of the virus.

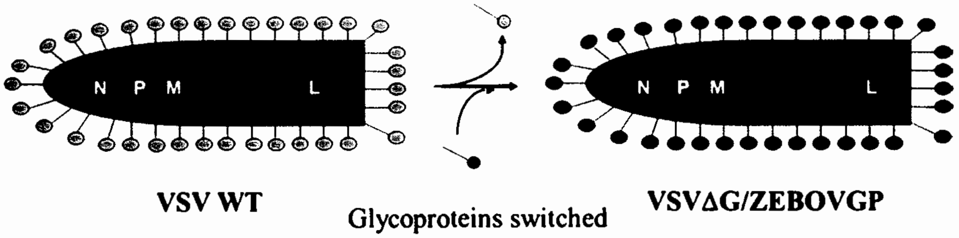

2. Methodology

There were two general objectives for this project (see figure #2). The first was to manufacture a pharmaceutical grade cGMP VSVΔG/ZEBOVGP vaccine that can be used in clinical trials, and can also be stockpiled in the event of an outbreak or bioterrorism event. The second objective was to continue developing assays to support the application of the vaccine for human use to Health Canada (HC) and the United States Food and Drug Administration (FDA); and also to conduct research on the VSVΔG/ZEBOVGP vaccine and EBOV in order to determine the correlates of immune protection.

The initial research grade vaccine was generated by the Public Health Agency of Canada (PHAC). PHAC then used a nonhuman primate (NHP) model to test the effectiveness of the vaccine by challenging the NHPs with EBOV. The vaccine

was 100% protective. It was at this point that the CSSP was needed to produce a pharmaceutical grade vaccine that could be used in humans. The manufacture of the vaccine was the work of the PHAC and two private pharmaceutical industry companies in Germany, Plasmid Factory, and IDT Biologika GmbH. The Plasmid Factory was contracted to generate the cGMP plasmids used to make the VSVΔG/ZEBOVGP vaccine. The plasmids were then used by PHAC to produce the initial culture of the vaccine. This culture was then sent to IDT Biologika, who was contracted to produce 1000-2000 vials of cGMP VSVΔG/ZEBOVGP that could be used for the clinical trials. The PHAC then tested the efficacy of the vaccine in non-human primates to make sure that the newly formulated vaccine was as effective as the research grade.

While the cGMP vaccine was being manufactured PHAC continued with researching the correlates of immune protection for an EBOV infection; developing vaccine and post-exposure protocols for VSVΔG/EBOVGP; and developing assays that could be used in the pre-clinical toxicology studies and clinical trials in order to support application of the vaccine for human use to the FDA and HC. This work was done in collaboration with our partners, Health Canada and the United States of America Medical Research Institute for Infectious Diseases (USAMRIID). Many of the early NHP studies were conducted at USAMRIID. However, later on PHAC was able to utilize the NHP colony at HC in Ottawa and the NHP studies began at the biosafety level 4 (BSL4) laboratory at the National Microbiology Laboratory (NML) in Winnipeg.

Figure #2: Overview of Project. There are two branches to the project, manufacturing, and research.

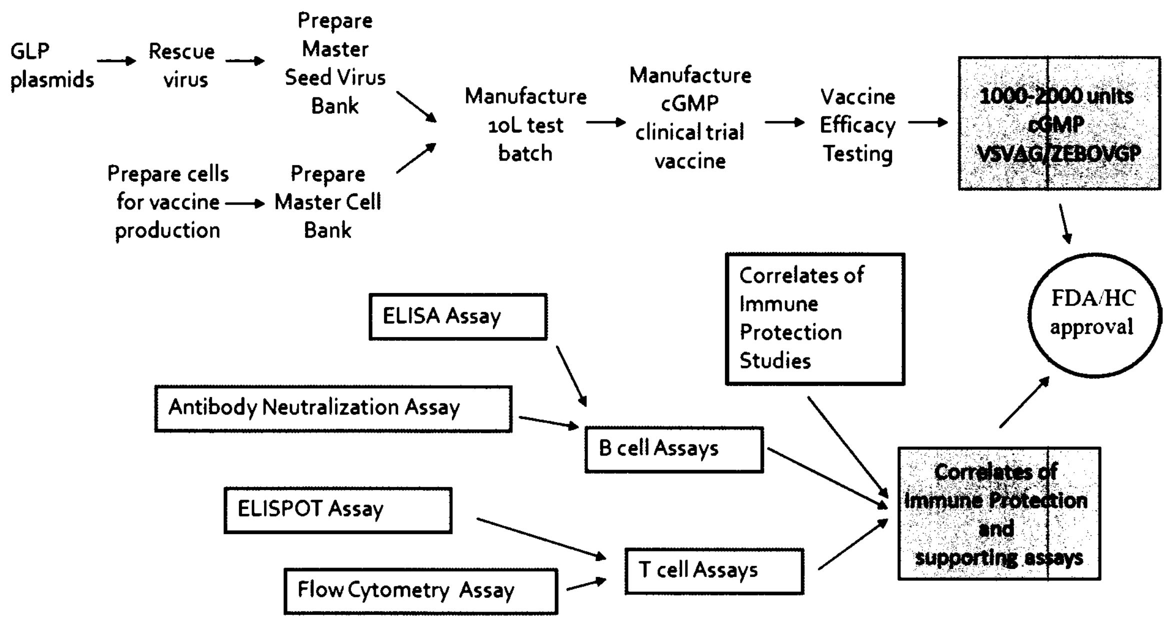

3. Results

Our goal was to generate a vaccine that effectively prevented an Ebola virus infection. To get a vaccine to market is a long and expensive process of which we have now taken one giant step towards fulfilling. A group of international Ebola virus experts rated VSVΔG/ZEBOVGP as the top vaccine candidate and would like to see it move forward. With the production of the cGMP vaccine the CSSP project has allowed the vaccine to go from the research and development stage, and progress to the clinical trials. The most critical goal of this project was obtaining 1000-2000 doses of the cGMP VSVΔG/ZEBOVGP vaccine so that we can proceed to pre-clinical and clinical trials. This is called the clinical trial material (CTM). At the end of the project a cGMP vaccine was generated by IDT Biologika and we received 1350 doses of CTM, as well as the master cell bank (MCB) and master virus bank (MVB) that were used to make the CTM (table 1). Obtaining the MCB and MVB allows for the future production of the vaccine using the same seed source that was used to make the CTM. During the manufacturing process a pilot study (10L test batch) was conducted to test the manufacturing process

prior to generating the CTM batch. It used the same processes as for the CTM but has not undergone the quality assurance testing that is performed on the CTM. We received this 10L test batch as one of our deliverables, which could then be used for further research. Part of the CTM stock will proceed into the clinical trials and the remainder will be stockpiled in case an emergency occurs before the vaccine comes to market.

Table 1: IDT Biologika Deliverables

Deliverable	Quantity
CTM	1350 doses
MCB	120 vials*
MVB	412 vials*
10 L test batch	1.2 Litres

* to be delivered when our facility is certified

The vaccine manufacturing process entails many steps, takes numerous days, and is formulated differently than the R&D vaccine that was initially tested in nonhuman primates (NHPs).Therefore the efficacy of the newly formulated vaccine had to be verified in animal models to assure that the manufacturing process did not affect the vaccine deleteriously. The CTM was tested in both mice and NHPs, and found to be relatively comparable to the research grade vaccine Figures 3 and 4.

Figure 3. *Efficacy testing of the CTM in mice*. Mice were vaccinated with varying doses (plaque forming units) of either the CTM or R&D VSVΔG/EBOVGP. The mice were then challenged 28 days later with a uniformly lethal dose (1000 LD_{50}) of mouse adapted ebola (MA-EBOV). The survival of the mice at the various doses is shown for the CTM and is compared to the R&D vaccine. The CTM is effective but less potent than the R&D vaccine.

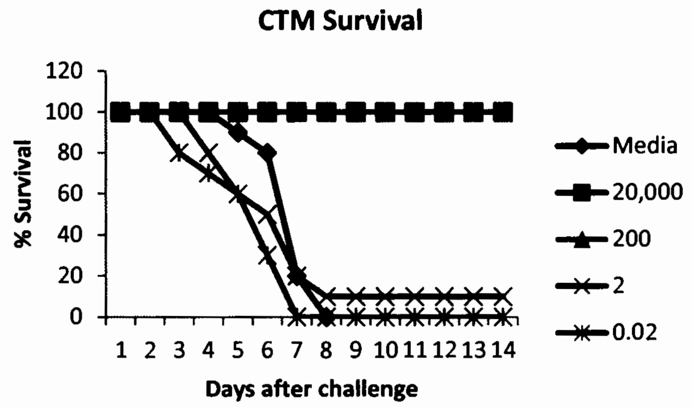

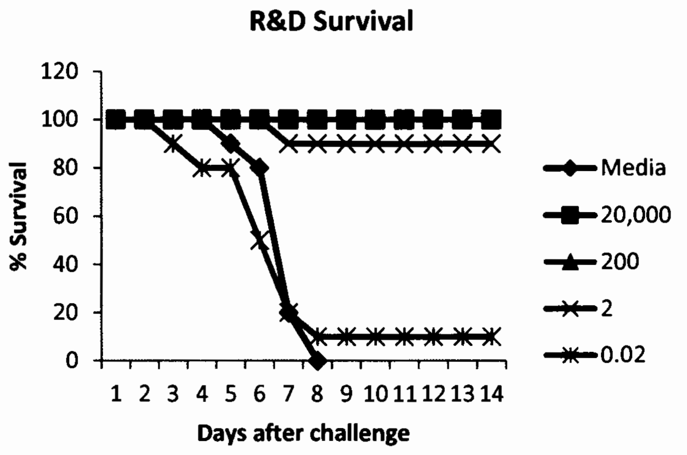

Figure 4. *Efficacy testing of the CTM in NHPs*. Cynomolgus macaques (n=4) were vaccinated with $1x10^8$ plaque forming units of the CTM VSVΔG/EBOVGP. The NHPs were then challenged 28 days later with a uniformly lethal dose (3000 $TCID_{50}$) of EBOV. The survival of the NHPs is shown for the CTM. The CTM fully protects the NHPs from a lethal EBOV infection.

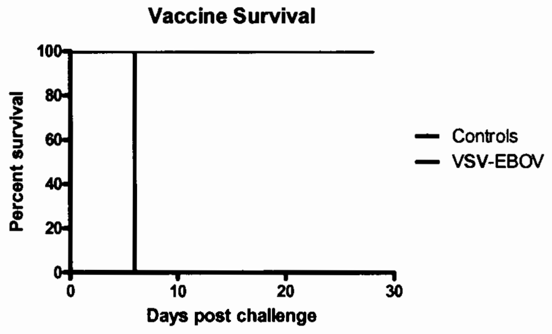

During the early stages of the VSVΔG/ZEBOVGP research it was demonstrated that the VSVΔG/ZEBOVGP could be used as a post-exposure therapeutic. Therefore the CTM was also efficacy tested in the mouse and NHP models (Figures 5 and 6).

Figure 5. *CTM efficacy testing of the post-exposure protocol in mice*. Mice were challenged with a uniformly lethal dose (1000 LD_{50}) of mouse adapted ebola (MA-EBOV). At 30 minutes or 24 hours after challenge the mice were treated with varying doses (plaque forming units) of either the CTM or R&D VSVΔG/EBOVGP. The mice were then followed for survival. The survival of the mice at the various doses is shown for the CTM and is compared to the R&D vaccine. The CTM is effective but less potent than the R&D vaccine.

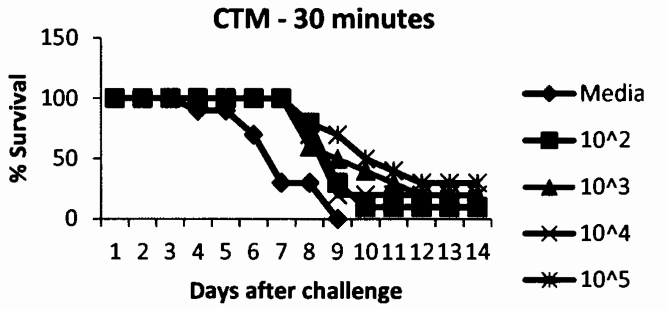
CTM - 30 minutes
% Survival
150
100
50
0
1 2 3 4 5 6 7 8 9 10 11 12 13 14
Days after challenge
Media
10^2
10^3
10^4
10^5

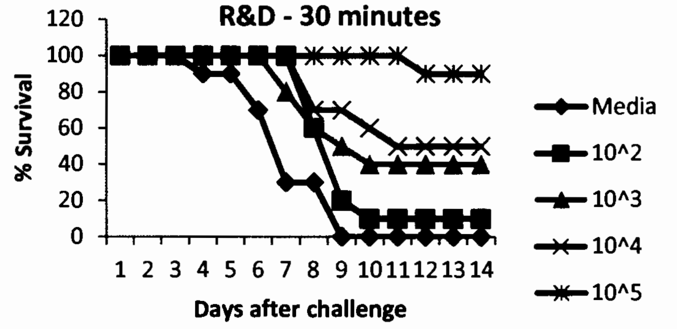
R&D - 30 minutes
% Survival
120
100
80
60
40
20
0
1 2 3 4 5 6 7 8 9 10 11 12 13 14
Days after challenge
Media
10^2
10^3
10^4
10^5

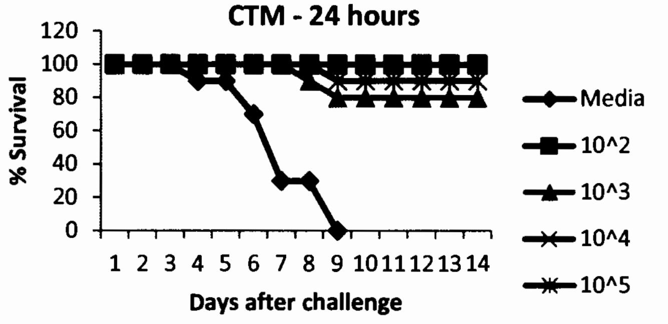
CTM - 24 hours
% Survival
120
100
80
60
40
20
0
1 2 3 4 5 6 7 8 9 10 11 12 13 14
Days after challenge
Media
10^2
10^3
10^4
10^5

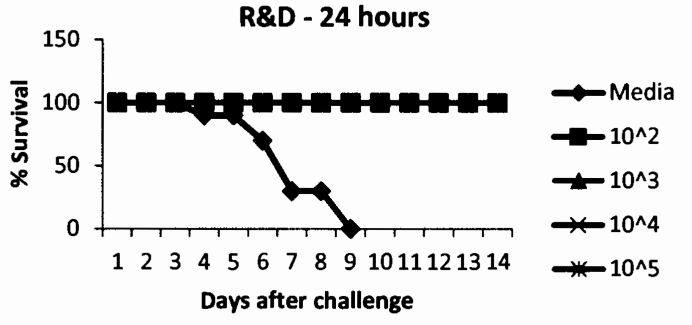
R&D - 24 hours
% Survival
150
100
50
0
1 2 3 4 5 6 7 8 9 10 11 12 13 14
Days after challenge
Media
10^2
10^3
10^4
10^5

Figure 6. CTM *efficacy testing of the post-exposure protocol in NHPs*. Rhesus macaques (n=6/group) were challenged with a uniformly lethal dose (3000 $TCID_{50}$) of EBOV. At 30 minutes or 24 hours after challenge the NHPs were treated with $2x10^8$ plaque forming units of the CTM VSVΔG/EBOVGP. The survival of the NHPs is shown. The controls (n=2) died on day 6. The 24 hour treatment group had no survivors with a mean time to death 7.7 days. The 30 minute treatment group protected 33% of the NHPs from a lethal EBOV infection.

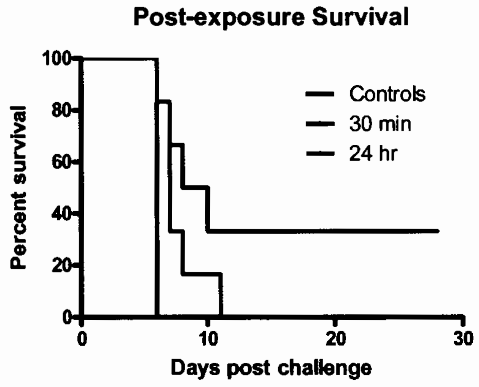

The second set of goals was focused on the research involving the understanding of the correlates of immune protection for an Ebola virus infection, and the development of treatment protocols and assays to support the application to Health Canada and the FDA for the vaccine to be used in humans. There were several accomplishments in this respect. We now have a post-exposure protocol developed in addition to the vaccine protocol (see above). In the vaccine protocol mice, guinea pigs and NHPs were immunized 28 days before a lethal challenge. We have also demonstrated that the route of vaccine delivery is equally effective via several different routes including intramuscular, intraperitoneally, orally, or nasally (Qiu 2009). However, we have also demonstrated using the murine model that you can immunize as late as 7 days before challenge and still get survival (2007 JID 106 (S2): S404). The post-exposure model was tested in mice and in NHPs. While the VSVΔG/ZEBOVGP was provided more survival when the mice were treated at 24 hours versus 30 minutes, the reverse was seen for the NHP study. It is not uncommon to see differences in treatment outcomes between different animal models. The NHP model is more representative of an Ebolavirus disease seen in humans and it is predicted that the effectiveness of the vaccine and post-exposure protocols will be similar to humans. In general, the disease onset is somewhat slower in humans therefore we expect to have more time for intervention.

Numerous immunological assays were developed to support the pre-clinical and clinical trials. These included the classical T and B cell assays as well as some new ones as follows.

B cell assays

The gold standard assay for B cell function is to quantify the viral-specific antibody in the sera of an immunized subject in an ELISA assay. An ELISA assay was utilized to measure the ZEBOV-GP-specific IgM (primary immune response), IgG (secondary immune response), and IgA (mucosal immune response) titres in NHPs immunized and subsequently challenged with VSVΔG/ZEBOVGP (Figure 7). The vaccine elicited a strong primary IgM response and mucosal IgA response. More importantly a robust IgG response was generated which is indicative of the rapid and longlasting B cell response.

Figure 7: ZEBOV-GP-specific ELISA for NHPs. Cynomolgus macaques were vaccinated either intramuscularly (IM), orally (OR), or intranasally (IN) with $2x10^7$ pfu VSVΔG/ZEBOVGP 28 days before challenge with 1000 pfu EBOV. Blood

samples were taken on the exam dates and the diluted sera added to an ZEBOV VLP ELISA.

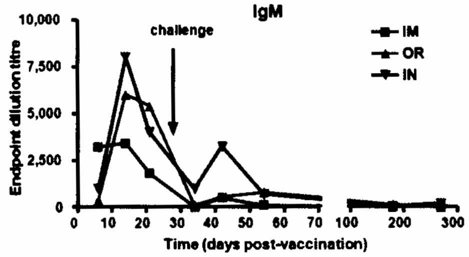

ZEBOV VLP ELISA

Serially diluted serum was added to plates coated with ZEBOV VLPs as the antigen.
Fold increase in pre- vs post-challenge peak titres

	IgA	IgG
IM	4.0	6.0
OR	3.9	22.4
IN	2.6	2.1

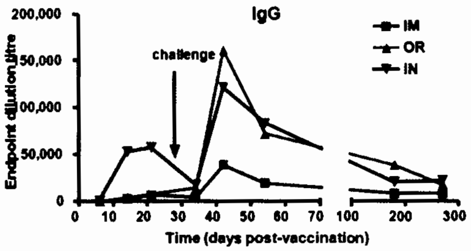

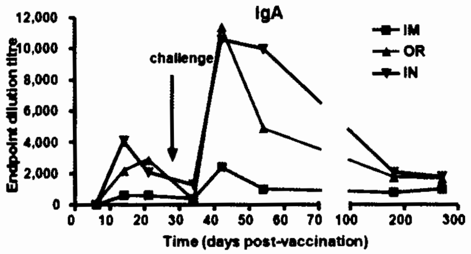

Neutralizing antibody titres have played significant roles in providing protection against viral and bacterial infections. This is most relevant in influenza virus infections where neutralizing antibodies are thought to provide the majority of protection. In natural Ebola virus infections neutralizing antibody titres are generally low or non-existent. However, numerous post-exposure therapeutics against Ebola virus utilized neutralizing monoclonal antibodies, with some successes and failures. It is not entirely clear what role neutralizing antibodies play in Ebola virus infections however, the ability of VSVΔG/ZEBOVGP to induce neutralizing IgG was examined. Two assays were utilized to determine the level of EBOVGP-specific neutralizing IgG in the sera of immunized subjects that can inhibit the infection of either Ebola or VSVΔG/EBOVGP. The gold standard plaque assay measured the ability of neutralizing antibodies to reduce the number of plaques that Ebola virus produces. This assay must be performed in a containment level 4 laboratory and the sensitivity of the assay is limited to 30-300 plaques. Therefore a new flow cytometry assay was developed that could be used in a level 2 laboratory. Instead of plaques as the readout, it examined the number of VSVΔG/ZEBOVGP infected cells by using a GFP-VSVΔG/ZEBOVGP virus. A reduction in GFP^{+} cells with the addition of immunized sera indicated the level of neutralizing antibodies in the immunized subject. Although the protocols for the two assays were somewhat different they comparable in their ability to detect neutralizing IgG, albeit the titres were low as expected. Since the flow cytometric assay could collect 10,000 events or more, this assay was more sensitive than the plaque assay, and also had a steeper dose-response curve and a broader linear range.

Figure 8: ZEBOV-GP-specific Neutralizing Antibody Assay. Sera from cynomolgus macaques was collected 21 days after being vaccinated either IM, OR or IN with VSVΔG/ZEBOVGP. Plaque assay: diluted sera was incubated for 1 hour with VSVΔG/ZEBOVGP before being added to Vero cells. The percent reduction in plaques indicated the neutralization capacity of the sera at each dilution. Flow cytometry assay: diluted sera was incubated for 1 hour with GFP-VSVΔG/ZEBOVGP before being added to Vero cells. After several days the cells were removed and made into a single cell suspension so they could be read on a flow cytometer. The percent reduction in GFP+ cells indicated the neutralization capacity of the sera at each dilution.

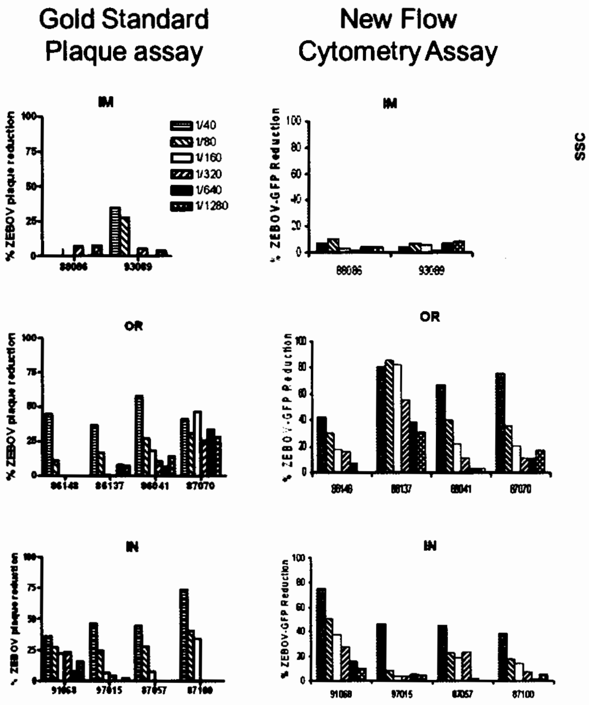

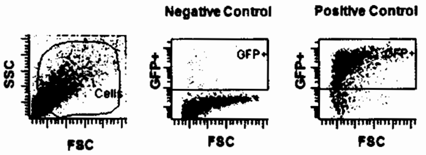

<u>Comparison of Assays</u>

- **Same NHP sera & dilutions were used**
- **different protocols reflect different assay requirements**
- **results of the two assays are comparable**
- **Flow based assay has a broader linear range, and a steeper dose response curve making it more sensitive to changes in antibody titre**

T cell assays

The cell mediated T cell response is the other protective branch of the immune system. The ELISPOT assay has been used for more than a decade to measure the virus-specific T cell response by quantitating the number of cells that produce cytokines in the presence of viral proteins. Commercial ELISPOT kits are available, and 7 different cytokines were examined to see if they were important measures of an Ebola virus specific T cell response. IFN-γ, IL-2, and TNF were all found to be elevated in mice. Therefore these cytokines were followed in the NHP studies. In the ELISPOT assay T cells secreted IFN-γ and IL-2 in response to Ebola GP peptides indicating a T cell mediated immune response(Figure #9). A flow cytometry assay (intracellular cytokine staining) was also utilized as you can obtain more detailed information about the cells that are secreting cytokines (Figure #10). VSVΔG/ZEBOVGP immunized NHPs CD4+ and CD8+ T cells produced cytokines IFN-γ, and TNF in response to Ebola virus peptides. So Ebola virus does induce a cell mediated immune response and it can be measured using the ELISPOT and intracellular cytokine staining assays.

Figure 9: NHP ELISPOT Assay. Peripheral blood mononuclear cells (PBMCs) from days 28 and 56 after IM vaccination with VSVΔG/ZEBOVGP were isolated and added to 1.5 ug/ml ZEBOV GP peptides on an IFN-γ or IL-2 ELISPOT plate. The number of cells producing either IFN-γ or IL-2 were quantitated using an ELISPOT reader.

Cytokine profile

- D28: IFN-γ > IL-2
- D56: IL-2 > IFN-γ

Immune Response to GP

- Breadth
  - Respond to all 3 pools
- Strength
  - pool 3 > 1 > 2

No. positive responders

- pool 3 > 1 > 2

Positive cutoff

- 2 x the average of the DMSO background wells from both day 28 and 56

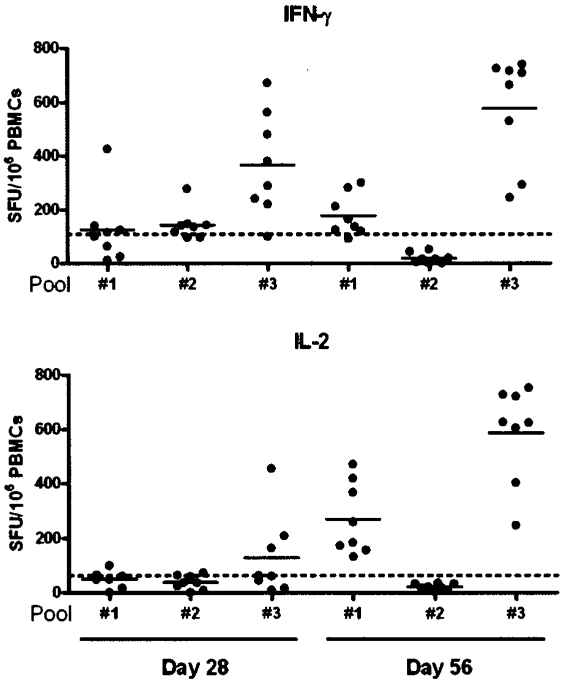

Figure 10: NHP intracellular Cytokine Assay: Peripheral blood mononuclear cells (PBMCs) from days 28 and 56 after IM vaccination with VSVΔG/ZEBOVGP were isolated and added to 1.5 ug/ml ZEBOV GP peptides and golgi plug. The cells were stained for the surface markers CD3, CD4, and CD8 before the intracellular staining for IFN-γ and TNF. The number of CD3+ CD4+ and CD3+CD8+ T cells producing either IFN-γ or TNF were quantitated using the flow cytometer.

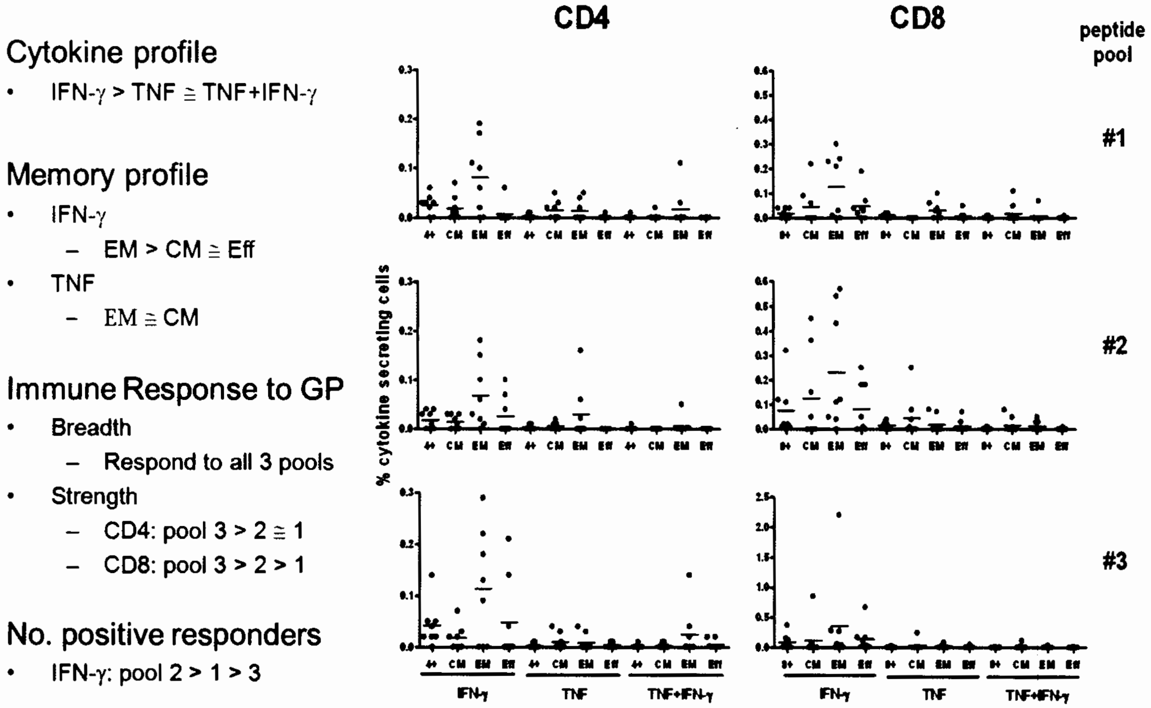

Determination of the correlates of immune protection for an Ebolavirus infection are important for designing vaccines and post-exposure therapeutics. Initially, it was believed that Ebola-GP-specific antibodies correlated with survival, however the effectiveness of antibodies was not definitive with some failures. Recent work with monoclonal antibodies are now demonstrating their ability to provide complete survival against infection. As such we were able to determine a minimal cutoff level of EBOV-GP-specific IgG antibodies needed for survival. Endpoint titres 28 days after immunization for survivors was 12,805 ± 29.905 versus 64 ± 59 for non-survivors.

Figure 11: ZEBOV GP-specific IgG responses in EBOV challenged NHPs. Diluted sera from cynomolgus macaques was collected 28 days after being vaccinated with VSVΔG/ZEBOVGP and added to a ZEBOV VLP ELISA. The NHPs were then challenged on day 28 with 1000 pfu ZEBOV. The graph demonstrates the ZEBOV GP-specific day 28 endpoint dilution serum titres of the NHPs that survived versus the non-survivors.

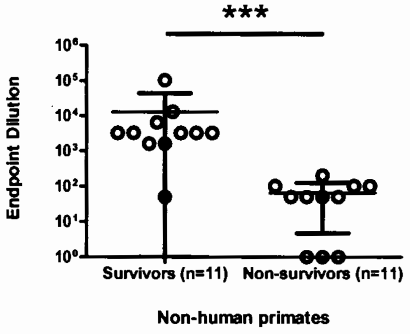

4. Impact on the Outcome(s)

Currently there are no approved vaccines or post-exposure therapeutics for an Ebolavirus infection. As such there remains a dire need for these products in the case of a bioterrorism event, accidental exposure, or natural outbreak. Prior to the start of the CSSP project we only had a limited R&D stock of the vaccine. Then in 2009, an Ebola virus researcher in Germany accidently stuck herself with a needle containing mouse adapted Ebola virus. The VSVΔG/ZEBOVGP was determined to be the best post-exposure treatment at that time and she was given 1 dose of the R&D vaccine 40 hours after exposure (2011 JID 204 (S3):S785). She survived without incident. Another incident involved a Dutch resident returning home ill after a trip to Uganda where she had toured a cave containing bats that are the natural reservoir for filoviruses. She was diagnosed with another filovirus infection, Marburgvirus (2009 Emerging Infectious Diseases 15(8):1171) and died 4 days later in a Dutch hospital. It was determined that there were 166 high risk and low risk contacts with the infected woman both in and out of the hospital. The PHAC had sent our other R&D VSVΔG/MARVGP vaccine to the Netherlands in case anyone wished to receive the vaccine as a post-exposure treatment. No one received the vaccine and fortunately no one else became ill. In 2009 there were 5 pig farmers who tested positive for Ebola virus. They had acquired the infection through the handling of pigs infected with Reston Ebola virus. Although Reston Ebola virus does not cause disease in humans, it was later determined that the highly pathogenic Zaire Ebola virus could infect pigs, who were then able to transmit the virus to NHPs within the same room (2012 Scientific Reports 2:811). The pigs did not succumb to the infection but this raised two serious issues. First, that pigs were susceptible to filovirus infections and as part of the food chain one infected pig could potentially infect many humans. Secondly, since the Ebola virus infection in pigs is primarily respiratory, they could easily transmit the virus to farm workers.

Aside from the naturally acquired infections mentioned above, there is still the threat of a weaponized Marburg virus or Ebola virus being unleashed. Overall, there is a great need to have a vaccine or post-exposure therapeutic available. In 2012 an International Filovirus Immunotherapeutic Consortium was formed. They ranked the VSVΔG/ZEBOVGP as the top candidate vaccine for the prevention of ZEBOV infections; and the ZMAb monoclonal antibody therapy was the top candidate for post-exposure therapy. This CSSP project has brought the VSVΔG/ZEBOVGP vaccine one step closer to being licensed for human use. We now have a cGMP CTM stock and part of it can be used for the pre-clinical and clinical studies. The remaining CTM is to be stockpiled in the case of any one of the emergencies listed above. It will be available until the licensure of the VSVΔG/ZEBOVGP vaccine in the next couple of years. The US company BioProtection Systems has licensed the vaccine from the Canadian Government and will then be responsible for the marketing of the vaccine. Already the groups expressing interest in obtaining the vaccine include all of the containment level 4 laboratories worldwide, and the Canadian and US military/governments. Having a cGMP vaccine available for Ebola virus will aid in the rapid delivery of the vaccine to first responders and affected individuals, in the case of a BT attack or an outbreak. This will assist in the emergency management/counterterrorism response to an Ebola virus bioterrorism event. It will also enhance public security and safety by minimizing the spread of Ebola virus within Canada and beyond the border.

5. Conclusions

The need for the Ebola virus vaccine is just as great now as it was when we were awarded the CSSP project in 2006. The Filovirus VSV platform has produced a very effective ZEBOV vaccine that can now proceed into clinical trials. There were some hiccups in the cGMP manufacturing process that resulted in delays, however now that the first production is complete any future manufacturing runs will proceed much more quickly. As part of the contract with IDT we have received the starting seed material, the MCB and MVB, needed for further productions.

The partners in the project have been invaluable. USAMRIID was involved in the very early stages of the project by conducting the initial NHP studies where they tested the VSVΔG/ZEBOVGP vaccine. Later on Health Canada provided their NHP expertise, along with animals from the NHP colony in Ottawa to assist PHAC in conducting NHP experiments at the NML containment level 4 laboratory. IDT's expertise in cGMP production of live vaccines was invaluable in obtaining CTM. BioProtection Systems is a US pharmaceutical company that has licensed the VSVΔG/ZEBOVGP vaccine from the Canadian Government. A strong collaborative effort between IDT, PHAC and BPS went into the manufacturing of VSVΔG/ZEBOVGP. Regular conference calls were conducted with all three parties to ensure that the proper cGMP protocols were being utilized to ensure licensure by the FDA and HC. PHAC was the subject matter expert on VSV and Ebola, IDT was the cGMP live vaccine manufacturing expert, and BPS had the cGMP and FDA experience. This entire project was truly a collaborative effort.

Much has been learned through this project. New technologies have been learned by the Special Pathogens staff. Many people have been trained in multi-colour flow cytometry, and on how to conduct a NHP experiment. Prior to this CSSP project the work in the Special Pathogens Program was primarily R&D, and diagnostic in nature. Now we have people in the building who have cGMP experience as a result of this project. Additionally, the NML has built a Biorepository which will be cGMP accredited so that it can store the CTM, MCB and MVB, as well as any other cGMP products the NML will be producing.

Although the CSSP funding is ending the project will go on. BPS is taking the CTM that we produced through the pre-clinical studies, and clinical trials. They will also be applying to the FDA for the VSVΔG/ZEBOVGP vaccine to be licensed for use in humans. In addition they will be working towards developing a pan filovirus vaccine that covers 3 Ebola virus species plus Marburgvirus. The results from the research conducted at PHAC will also provide the immunological assays that can be used in the trials, a vaccine and post-exposure treatment protocol, and advance the basic understanding of Ebola virus pathogenesis.

6. Recommendations

It is very fortunate that a private pharmaceutical company in the US has licensed the VSVΔG/ZEBOVGP vaccine from the Canadian Government. They will now take the vaccine through the pre-clinical toxicology studies and clinical trials. They will also apply to the FDA for licensure for human use, at which point it will be marketed after approval. Until then the unlicensed CTM will be available in the case of an emergency. The DND and Health Minister are aware of the vaccine. If any need arose the Public Health Agency of Canada would be contacted as with the previous incidences, and the need for receiving the VSVΔG/ZEBOVGP will be assessed on a case by case basis.

This CSSP covered the Zaire strain of Ebola virus which is the most pathogenic Ebolavirus with a 90% mortality rate. However, it will not protect against a Sudan (SUDV), Bundibugyo (BDBV) or Marbvirus virus (MARV) infection which has a 50%, 40% and 40-90% mortality rate, respectively. It is highly recommended that a pan filovirus vaccine be made to cover all of the most pathogenic filoviruses. The VSV platform is perfect for a pan vaccine scenario as several rVSVs can be made each with one of the glycoproteins from each of the filoviruses, and then various mixtures of the rVSVs can be made to suit the emergency. The US Department of Defence (DOD) is spending hundreds of millions of dollars to have a pan filovirus vaccine and post-exposure therapeutic available. PHAC already has the technology to make the pan filovirus vaccine but not the money to produce one. Without any political will in Canada to fund this work we will have to rely on funding from the US government.

In regards to CSSP, a Knowledge Translation mechanism should be implemented to ensure that the projects they fund have a way to make it to the end users. A list should be created for all of the innovations and their potential uses, then either posted on a website or provided as a document for potential users.

PROJECT PERFORMANCE

7. Technical Performance

From beginning to end the scope of the project never changed. One aspect of the project was to have 1000-2000 vials of the cGMP CTM VSVΔG/ZEBOVGP vaccine produced so that we could move forward with clinical trials and licensure. We achieved this goal with the receipt of 1350 vials of the CTM from IDT Biologika which is enough to proceed into clinical trials and still have some left over to stockpile in case of an emergency. In addition we received 120 vials of the MCB and 412 vials of the MVB. These two items are the seed stocks required to manufacture another identical cGMP batch in the future. Part of the manufacturing process involved a pilot run of the vaccine to verify all of the protocols are in place prior to manufacturing of the CTM. IDT gave us 1.2 L of this 10 L test batch that we can use for further research studies and for stockpiling. This test batch is made in a cGMP facility but has not undergone the same quality testing that the CTM undergoes to verify that it is safe for human use. Efficacy testing of the CTM was conducted by PHAC to confirm that the manufacturing process did not destroy the vaccines activity. A vaccine protocol and post-exposure protocol were tested in mice and NHPs. The CTM was approximately 1 log lower in effectiveness than the R&D vaccine. However, this lower potency was overcome by increasing the dose in NHPs by 1 log. The NHP results confirmed that the CTM was equivalent to the R&D vaccine protocol with 100% survival in all vaccinated animals. The post-exposure protocol saw 33% survival in the NHPs treated with the CTM 30 minutes after infecting them with ZEBOV. Previous research with the R&D vaccine saw 50% survival with the 30 minute treatment. Statistically, there is no difference between the two results. Overall, all vaccine manufacturing goals have been achieved as we have a large amount of an effective CTM.

The second part of the project required further research into Ebola virus infections plus the correlates of immune protection, and the development of assays in support of an application to the FDA. Two B cell assays (ELISA and Neutralizing Antibody Assay) and two T cell assays (ELISPOTS and Intracellular Cytokine Staining) were validated for the VSVΔG/ZEBOVGP vaccine using the NHP model. All four assays were able to detect ZEBOV GP-specific immune responses. These assays are being transferred to the US company that has licensed the vaccine, so they can be used for the pre-clinical toxicology trials, the clinical trials, and the two animal rule studies. The continuing research into Ebola virus infections has demonstrated a correlation between the ZEBOV GP-specific antibody response and survival. Overall, the research portion of the project achieved all of the original goals. An additional benefit achieved through this project was the training of many of the staff and students in the Special Pathogens Program on how to perform multicolour flow cytometry, particularly with murine and NHP samples. At the start of the project very few staff new how to perform a flow cytometry experiment with one or two colours. Now there are numerous people in the program who regularly conduct 6-12 colour flow cytometry. These technical advancements have benefitted the program immensely in conducting NHP experiments.

8. Schedule Performance

There was significant slippage in the schedule performance for this project. Much of this was due to delays with the vaccine manufacturer. Originally, this was a 5 year project but ended up closing several years past that. The confounding factors were as follows. It took 18 months just to work out the initial contract between the vaccine manufacturer IDT Biologika and Public Works and Government Services Canada (PWGSC). This delayed the start of the manufacturing process by a couple of years. Then there was difficulty in generating the initial viral seed at IDT Biologika. This was due to working within a cGMP atmosphere where we had to use a qualified cell line and not the cell line that we normally use to rescue the virus. IDT tried numerous methods to overcome this barrier but in the end we had to go back to using the original cell line. This meant that we had to add an additional purification step in the manufacturing process. There was a year spent rectifying the viral seed issue. Finally, the manufacturing of the cGMP clinical trial material failed twice resulting in a delay of another 18 months before the third and final batch was manufactured and released. Working with biologicals in a cGMP atmosphere can be challenging. Aside from it taking 18 months to generate a contract, the other delays were simply addressing the various cGMP challenges as they arose. Even though the project took much longer than expected the final product is well worth the effort.

9. Cost Performance*

The Task estimate and actual expenditures are summarized in the following table:

GL	Budgeted	Actual	Variation
Manufacture of Plasmids	$90,976.38	$122,056.14	+ $31,079.76
Manufacture of cGMP material	$680,757.00	$877,422.48	+ $196,665.48
NHPs (purchase/housing)	$34,433.00	$150,787.00	+ $116,354.00
**Total**			

*this table is only showing the larger items. For the full accounting for the project look at the annual financial reports that have been submitted.

10. Variance Analysis

There were several variances within the budget. The first is the contract with IDT Biologika to produce the cGMP vaccine. The original contract had a ceiling price of $680,757.00 CAD. By the end of the contract the ceiling price had risen to $877,422.48 CAD. The reason for the increase was due to additional work being performed by IDT. Two cell lines are required to rescue the viral seed stock but there is only one qualified cell line (WHO Vero cells) for human use. IDT was unable to rescue the initial virus seed stock using just the Vero Cell line. However, we wanted to maintain the cGMP status of the vaccine so IDT's contract was amended ($29,850.48) to test additional non-standard protocols to rescue the seed virus in Vero cells. When it became obvious that this approach would not work we decided to have the NML conduct the work in a non-GMP facility using the qualified WHO Vero cell line along with a non-qualified cell line. This necessitated IDT to perform a plaque purification process at a cost of $40,000.00 to eliminate any adventitious agents that may have been introduced during the non-GMP rescue protocol. Another step in the manufacturing process is the adventitious agent testing which assesses whether there are any harmful bacteria, fungi or viruses that have made it into the virus preparation. As VSVΔG/ZEBOVGP infection results in cytopathic effects in cells an antibody had to be supplied by PHAC to neutralize these effects in order to determine if there was another detrimental agent present. PHAC was unable to effectively neutralize the VSVΔG/ZEBOVGP therefore IDT had to proceed to a PCR based assay to screen for adventitious agents. This was contracted out by IDT and cost PHAC $76,350.00. The final additional costs ($50,465.00) arose with shipping and storage of the CTM, MCB, and MVB. The contract with IDT had not defined the FOB delivery definitively as being part of the contract, at which time IDT said shipping is never part of their contracts. Therefore shipping was added to the contract. Then in order to maintain cGMP status of all 3 deliverables, the NML built a cGMP Biorepository to store them. However there were numerous delays in obtaining the cGMP status so PHAC had to delay receipt of the deliverables resulting in storage costs. Overall, the additional expenditures with IDT, with the exception of the shipping costs, were not foreseen, nor avoidable. These additional costs were reasonable within the scope of a cGMP live vaccine production.

The contract with Plasmid Factory also saw an increase in costs. Part of the increase was due to the delay in being able to ship the plasmids to IDT Biologika, resulting in additional storage costs ($8,558.00). This was in part due to the delay in signing a contract with IDT which delayed the start of the project. The other part. \......

More was spent on NHPs than originally planned. In one case we had access to free NHPs which allowed us to perform some long term studies. However, we incurred some shipping and housing fees ($34,787.00) for that study. The second increase in money for NHPs arose from the final cGMP efficacy trials. Originally, only the vaccine protocol was going to be

tested. However, the US company BPS that licensed the vaccine wanted to take the post-exposure protocol to the FDA before the vaccine protocol. So efficacy testing was performed for both protocols resulting in an increase in costs to purchase and ship them ($81,567.00).

A decrease in hiring of additional personnel, and a decrease in general Ebola virus research was used to offset the increases in the budget mentioned above. The exact numbers are not known for this and are therefore not entered into the table above.

Another area of variance was the change in the total amount of project funding received from CSSP. Initially, any unused funds in a particular year could be rolled over into the next fiscal year. Therefore, the charter and annual budgets were drawn up to reflect this policy. However, later on CSSP stopped the rollovers resulting in a loss of ~10% ($195,000.00) for this project. This money had been reserved for the manufacturing and efficacy testing of the CTM but due to a production run failure this work was carried over into the next fiscal year. If PHAC had not supported this project and contributed another $250,000.00, the project would never have been completed. A decrease in the hiring of personnel in the CSSP project also meant that PHAC staff were pulled in to help on the project periodically, as needed. So some of the variance mentioned in the tables above were picked up by PHAC either in in kind funding or in kind contributions (i.e . personnel).

11. Deliverables – per the project Charter

Due to the significant delays in the project there were nine versions of the project charter with changes in dates for the deliverables in several charters. For the planned dates I am working off the earliest version that I have access to, version 6 dated December 15, 2006, when the project was approved and initiated. This will give us an idea what the original expectations were at the beginning of the project in comparison to what was actually achieved.

Deliverable	Planned Date	Actual Date	Notes
1000-2000 doses of the cGMP vaccine "clinical trial material"	June 2010	January 2014	We received 1350 doses of the clinical trial material. Part of this material will be used for the pre-clinical toxicology studies and clinical trials. The remainder will be stockpiled in case of an emergency.
Master Seed Virus Bank (MVB)	January 2009	September 2011 for the manufacture of the MVB and<br>July 2014 for delivery	Project Charter v6 only stipulated the production of the MVB (Jan 2009) as a preliminary step in the manufacturing process. So the actual equivalent date is September 2011. The MCB was released by IDT on July 31, 2013, meaning it was ready for delivery to PHAC then. Once our Biorepository is cGMP certified by Health Canada IDT will send us 412 vials of the MVB. The delay in receipt is due to PHAC not having the Biorepository ready on time. The Biorepository has to be cGMP certified in order to maintain the cGMP status of the MVB.
Master Cell Bank (MCB)	April 2008	January 2011 for the manufacture of the MCB and<br>July 2014 for delivery	Project Charter v6 only stipulated the production of the MCB (April 2008) as a preliminary step in the manufacturing process. So the actual equivalent date is January 2011. The MCB was released by IDT on April 31, 2013, meaning it was ready for delivery to PHAC then. Once our Biorepository is cGMP certified by Health Canada IDT will send us 120 vials of the MCB. The delay in receipt is due to PHAC not having the Biorepository ready on time. The Biorepository has to be cGMP certified in order to maintain the cGMP status of the MCB

Deliverable	Planned Date	Actual Date	Notes
10L test batch vaccine "pre-clinical stock"	August 2009	February 2012	PHAC received 1.2 L of the 10 L test batch. Originally, this was going to be used for the pre-clinical testing. However, BPS opted to perform the tests using the CTM material which would be better for the FDA application.
ELISA Assay	June 2007	June 2007	
Neutralizing Antibody Assay	November 2007	September 2011	
ELISPOT Assay	October 2008	September 2011	
Intracellular Cytokine Staining Flow Cytometry assay	June 2008	September 2011	
Correlates of immune protection	March 2011	March 2014	This is a never ending process as you can continue to discover new correlates as research proceeds. So in both charters the research into correlates continued until the end of the project.
Efficacy testing clinical trial material in mice and nonhuman primates	June 2011	December 2013	Testing of the CTM in mice and NHPs could not be conducted until the CTM was manufactured.

12. Evaluation of Project Success

Ebola virus infections are highly fatal (up to 90% mortality) and currently there are no approved vaccines or post-exposure therapies available for humans. Therefore the VSVΔG/ZEBOVGP vaccine is a substantial improvement over existing solutions which at present consists of supportive care. Having a cGMP vaccine that can be used for human infections will impact human disease directly by enabling a preventive and protective responsive capability of people and responders. In the event of a high risk scenario such as a natural outbreak or bioterrorism event the vaccine could be used to immunize infected patients family members, first responders, hospital staff, police and military personnel, or ring-immunize the community surrounding the affected area. This action would mitigate mortality and morbidity, and aid in casualty prevention and management. Limiting the impact of an ebola virus outbreak would maintain the integrity of the national economic and security infrastructures by reducing public fear and closures of hospitals or borders, and maintain a community work force thereby reducing any detrimental economic fallout.

The Canadian government has patented the vaccine, which has been licensed by a US pharmaceutical company who plans to apply for licensure in the US and Canada. So in the long term, when the vaccine is approved by Health Canada and the US FDA, it will be available worldwide to emergency responders, the military, international governments, and containment level 4 laboratories. Until then a small cGMP stockpile of the vaccine is available on a case by case basis for emergency situations. It is of value in terms of improving emergency management, and increasing operational effectiveness, risk reduction, and cost/loss reduction. In addition to mitigating human disease the vaccine can also be utilized to minimize animal ebola virus outbreaks. In the last couple of years it was determined that pigs can be infected with Ebola virus and can transmit the virus via aerosols. The impact of having Ebola virus in the food chain, as well as the ability to infect animal handlers increases the potential size of an outbreak. Therefore, if needed, this vaccine could also be used on animals. Although the probability of a severe outbreak is low, the catastrophic economic and personal impact has the potential to be quite high. Therefore, the value of this project was quite high considering the potential economic and health risk that exists. Thus, the vaccine fills an urgent requirement of the operational community (military and first responders) and will prevent dissemination of the viruses in the community providing safe and effective treatment at home and in the developing world.

FOLLOW-ON ACTIVITY

13. Project Transition

There is definitely life after the project. The Canadian Government has patented the VSVΔG/ZEBOVGP vaccine, and a US pharmaceutical company, namely BioProtection Systems, has licensed it. Now that we have a cGMP vaccine available BioProtection Systems will use part of the clinical trial material from the CSSP project to conduct the pre-clinical toxicology studies and the clinical trials. National Institutes of Health (NIH) will conduct the phase I and II clinical trials on behalf of BioProtection Systems with the phase I studies beginning in the fall of 2014, with the phase II clinical studies following after that. Phase III clinical trials will not be conducted as humans cannot be challenged with Ebola virus when testing the vaccine or post-exposure protocols. Therefore the two animal model rule will be implemented. BioProtection Systems will then apply to the FDA for the use of the vaccine in humans. Once approved by the FDA an application can be made for Canada as well. Once approved it is ready for market. I suspect all of this work will take several years to complete. During the approval process we will have part of the CTM VSVΔG/ZEBOVGP stock available for medical emergencies in the case of a bioterrorism event, a natural outbreak, or accidental infection.

Aside from supplying the cGMP clinical trial material for the above mentioned studies we are also transferring the immunological assays over to BioProtection Systems. This will include the T and B cell assay protocols, the NHP vaccine and post-exposure treatment protocols, as well as the viral and molecular protocols for generating other rVSVΔG/filovirus GP vaccines. PHAC will remain the subject matter experts for BioProtection Systems, and will most likely conduct any future containment level 4 experiments for the future vaccines that are generated.

14. Outstanding Activities

The project is essentially finished but there are just two items that need to be addressed. On the production side we will not receive the MCB and MVB from IDT until our Biorepository is cGMP certified. The MCB and MVB must remain in a GMP facility in order to maintain cGMP status. There have been delays in building and certifying the Biorepository at the NML so receipt of the MCB and MVB will not happen until July or August of 2014. As a result the contract with IDT will not end until then. However, all funds for the CSSP project have been used up so the funding for the final portion of the IDT contract will come from PHAC.

BPS will be responsible for the pre-clinical studies and clinical trials. Therefore PHAC is in the process of transferring all technical knowledge and reagents of the vaccine project to BPS so that they can proceed. Further collaboration between PHAC and BPS will be ongoing as PHAC will lend their VSV and Ebola virus expertise.

15. Disposition of Project Materials

Project files, documents, communication records. CSSP has received all of the relevant documentation.

Electronic copies of MS Project plans and documents. CSSP has received all of the relevant documentation.

LESSONS LEARNED

This project was very complicated and spanned a long period of time. Over the length of this project there were many changes in key personnel including 2 Project Champions with 1 Deputy Project Champion, 3 Project Managers, 3 Deputy Project Managers, and 2 CSSP Finance Officers. This resulted in a problem with continuity. Information was not passed on to the next person thereby creating some problems. The biggest issue that arose from this is determining what IDT was responsible for during the manufacturing of the VSVΔG/ZEBOVGP vaccine. The contract between IDT and Public

Works and Government Services Canada did not adequately define the work IDT was to perform. In most cases IDT did so much more work than what was defined, then on the other hand there was work they said they were not responsible for such as delivery of the CTM, that was not included in the contract. However, without the history of what was negotiated between the original Project Manager and IDT it became a constant stream of negotiations and amendments with IDT. A more complete Statement of Work should be used for future contracts. Also, 18 months to establish a contract seems excessive and any mechanisms that can be put in place to expedite this process should be utilized.

Budgeting the annual CSSP funds became an issue for this project. The original policy of being able to roll over unused funds into the next fiscal year was a Project Managers dream. In fact, the annual budgets were generated with this policy in mind. However, issues arose when the funds could no longer be rolled over, and at the same time there were delays in the vaccine manufacturing process. This resulted in losing $195,000.00 in CSSP funding that would have covered the remainder of the manufacturing process and the NHP efficacy studies. If PHAC had not funded the remainder of the project, this project would have failed in its final year, with no product to show for it. It is understandable that the rollovers create a financial difficulty for CSSP. However, it might be feasible to adopt a policy where critical funding can be rolled over versus holding back other money that would not affect the project detrimentally.

This project had a cGMP component which added on a level of complexity, and became a reason for some delays. GMP expertise is very limited at the NML. Therefore it was absolutely critical to make as many contacts with the regulatory people as possible. They provided invaluable advice in which direction to proceed. Most importantly was the ability to team up with pharmaceutical company BioProtection Systems who licensed the vaccine. Their GMP expertise was used extensively to assure the vaccine was manufactured to fulfill the FDA requirements. So it was beneficial to ask project team members and Portfolio Managers for their help in finding key experts.

This is one of the earlier CSSP projects that received funding, and as such received a substantial monetary award. This is no longer the case with projects now receiving smaller amounts of funding. As such it isn't feasible that another vaccine project such as this will happen again. However, it does not mean that it should not receive funding. In the US they are spending hundreds of millions of dollars to have pan filovirus vaccines and post-exposure treatments. Perhaps if the political will in Canada were to change in the future then the much larger sums of money could be earmarked for taking the vaccines or treatments all the way through the clinical trial phase. We were fortunate in this case that we had a company who licensed the vaccine and are willing to take through the remainder of the process. Otherwise once the CSSP project ended the vaccine may have just sat in the freezers. It would be best if when funding projects assurances are made that the work can be seen through to completion.

BPS DEVELOPMENT PLAN

ATIA - 20(1)(b)
ATIA - 20(1)(c)
Document Released Under the Access to Information Act / Document divulgué en vertu de la Loi sur L'accès à l'information
PHAC A-2014-00102

BioProtection Systems Corporation

VESICULAR STOMATITIS VIRUS (VSV)-BASED VACCINE PLATFORM DEVELOPMENT PLAN

Overview:

BioProtection Systems Corporation (BPSC) was incorporated in April of 2005 and is located in the Iowa State University Research Park in Ames, Iowa. BPSC develops novel antiviral vaccines using several State of the Art vaccine platform technologies. BPSC has a strong in-house research team comprised of virologists, immunologists, cell biologists and experts with extensive high-containment biosafety level 4 (BSL-4) facility experience, 60% of who are Ph.D.'s. One of BPSC's core technologies, the HyperAcute® αGal Technology, has been exclusively licensed from BPSC's parent company NewLink Genetics Corporation (NewLink).

NewLink was incorporated in 1999 and is also located in the Iowa State University Research Park. NewLink has four cancer vaccines employing the HyperAcute® Technology in phase I & II clinical trials that have shown no toxicity or adverse effects. BPSC and NewLink are jointly experienced with the development and protection of intellectual property, preparation of successful FDA Investigational New Drug applications (IND) and clinical trial design and performance. Further, we possess the comprehensive knowledge required for translational drug development.

During platform development, animal protection studies are performed as subcontracts in different BSL-4 facilities. Access to high containment facilities is crucial and BPSC has CRADA's and Sponsored Research Agreements in place with USAMRIID [redacted]

BPSC has established national and international collaborations with researchers at universities and government research facilities and is aggressively seeking to license promising patents to combine both existing and new vaccine platforms with the existing technologies.

BPSC is capitalized through several NIH/NIAID grants [redacted] a DoD SBIR grant as well as a multi-million $ DTRA contract in addition to private investors. BPSC and its developmental partners have the capability and capacity to manufacture, to bring drugs to the clinic and to follow the development of a drug through to FDA approval. cGMP facilities are available with established QA and QC processes. NewLink has a regulatory team is in place as well as a clinical trial management team interacting with clinical coordinators.

2901 SOUTH LOOP DRIVE, SUITE 3360 AMES IOWA 50010-8646
PHONE (515) 296-2592

Document Released Under the Access to Information Act / Document divulgué en vertu de la Loi sur L'accès à l'information

ATIA - 20(1)(b)
ATIA - 20(1)(c)

Capabilities and Capacity:

- Experienced research team capable to design and conduct required scientific tasks
- Established infrastructure (i.e., laboratory and administrative space)
- Adequately capitalized for planned research projects
- State-of-the-Art facilities (i.e., cGMP) available
- Experienced in cGMP manufacturing, drug testing, follow-up clinical trials through NewLink
- Dedicated regulatory teams, Quality Assurance (QA) and Quality Control (QC)
- Industrial partner with designed path for successful product introduction into the market
- Active government affairs division whose sole responsibility is to establish partnerships and development opportunities [redacted]

Competitive Advantages:

- [redacted]

Corporate Strategy:

- [redacted]

2901 SOUTH LOOP DRIVE, SUITE 3360, AMES, IOWA 50010-8646
PHONE (515) 296-5592

Document Released Under the *Access to Information Act* / Document divulgué en vertu de la *Loi sur L'accès à l'information*

ATIA - 20(1)(b)
ATIA - 20(1)(c)

Potential Products:

HyperAcute® αGal Technology:

[redacted]

2901 SOUTH LOOP DRIVE, SUITE 3360, AMES IOWA 50010-8646
PHONE (515) 296 3592

Document Released Under the *Access to Information Act* / Document divulgué en ver de la *Loi sur L'accès à l'information*

WITHHELD

Is(Are) exempted pursuant to sections(s)
est(sont) exemptée(s) en vertu de(s)(l')article(s)

20(1)(b)
(b) financial, commercial, scientific or technical information that is confidential information supplied to a government institution by a third party and is treated consistently in a confidential manner by the third party;
(b) des renseignements financiers, commerciaux, scientifiques ou techniques fournis à une institution fédéra par un tiers, qui sont de nature confidentielle et qui sont traités comme tels de façon constante par ce tiers;

20(1)(c)
(c) information the disclosure of which could reasonably be expected to result in material financial loss or gai to, or could reasonably be expected to prejudice the competitive position of, a third party; or
(c) des renseignements dont la divulgation risquerait vraisemblablement de causer des pertes ou profits financiers appréciables à un tiers ou de nuire à sa compétitivité;

Document Released Under the Access to Information Act / Document divulgué en vertu de la Loi sur L'accès à l'information

ATIA - 20(1)(b)
ATIA - 20(1)(c)

[redacted]

Focus:
BPSC focuses on the development of vaccines against high priority agents as defined by NIAID and emerging viral pathogens that result in deadly hemorrhagic fevers. The HyperAcute® αGal Technology has been applied to select vaccine platforms including VLPs, inactivated viruses and pseudotyped viruses that are currently undergoing efficacy tests via live virus challenge studies in mice and rats with our collaborators in several BSL-4 facilities. [redacted]

We believe our product development strategy will accelerate potential commercialization of your platform. We anticipate taking this product to clinical trials as early as 2011.

We are collaborating with world-renowned scientists in the field of VSV-based vaccines. [redacted]

Document Released Under the Access to Information Act / Document divulgué en vertu de la Loi sur L'accès à l'information
PHAC A-2014-00102
ATIA - 20(1)(b)
ATIA - 20(1)(c)

Markets:

According to a study conducted by the Freedonia Group, "Vaccines to 2008 – Market Size, Market Share, Market Leaders, Demand Forecast and Sales", published in August of 2004, the demand for [redacted]

The study data was collected from a variety of primary and secondary sources, including published data, industry participants, online databases and other Freedonia studies. Primary information was obtained through consultation with officers and marketing/technical personnel of participating companies. Secondary and background data sources consisted of various government, trade and industry publications such as BioPharm, Chemical & Engineering News, Chemical Market Reporter, Chemical Week, Drug Delivery Technology, Drug Discovery & Development, Drug Topics, Genetic Engineering News and R&D Directions. Reports from the US Food and Drug Administration and Pharmaceutical Research and Manufacturers of America were also consulted, as were corporate annual reports, SEC filings, product literature and other company information.

Viral hemorrhagic fever viruses are perfect examples of emerging and reemerging pathogens. Infections are serious public health concerns not just in developing countries where these viruses are endemic but also in many developed countries. Some of them are listed on the category A list of bioterrorism agents (Centers for Disease Control, 2003, http://www.bt.cdc.gov/Agent/Agentlist.asp) and thus represent a threat to the world's population. Studies on many of these pathogens, such as Lassa virus, Marburg virus, and Ebola virus, have been impeded in the past by the biocontainment needed for their manipulation, biosafety level 4 (BSL4). Although these viruses can be grown in tissue culture, virus propagation is comparatively slow, and titers are generally lower than those of other viral pathogens.

Lassa fever is one of the hemorrhagic diseases, a class that includes the dreaded Marburg and Ebola viruses, and is considered a prime agent for bioterrorism. In recent years, several travelers have brought the disease with them to the United States and Europe. We believe there will be great interest from various international governmental agencies, as well other organizations such as Gates Foundation, to invest in this life saving product.

[redacted]

Document Released Under the Access to Information Act / Document divulgué en vertu de la Loi sur L'accès à l'information

clinical trials in a classical phase I and II approach will be performed followed by a final phase III study thus facilitating entry into the commercial vaccine market.

Summary Work plan:

BPSC will strengthen the available research, efficacy and safety data with additional required studies to rapidly prepare and execute an IND and move the replication-competent VSV vaccine platform into phase I/II clinical trials.

Objective #1: Preclinical vaccine testing

We have the experienced regulatory, QA, QC and preclinical testing team efforts in place to complete all guarantee performance that meets the rigorous studies required by requirements of the FDA to submit for approval of an IND. Our team will work closely with PHAC's Special Pathogens program and combine their experience and expertise with ours.

Objective #2: Safety and toxicology studies

Our in-house experience and relationships with several CRO organizations that conduct pre-clinical testing will enable us to perform any additional studies that may be required by the FDA prior to the initiation of clinical trials with the envisioned rVSV–based vaccines.

Objective #3: Manufacturing of the vaccine

We have the capacity and capability to manufacture the vaccine candidates under cGMP conditions at sufficient scale to allow completion of all preclinical and clinical testing. Furthermore, our well trained staff continues process development research to develop additional SOP's to monitor and improve production methods to optimize efficiency of vaccine manufacturing.

Objective #4: Interactions with FDA and Clinical Testing

We have the required teams and infrastructure (regulatory, medical, QA, QC) in place to complete this objective. In addition, we are in the process of implementing an electronic data capture system that is capable of highly efficient acquisition and management of all clinical data in a highly efficient manner. We believe the ability to perform these functions internally adds value to our company and should significantly accelerate product development and commercialization of this promising technology.

2901 SOUTH LOOP DRIVE SUITE 3360, AMES, IOWA 50010-8646
PHONE (515) 296-3592

Document Released Under the Access to Information Act / Document divulgué en vertu de la Loi sur L'accès à l'information

ATIA - 20(1)(b)
ATIA - 20(1)(c)

Detailed work plan:

Design and generation of vaccine candidates and initial efficacy studies have already been performed by PHAC's Special Pathogens program. Together with the FDA, we will determine and implement any additional studies that are required.

cGMP manufacturing: compounding of active pharmaceutical ingredients:

BPSC will be responsible for the compounding of the active pharmaceutical ingredients and will use its internal Quality Management program to assure compliance with all applicable FDA guidelines and regulations. [redacted] The Quality Control unit performs multiple quality control tests on in-process and finished product samples. [redacted]

Quality Assurance (QA) and Quality Control (QC):

The QA unit maintains compliance with applicable regulatory guidelines for the manufacture and testing of pharmaceutical products. The QA monitoring function includes the establishment of precise specifications for starting materials, equipment, facilities, and personnel in addition to approving standard operating procedures in the manufacture and testing of products. The QA unit is also responsible for maintaining all documentation of process control and for testing programs that measure stability and equivalence of product lots. [redacted] BPSC will provide quality management to consist of oversight in two phases of product development: pre-clinical and Phase I clinical.

Quality control will similarly have two parallel programs: (i) compounding of the active pharmaceutical ingredients and (ii) the codification of the specifications, the development of manufacturing protocols that will reproducibly generate such materials, and testing protocols to assure adherence of the manufactured materials to the IND specifications. Quality control testing of final product vaccine will be comprised of multiple techniques. Standard release testing will include sterility, endotoxin, pyrogenicity, and general safety [redacted]
A quarterly stability testing program that repeats the product specific testing, in addition to sterility, is anticipated for a minimum of 36 months.

Regulatory and Clinical Affairs:

BPSC's medical team comprised relationship with NewLink Genetics includes full access to the services of MD's, the physicians, registered nurses and Clinical Research Associates (CRA) have recently demonstrated high level competency with extensive expertise in clinical trials trial management and regulatory affairs.

Document Released Under the Access to Information Act / Document divulgué en vertu de la Loi sur L'accès à l'information

ATIA - 20(1)(b)
ATIA - 20(1)(c)

Safety and Toxicology studies:

[redacted] in regards to this specific vaccine candidate, BPSC will work with PHAC's Special Pathogens Program team who has access to BSL-4 facilities that will enable the completion of the safety and toxicology studies. Furthermore, our relationships with several CRO organizations that conduct pre-clinical testing will enable us to perform any additional studies that may be required by the FDA prior to the initiation of clinical trials with the envisioned VSV–based vaccines.

IND and Phase I/II clinical trials:

The current status of basic research with the rVSV-based filovirus vaccine strongly suggests that clinical development of the product will proceed quickly. The FDA is currently aware of the project goal of producing the rVSV-based vaccine and it is anticipated that an initial pre-IND will be conducted shortly to confirm the design of the pre-clinical safety and toxicology testing program. Completion of the pre-clinical research plan will allow the preparation of an IND covering the vaccine clinical studies. [redacted] The conclusion of process development work, including stability studies and preparation of demonstration lots, will enable the production of the clinical lots to final specifications in time for the Phase I studies.

[redacted] The anticipated Phase I trial will have the primary objective of evaluating the safety of the filovirus vaccine(s) by examining the effects of a single dose of rVSVΔG/MARVgp and rVSVΔG/EBOVgp vaccine. The secondary objective will be the determination of the immune response against target organisms following administration of vaccine as measured by various standard laboratory assays.

2901 SOUTH LOOP DRIVE, SUITE 3360 AMES IOWA 50010-8646
PHONE (515) 296-3592

PHAC LICENSE NEGOTIATION TERM SHEET

Document Released Under the Access to Information Act / Document divulgué en vertu de la Loi sur L'accès à l'information

Public Health Agency of Canada | Agence de santé publique du Canada

License Negotiation Term Sheet

NOTE: THIS IS NOT A LICENSE OR PERMISSION TO USE THE PHAC TECHNOLOGY AND IS NOT A LEGALLY BINDING DOCUMENT

**Licensor**	Her Majesty the Queen in Right of Canada as represented by the Minister of Health through the Public Health Agency of Canada (PHAC)<br><br>1015 Arlington Street<br>Winnipeg, MB R3E 3R2 CANADA
**Licensee**	BioProtection Systems Corporation<br>Iowa State University Research Park<br>2901 South Loop Drive, Suite 3360<br>Ames, Iowa 50010
**Licensee Details:**<br>Type of Entity<br>Where organized<br>Fiscal Year	 __________<br>__________<br>__________
**Type of License**	Patent
**Subject Matter to be Licensed**	Recombinant vesicular stomatitis virus vaccines for viral hemorrhagic fevers, including Patent Rights described in Attachment A. Patent Rights includes any issued, unexpired, valid claim or pending claim contained in the Patent Rights issued within seven (7) years of the filing date of the patent application from which it arose.<br>US Patent Application #10/522,134<br>Canadian Patent Application # 2,493,142<br>European Patent Application 03 771 017.5
**Grant**	(a) To make, have made, use, lease and sell licensed products, (b) to practice licensed processes and (c) to use the technology, directly or by sublicense for commercial purposes in the Field of Use within the Territory during the Term.
**Exclusivity**	Sole license with respect to patents<br><br>PHAC to retain non-commercial rights, use for educational and research purposes reserved.<br><br>PHAC to retain rights to emergency use prior to product approval.<br><br>PHAC to retain rights to emergency use for its staff post-approval.
**Right to Sublicense**	Included, provided sublicensee agrees to abide by certain provisions of the License – including, provisions requiring BPS to flow down the terms of the license to sub-licensees in order to protect the interest of PHAC.<br><br>Consideration for a sublicense shall not include anything of value in lieu of cash payments without permission of PHAC.<br><br>Sharing of income from sublicenses and the effect of termination of the license on sublicenses are provided for below.

Document Released Under the Access to Information Act / Document divulgué en vertu de la Loi sur L'accès à l'information

ATIA - 18(a)
ATIA - 20(1)(a)
ATIA - 20(1)(b)

Public Health Agency of Canada · Agence de santé publique du Canada

**Field of Use**	Prophylactic use for VHF viruses.
**Territory**	Worldwide.
**Term**	Longer of (a) expiration of last to expire claim included in licensed patents, and (b) ten years from first commercial sale.
**Due Diligence**	Commercially reasonable efforts to develop and market Licensed Products. Specific Milestones, due dates and associated payments are provided for below.<br><br>A "no shelving" clause in the license agreement to ensure that BPS does not sit on the technology.<br><br>Annual report on progress towards introduction of licensed products.<br><br>If license is terminated, licensee to return improvements and information relevant to commercialization to the PHAC.

**Milestones**	**Date** (mm/dd/yyyy)	**Payment**
Submit IND/IDE/510(k) Application	__/__/____	[redacted]
Completion of Phase I Clinical Study	__/__/____	
Completion of Phase II Clinical Study	__/__/____	
Submit NDA	__/__/____	

**Financial Terms**	
**Responsibility for Patent Costs:**	Licensee will reimburse Prior Patent Costs, which amounted to $ ______ as of _____ within 30 days of Effective Date of License.
**Initial Payment:**	[redacted] non-credible and non-refundable
**Minimum Royalty Amount:**	[redacted] Payable January 1 of the year shown and creditable against earned royalties or sublicense payments in the same year.
**Royalty Percentage**	[redacted] of Gross Sales by Licensee
**Third Party Licenses**	Licensee may enter into a license with a third party if necessary in order to commercialize the licensed rights. If the existence of the third party patent could not have been known as of the Effective date of the license, half of such third party payments can be credited against up to half of the running royalties in any calendar quarter.
**Lump Sum Percentage:**	[redacted] of all lump sums received from sublicenses, excluding research support payments (next section). Includes proceeds from purchases of equity in Licensee which are associated with a partnership which includes a sublicense to the extent that per share price exceeds [redacted] of current market value.
**Research Support Percentage:**	Good faith effort to collaborate with PHAC for [redacted] of basic research and further development under separate agreements.
**Earned Royalties**	[redacted] of gross royalties received from sublicensees.
**Reports**	Quarterly reports of sales, royalties, milestone events and sublicenses; payment to be received within 45 days of end of quarter.<br><br>Annual copy of certified financial statements and evidence of renewal

Document Released Under the Access to Information Act / Document divulgué en vertu de la Loi sur L'accès à l'information
PHAC A-2014-00102

	of insurance within 90 days of end of fiscal year.<br><br>Annual reports to stockholders and material revisions to business plan when prepared.
**Audit**	At PHAC's expense. Immediate payment of arrears. Overpayments credited against next payment due. If underpayment of 5% or more discovered, Licensee to pay costs of audit.
**Patent Filings**	By agents/attorneys selected by PHAC. Licensee has right to comment on proposed actions and filings.
**Patent Costs**	Licensee to reimburse PHAC for prior patent costs on the Effective Date, and for subsequent patent costs on a monthly basis within 30 days or receipt of invoices.
**PHAC Limitation**	PHAC makes no representations or warranties on the patent rights.
**Indemnification**	Licensee to hold PHAC harmless from claims and suits arising out of Licensee's use and commercialization of the licensed technology, including those arising from Licensee's negligence.
**Insurance**	Licensee to procure and maintain in full force and effect commercial general liability insurance policies that protect and name the Indemnitees as additional insureds. Coverage shall be no less than $5,000,000 per incident and $5,000,000 in annual aggregate.<br><br>Licensee shall provide the PHAC with written evidence of such insurance upon issuance and upon each annual renewal. Licensee shall give the PHAC at least thirty (30) days written notice prior to any cancellation, non-renewal or material change in such insurance.
**PHAC Liability**	For any cause of action to be capped/limited to royalties and payments received by PHAC.
**Prosecution of Infringers**	Licensee to have first right to sue infringers. Any recoveries go first to reimburse expenses then 75% to Licensee and 25% to PHAC.<br><br>If Licensee does not take action to prevent infringement within one (1) month of becoming aware of infringement, PHAC has right, but not the obligation, to sue. PHAC retains all recoveries.
**Termination**	Unless terminated earlier for breach or convenience, the license for Patent-Based products and processes will continue in each country until the expiration or termination of Patent Rights in said country.
**Early Termination**	In case of a material Breach or failure to pay royalties, PHAC may terminate the license upon thirty (30) days prior written notice. Licensee can cure the breach within this period to maintain the license.<br><br>In case of insolvency or bankruptcy of licensee, the license is terminated immediately.

The parties will pursue negotiations in good faith between December 01, 2007 and March 31, 2008, in an attempt to implement the transaction described above.

Neither party shall be legally bound to this proposed transaction (whether expressed orally, in writing, or by implication). The parties shall be bound only by a final written document executed on their behalf by their duly authorized representatives. This term sheet is not such a document. It is merely a reflection of the parties' intent to negotiate such a document and is not binding at law.

Document Released Under the Access to Information Act / Document divulgué en vertu de la Loi sur L'accès à l'information

Public Health Agency of Canada / Agence de santé publique du Canada

It is anticipated that the negotiations will involve the usual mix of discussion, letters, and draft agreements, but, prior to the due execution and delivery of a final document, the parties shall have no binding agreement between them and must not rely on written, oral, or implied promises that may be given during the course of the negotiations.

The only commitments made by PHAC during these negotiations are to: 1) not to enter into negotiations with other parties to license the Patent Rights; and 2) pay the costs of patent prosecution incurred by the PHAC during the negotiation period.

**Her Majesty the Queen in Right of Canada as represented by the Minister of Health through the Public Health Agency of Canada**	**BioProtection Systems Corporation**
Name	Name
Date:	Date:

Document Released Under the Access to Information Act / Document divulgué en vertu de la Loi sur L'accès à l'information
PHAC A-2014-00102

ATTACHMENT A

Patent List

	Title	Inventors	Type	Country	Filing Date/ Issue date	Application No./ Patent No.	Status	Funding

PROJECT UPDATES
A

SCHEDULE / MILESTONE REVIEW

Initiation of Project

Milestone	Actual or Forecast date	Notes
Project Approval	15 Dec 06	Completed
Project Implementation Begins	18 Dec 06	Completed
Contract Award	12 Jan 07	Completed
Contracts with partners signed (Plasmid Factory and IDT)	30 Sep 08	Completed

SCHEDULE / MILESTONE REVIEW I

Milestone	Actual or Forecast date	Notes
Develop standardized ELISA for assessment of murine and NHP antibody responses	30 June 07	Completed
Develop ELISPOT assay for the characterization of murine T-Cell responses	30 Sept 10	Completed
Development of ELISPOT and flow cytometry assays for NHP responses to Ebola vaccine	30 Sept 11	Completed
Develop flow cytometry assays for measuring proliferation and functional activity of murine T-cells	30 Sept11	Completed
Develop standardized assay for antibody neutralization for murine and NHP antibody responses	30 Sept 11	Completed NHP<br>Mouse not needed
Immune correlates of protection data set completed	31 May 13	Ongoing - add to dataset as time and funds permit

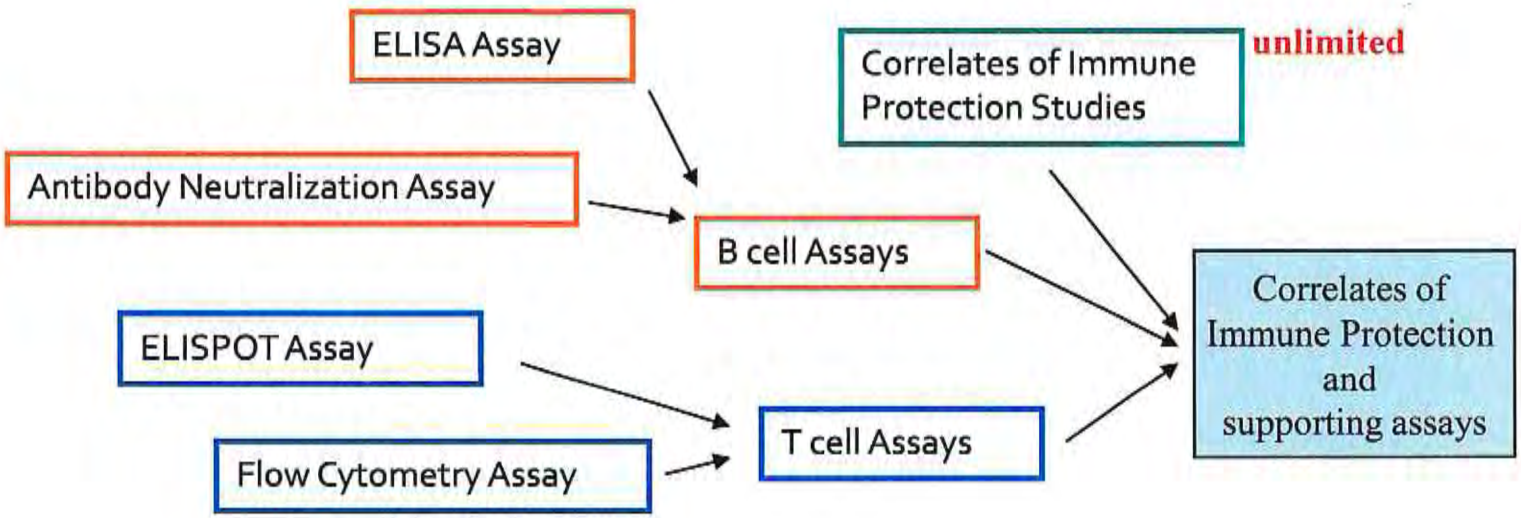

SCHEDULE / MILESTONE REVIEW

Milestone	Actual or Forecast date	Notes
Rescue vaccine seed stock and delivery to IDT	10 Jan 11	Completed
Preparation of cells for vaccine production (MCB)	21 Jan 11	Completed
Plaque Purification	31 Mar 2011	Completed
Preparation of Master Seed Virus Bank (MVB)	23 Sep 11	Completed
Manufacture 10L test batch of VSVΔG/ZEBOVGP	28 Feb 12	Completed

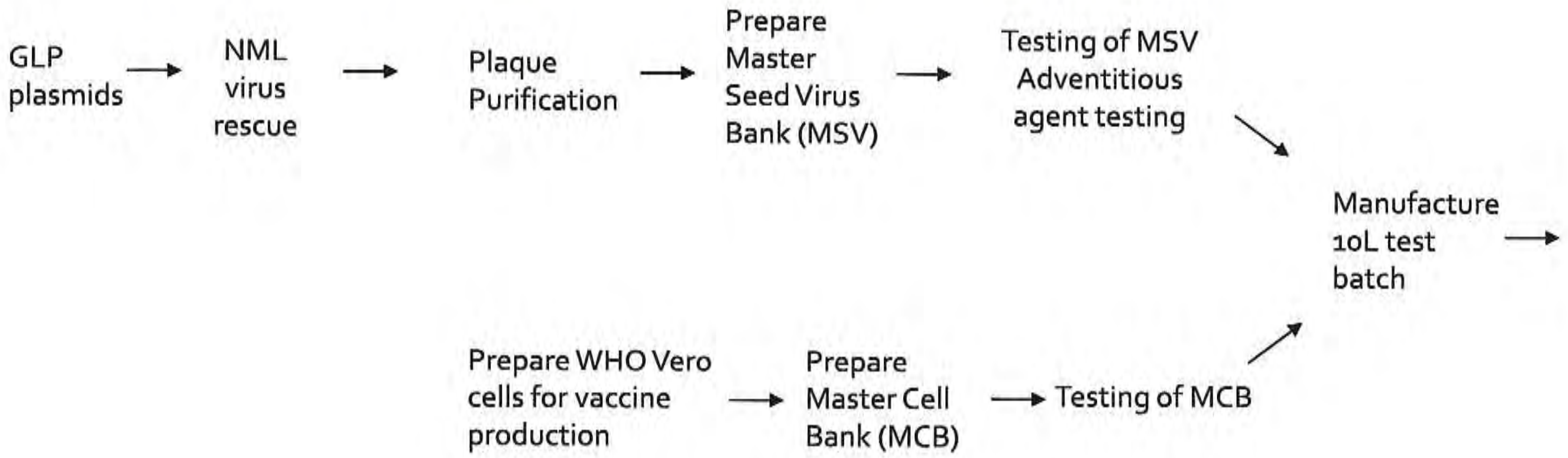

SCHEDULE / MILESTONE REVIEW

**Milestone**	Actual or Forecast date	Notes
Interim report on efficacy of test batch material in mice	31 Mar 12	Completed
Manufacture clinical trial material	30 Apr 12	Currently being manufactured
Delivery of cGMP material	31 Dec 12	Not Started<br>- Delivery in March 2013
Completion of cGMP VSV testing in mice and NHP	31 May 13	Not Started
Submission of final Report	30 June 13	
Project Complete	30 June 13	

→ Manufacture 10L test batch → Vaccine Efficacy Testing - Mice - - Guinea Pigs- → Manufacture cGMP clinical trial vaccine → Vaccine Efficacy Testing - mice - - NHPs - → 1000-2000 units cGMP VSVΔG/ZEBOVGP

PROJECT UPDATES
B

Topics for the meeting with [redacted] and [redacted] BPS on Tuesday May 18, 2011

Final formulation

- I informed BPS
    - o IDT has said that the residual Vero DNA in the clarified prep is too high therefore there will be a purification step
    - o IDT will be performing some purification trials including depth filtration and Benzonase treatment with consecutive diafiltration approaches
    - o IDT also recommends to formulate the final product in a stabilizer based on amino acid/ carbohydrate formulation. It is rather risky to store rhabdoviruses without a stabilizer which prevents formation of crystals during freezing. The recommend stabilizer could facilitate freeze-drying at a later stage. Another approach could be the use of 50% buffer glycerol solution. But I would not recommend that.
- I told them that I was in the process of arranging a meeting with IDT, BPS and me so that we can discuss the formulation and any other manufacturing questions that they have. I would like to have a meeting within the next two weeks.

Adventitious agent testing

- this includes: adventitious agents testing of MSV(Adventitious agents testing is restricted to the following tests: in vivo (suckling and adult mice, guinea pigs), in vitro, retroviruses and other transforming viral agents (qPERT), bacteria and fungi, mycoplasma, mycobacteria, and other specific viral agents (if applicable- bovine, porcine, human and simian).)
- I told them that IDT recommends you get a statement from the FDA on the necessary adventitious agent testing, especially regarding the non-GLP step in the manufacture process.
- BPS said they will gently bring this up to the FDA

Assay development

- We talked about assay development and what would be the most appropriate assay to put forward as the correlates of immune protection. The difficulty arises in determining what the mechanism of protection is for the post-exposure and then proving that this protective mechanism is what is induced in humans. We are going to think about what is the best approach to put forward to the FDA. We are thinking that IFN is very important….so we might do a cytokine screen. However, there was also the confirmation that IFN is only responsible for 33% of the survival. Also, there is the fact that there is some specificity in the response as VSV MARB does not provide the same level of protection as VSV-ZEBOV.
- Should develop the assays that would be beneficial for providing the correlates of immune protection for the post-exposure treatment. It would be beneficial to determine what the best assays are. These assays are different than what we would use for the vaccine.
- Currently have the following assays
    - o IL2 and IFNγ ELISPOT
    - o Flow NAb assay

- VLP ZEBOV GP specific ELISA
- Flow cytometery ICS, proliferation, and immunophenotyping

Sequencing.

- We will be getting the MSV stock from IDT soon for sequencing. If BPS facility is GLP perhaps the sequencing could be performed at BPS.
- BPS is happy with PHAC performing the sequencing and recommended that we sequence each region 4-5 times to make sure that there are not any mistakes in the sequencing.

Meeting with IDT.

- I have spoken with [redacted] and I am trying to arrange a meeting with all three parties so we can discuss the final formulation etc.
- BPS is happy about the meeting and hopefully it will occur within the next 2 weeks.
-

CRTI05-0078RTD Semi-Annual Project Review Committee Meeting
June 22, 2011
1015 Arlington Street, Winnipeg, Manitoba

Attendees:
Dr. Frank Plummer (Project Champion)
Dr. Judie Alimonti (Project Manager)
Dr. Gary Kobinger (Deputy Project Manager)
Dr. Dorothea Blandford – PHAC
Norm Yanofsky (Portfolio Manager)
Cathi Johannson (Recording Secretary)
Al Frohwerk – PHAC

Via Teleconference:
[redacted]???

Regrets:
Daniele Cole

	Agenda Item / Discussion / Action
1.	<u>Project Update</u><br><br>Dr. Plummer welcomed all attendees.<br><br>Dr. Alimonti provided a project briefing based on the attached presentation deck. It was recommended to the PRC that Jay Ramsay be brought into the project as an expert advisor – all parties agreed with the recommendation.<br><br>[redacted] provided a briefing on the progress of vaccine manufacture at IDT.
2.	<u>Round table discussions</u><br><br>[redacted]<br><br>➢ Manufacturer's questions need to be addressed.<br>➢ BPS to proceed with a post-exposure application vs. preventative.<br>➢ BPS will be re-evaluating suggestions made by the regulators.<br><br>[redacted]<br><br>➢ IDT is proceeding at a rate in which they may be ready to proceed prior to FDA approval being received for specifications for favourable first review.<br>➢ [redacted] is to initiate discussions with FDA (to be sent first week of July).<br>➢ It appears very promising that IDT will be able to generate substantial stocks of material.<br><br>Dr. Plummer:<br><br>➢ When will it be appropriate to contact Health Canada?<br>➢ When do we want to approve for use in Canada – we should look at existing

regulations. Judie will start looking into the issue. There should be lots of harmonization between Canada/US and Europe. [redacted] advised that the FDA bases specifications on current knowledge; however, additional tests and/or investigations may be required. Discussions should be initiated immediately. If FDA advice is required before IDT proceeds, decisions need to be made immediately.

[redacted]

- [redacted]

Norm Yanofsky:

- Recommended another PRC meeting be held in November or perhaps a project team meeting which would provide updates on forces, FDA status, etc.

[redacted]

- Would like to meet in person. The timing of the meeting will depend on the specifications. This meeting would provide an opportunity to solve lots of outstanding issues.

[redacted]

- Can't foresee anything in the current specifications which wouldn't be approved by the FDA.
- Is prepared to travel if a face-to-face meeting is to be held (BPS/IDT/Health Canada/PHAC).

[redacted]

- Is available if assistance is needed.

Dr. Kobinger:

- Recommends that 10^8 post-exposure testing is done prior to the next meeting.

Meeting adjourned

CRTI05-0078RTD Semi-Annual Project Review Committee Meeting
June 22, 2011
1015 Arlington Street, Winnipeg, Manitoba

PRC COMMITTEE RECOMMENDATION:

Proceed with the project in accordance with the Project Charter.

Prepared by: Cathi Johannson

RECOMMENDED FOR APPROVAL:

Judie Alimonti ____________________ Date: Sept. 16/11

Dr. Judie Alimonti – Project Manager
Biologist, PHAC-NML

APPROVED:

____________________ Date: ____________________

Dr. Frank Plummer – Project Champion
Scientific Director General, PHAC-NML

____________________ Date: ____________________

Norm Yanofsky
Portfolio Manager, DRDC-CSS

CRTI-05-0078 RD update for Frank Plummer Oct 26, 2011
Meeting with Judie Alimonti Previous update: June 8, 2011

Vaccine Manufacturing at IDT

- IDT has generated the Master Seed Virus stock (MSV)
  - They have made the MSV stock and bottled it, and are performing the various tests that have to be done for the sterility, identity, titre, adventitious agent testing, etc.
  - Majority of tests are complete. However, still have to perform the adventitious agent (AA) testing and sequencing. Hold up is on our end. They need a lot of MAb for the neutralization of the virus in the AA test and it is taking us a long time to make the quantities they will need. Also, we have been too busy to start on the sequencing of the virus.
  - The cGMP clinical batch can not be produced until all of the tests are complete on the MSV. As the AA testing and sequencing have not been completed this has delayed the cGMP production until March or April, or perhaps later. The original release date was March 2012. This means the cGMP release will be in Oct 2012.
  - IDT will start producing the 10L test batch this November or December.
- The final formulation has to be determined.
  - Requires one or more purification steps, which have yet to be determined.
  - The vaccine will be a frozen liquid and not lyophilized. The vaccine requires some downstream processing including the treatment with benzonase etc.

Research at PHAC

- Monkey experiment began in April
  - Purpose was to validate the NHP assays
  - However, it now includes a long term protection study Still working on this part
- Additional experiments planned for the year
  - When we get the 10L test batch it will be tested in mice and guinea pigs.
  - Produce the MAb and carry out all of the sequencing of the MSV stock

End use of the vaccine

A) BioProtection Systems (BPS) – human vaccine license

- BPS has licensed the VSVΔG/ZEBOV GP for human use.
- They will use a portion of 10L test batch and cGMP stock for use in a toxicology, and clinical trial.
- As both studies will be performed in the USA we do not have to go through HC at this time. BPS will use their FDA expert to contact the FDA for a pre-IND meeting. However, I have contacted HC and they said that after the FDA has approved their application then HC can also take a look at it to see if they would like anything else
- However, they are basing the IND on a post-exposure therapeutic protocol and not the vaccine protocol. They believe they will have a better chance of getting the vaccine approved using the post-exposure protocol.

Miscellaneous Material.

- EG4 position is finished at the end of December
- Jones's lab backlog
  - His last student will hopefully be graduating this year.
  - I have been writing papers on old research….so far have submitted 3, with 2 being accepted for publication.

PROJECT UPDATES
C

Topics for the meeting with [redacted] and [redacted] BPS on Tuesday May 18, 2011

Final formulation

- I informed BPS
  - IDT has said that the residual Vero DNA in the clarified prep is too high therefore there will be a purification step
  - IDT will be performing some purification trials including depth filtration and Benzonase treatment with consecutive diafiltration approaches
  - IDT also recommends to formulate the final product in a stabilizer based on amino acid/ carbohydrate formulation. It is rather risky to store rhabdoviruses without a stabilizer which prevents formation of crystals during freezing. The recommend stabilizer could facilitate freeze-drying at a later stage. Another approach could be the use of 50% buffer glycerol solution. But I would not recommend that.
- I told them that I was in the process of arranging a meeting with IDT, BPS and me so that we can discuss the formulation and any other manufacturing questions that they have. I would like to have a meeting within the next two weeks.

Adventitious agent testing

- this includes: adventitious agents testing of MSV(Adventitious agents testing is restricted to the following tests: in vivo (suckling and adult mice, guinea pigs), in vitro, retroviruses and other transforming viral agents (qPERT), bacteria and fungi, mycoplasma, mycobacteria, and other specific viral agents (if applicable-bovine, porcine, human and simian).)
- I told them that IDT recommends you get a statement from the FDA on the necessary adventitious agent testing, especially regarding the non-GLP step in the manufacture process.
- BPS said they will gently bring this up to the FDA

Assay development

- We talked about assay development and what would be the most appropriate assay to put forward as the correlates of immune protection. The difficulty arises in determining what the mechanism of protection is for the post-exposure and then proving that this protective mechanism is what is induced in humans. We are going to think about what is the best approach to put forward to the FDA. We are thinking that IFN is very important….so we might do a cytokine screen. However, there was also the confirmation that IFN is only responsible for 33% of the survival. Also, there is the fact that there is some specificity in the response as VSV MARB does not provide the same level of protection as VSV-ZEBOV.
- Should develop the assays that would be beneficial for providing the correlates of immune protection for the post-exposure treatment. It would be beneficial to determine what the best assays are. These assays are different than what we would use for the vaccine.
- Currently have the following assays
  - IL2 and IFNγ ELISPOT
  - Flow NAb assay

- VLP ZEBOV GP specific ELISA
- Flow cytometery ICS, proliferation, and immunophenotyping

Sequencing.

- We will be getting the MSV stock from IDT soon for sequencing. If BPS facility is GLP perhaps the sequencing could be performed at BPS.
- BPS is happy with PHAC performing the sequencing and recommended that we sequence each region 4-5 times to make sure that there are not any mistakes in the sequencing.

Meeting with IDT.

- I have spoken with [redacted] and I am trying to arrange a meeting with all three parties so we can discuss the final formulation etc.
- BPS is happy about the meeting and hopefully it will occur within the next 2 weeks.
-

CRTI-05-0078 RD update for Frank Plummer June 8, 2011
Meeting with Judie Alimonti Previous update: March 22, 2011

Vaccine Manufacturing at IDT

- IDT has generated the Master Seed Virus stock
  - They have made the stock and bottled it.
  - They are starting the various tests that have to be done for the sterility, identity, titre, adventitious agent testing, etc. Expected to take 2-3 months.
- have yet to negotiate on the adventitious agents and cost (amendment #3)
  - I had talked with [redacted] at [redacted] and he thought that the cost most likely will be included in the original price. But, still have to finalize it, preferably after talking to the FDA.
- The final formulation has to be determined.
  - Requires one or more purification steps, which have yet to be determined.

Research at PHAC

- Monkey experiment began in April
  - Purpose was to validate the NHP assays
  - However, it now includes a long term protection study
  - The long term protection is an extra cost but it can be covered by extra salary money in this year (~$20,000 in Bi-4 salary) and in the next year ($65,844 in EG salary and possibly another ~$20,000 in Bi-4 salary)
- Additional experiments planned for the year
  - Confirming the cGMP vaccine is as effective as the research grade vaccine. This will be confirmed in mice and guinea pigs using both the vaccine, and the post-exposure protocols.
  -

End use of the vaccine

A) BioProtection Systems (BPS) – human vaccine license

- BPS has licensed the VSVΔG/ZEBOV GP for human use.
- They will be taking a portion of the final manufactured stock for use in a toxicology, and clinical trial.
- As both studies will be performed in the USA we do not have to go through HC at this time. BPS will use their FDA expert to contact the FDA for a pre-IND meeting.
- However, they are basing the IND on a post-exposure therapeutic protocol and not the vaccine protocol. They believe they will have a better chance of getting the vaccine approved using the post-exposure protocol.

B) IDT- animal vaccine

- IDT is interested in using the vaccine to test their new animal formulation. Just starting discussions with IDT this month.

Miscellaneous Material.

- Hired EG4
  - began in April, and is working on the CRTI project and helping to finish up other projects/papers from Jones lab.
- Jones's lab backlog
  - Along with the CRTI project I am trying to finish all of the work that was left behind by Steven Jones. One student will be graduating this year.
  - Currently have 13 papers that need to be written.

CRTI05-0078RTD Semi-Annual Project Review Committee Meeting
June 22, 2011
1015 Arlington Street, Winnipeg, Manitoba

Attendees:
Dr. Frank Plummer (Project Champion)
Dr. Judie Alimonti (Project Manager)
Dr. Gary Kobinger (Deputy Project Manager)
Dr. Dorothea Blandford – PHAC
Norm Yanofsky (Portfolio Manager)
Cathi Johannson (Recording Secretary)
Al Frohwerk – PHAC

Via Teleconference:
[redacted] ????

Regrets:
Daniele Cole

	Agenda Item / Discussion / Action
1.	<u>Project Update</u><br><br>Dr. Plummer welcomed all attendees.<br><br>Dr. Alimonti provided a project briefing based on the attached presentation deck. It was recommended to the PRC that Jay Ramsay be brought into the project as an expert advisor – all parties agreed with the recommendation.<br><br>[redacted] provided a briefing on the progress of vaccine manufacture at IDT.
2.	<u>Round table discussions</u><br><br>[redacted]<br><br>➢ Manufacturer's questions need to be addressed.<br>➢ BPS to proceed with a post-exposure application vs. preventative.<br>➢ BPS will be re-evaluating suggestions made by the regulators.<br><br>[redacted]<br><br>➢ IDT is proceeding at a rate in which they may be ready to proceed prior to FDA approval being received for specifications for favourable first review.<br>➢ [redacted] is to initiate discussions with FDA (to be sent first week of July).<br>➢ It appears very promising that IDT will be able to generate substantial stocks of material.<br><br>Dr. Plummer:<br><br>➢ When will it be appropriate to contact Health Canada?<br>➢ When do we want to approve for use in Canada – we should look at existing

	regulations. Judie will start looking into the issue. There should be lots of harmonization between Canada/US and Europe. [redacted] advised that the FDA bases specifications on current knowledge; however, additional tests and/or investigations may be required. Discussions should be initiated immediately. If FDA advice is required before IDT proceeds, decisions need to be made immediately.<br><br>[redacted]<br><br>➢ [redacted]<br><br>Norm Yanofsky:<br><br>➢ Recommended another PRC meeting be held in November or perhaps a project team meeting which would provide updates on forces, FDA status, etc.<br><br>[redacted]<br><br>➢ Would like to meet in person. The timing of the meeting will depend on the specifications. This meeting would provide an opportunity to solve lots of outstanding issues.<br><br>[redacted]<br><br>➢ Can't foresee anything in the current specifications which wouldn't be approved by the FDA.<br>➢ Is prepared to travel if a face-to-face meeting is to be held (BPS/IDT/Health Canada/PHAC).<br><br>[redacted]<br><br>➢ Is available if assistance is needed.<br><br>Dr. Kobinger:<br><br>➢ Recommends that 10^8 post-exposure testing is done prior to the next meeting.
	Meeting adjourned

CRTI-05-0078 RD update for Frank Plummer Jan 13, 2012
Meeting with Judie Alimonti Previous update: Oct 26, 2011

Vaccine Manufacturing at IDT
- IDT has generated the Master Seed Virus stock (MSV)
  - They are performing the various tests that have to be done for the sterility, identity, titre, adventitious agent testing, etc. Majority of tests are complete, except for the adventitious agent (AA) assay. Hold up is on our end. IDT needs a lot of monoclonal antibodies (MAb) for the neutralization of the virus in the AA test. They need more MAb than is feasible to make so we have changed the protocol. We will bind different MAb to a resin that can be used repeatedly to remove the rVSV-ZEBOV from the vaccine. We have contracted out the MAb production due to the large quantity needed. We hope delivery of the MAb will be at the end of March. Then we will develop the protocol to remove rVSV from the vaccine. The reagents and protocols will be sent to IDT for their AA assays.
  - Despite the AA testing not being completed, it was decided that the risk of an adventitious agent being present was low therefore IDT has begun producing the 10L test batch, and is scheduling the cGMP clinical batch for March or April. All of the AA testing will be done during the spring and summer. This means the cGMP release will be in Oct 2012.
- The final formulation has to be determined.
  - The vaccine will be a frozen liquid, not lyophilized. The vaccine downstream processing includes filtration, treatment with trypsin and benzonase. The final buffer will be 10 mM Tris pH 7.2 plus a stabilizer

Research at PHAC
- Monkey experiment began in April
  - Purpose was to validate the NHP assays. This part has been done
  - However, it now includes a long term protection study Still working on this part
- Additional experiments planned for the year
  - When we get the 10L test batch it will be tested in mice and guinea pigs.
  - Produce the MAb and develop the rVSV-ZEBOV depletion protocol.

End use of the vaccine

A) BioProtection Systems (BPS) – human vaccine license
- BPS has licensed the VSVΔG/ZEBOV GP for human use.
- BPS gets a portion of 10L test batch and cGMP stock for toxicology, and clinical trials.
- As both studies will be performed in the USA we do not have to go through HC at this time. BPS will use their FDA expert to contact the FDA for a pre-IND meeting. However, I have contacted HC and they said that after the FDA has approved their application then HC can also take a look at it to see if they would like anything else
- BPS is basing the IND on a post-exposure therapeutic protocol and not the vaccine protocol. They believe they will have a better chance of getting the vaccine approved using the post-exposure protocol.

Miscellaneous Material.
- Jones's lab backlog
  - His last student will be defending her thesis in February.
  - I have been writing papers on old research….so far have submitted 3, with 2 being accepted for publication.

CRTI-05-0078 RD update for Frank Plummer Aug 2, 2012
Meeting with Judie Alimonti Previous update: Jan 13, 2011

Vaccine Manufacturing at IDT

- Generated the Master Seed Virus stock, Master Cell Bank (MCB), & 10L test batch.
  - The 10L test batch has been released. We have 8-150 ml bottles stored at the NML in a secure -80 freezer.
  - The cGMP clinical trial batch has failed. Manufacture is rescheduled for Sept.
  - Adventitious agent (AA) assay. Must remove rVSV-ZEBOV from vaccine in order to determine if there is any other agent in the vaccine that may cause harm. We had 2 grams of each anti EBOV-GP antibody made by NRC. We have bound the antibodies to a resin in order to remove the rVSV-ZEBOV from the vaccine for the AA assay. We cannot remove 100% of the rVSV-ZEBOV from the vaccine therefore we will have to go to the PCR AA test which will cost between $107,000.00-128,000.00. Awaiting a firm quote from IDT. The resin will work for the *in vivo* test as we only have to get the titre below 1 x 10^4 pfu/ml in order to obtain no pathogenesis from rVSV-ZEBOV.
- The final formulation- may change due to the manufacturing failure. Currently it is…
  - Frozen liquid in10 mM Tris pH 7.2, 2.5 mg/ml recombinant human albumin. The downstream processing includes filtration, treatment with trypsin and benzonase.

Research at PHAC

- Monkey long term protection experiment saw 25% survival at 7 and 11 months.
- Efficacy test of 10L test batch in mice (vaccine and post-exposure protocols).
  - 10L batch is slightly less potent than the R&D vaccine. Therefore will have to use higher doses to match efficacy.
- Working on the protocol for removal of the VSV-ZEBOV from the vaccine in order to test for *in vivo* adventitious agents.
  - We have about 1 month left to work out the details and then transfer the protocols to IDT to test in their system.

End use of the vaccine

A) BioProtection Systems (BPS) – human vaccine license

- BPS gets some of the cGMP stock for US based toxicology, and clinical trials.
- The pre-IND meeting with the FDA for the post-exposure protocol will be in September. They expect to start the toxicology studies in the fall, and the phase I clinical trial in February 2013.

B) Storage of the VSVΔG/ZEBOV GP for humanitarian use.

- Two possibilities for storage
  - **i.** National Emergency Stockpile Facility in Ottawa.
    - This facility is cGMP certified, secure, and heavily monitored
    - Initial funds to buy & map freezers, and put in electrical ~ $50K
  - ii. NML Biorepository
    - Not cGMP certified, but will be secure, heavily monitored etc.
    - Not yet ready but very little cost involved (to the CRTI project)

Miscellaneous Material on Jone's lab backlog.

- His last student has successfully defended her thesis.
- I continue to write papers on old research as time permits.

CRTI05-0078RTD Semi-Annual Project Review Committee Meeting
November 21, 2012
1015 Arlington Street, Winnipeg, Manitoba

Attendees:
Dr. Mike Drebot – Deputy Project Champion
Dr. Judie Alimonti – Project Manager
Dr. Diana Wilkinson - CSS
Dr. Gary Kobinger – Deputy Project Manager
Dr. Dorothea Blandford - PHAC
Cathi Johannson – Recording Secretary

Via Teleconference:
[redacted]

Regrets:
Dr. Frank Plummer – Project Champion
Norm Yanofsky – CSS Portfolio Manager
[redacted]
Christie Pronych – PHAC-NML
Florence Lopuck – PHAC-NML

	Agenda Item / Discussion / Action
1.	<u>Project Update</u><br><br>Dr. Alimonti welcomed all attendees.<br><br>Dr. Alimonti provided a project briefing based on the attached presentation deck.<br><br>[redacted] provided a project update from IDT's perspective based on the attached presentation.<br><br>Regarding the clinical batch made in the spring…there was a high endotoxin level which was identified by IDT as being from the trypsin they used. Dr. Wilkinson – Is there anything in the manufacturing process that raises the Endotoxin levels? [redacted]- yes if not correctly processed.<br><br>[redacted] advised that a problem was identified with the final vial material (too purified to confirm the identity); therefore, the method is to be changed and the Western blot will be repeated.<br><br>Dr. Drebot – What is the benefit of increased osmolality of the final clinical batch? [redacted] advised that if being injected with a liquid of low osmolality, the cells around the injection site will feel more pain.<br><br>It is critical that the batch release be received at NML by the end of March 2013 as there are no provisions for funding after that time.<br><br>PCR testing of the Adventitious Agents ('AA') for batch release is for the Master Seed Virus (MSV) and not the clinical trial material. The parameters of the PCR AA on the

	MSV has been determine. It is not known if additional testing is still required; pending discussions with the Food & Drug Administration ('FDA').<br><br>Dr. Wilkinson – Will the FDA require this go through the "animal rule" for clinical trials? [redacted]- Yes as AA testing is performed on mice and guinea pigs. [redacted] advised that it is a licensing requirement to process in accordance with this rule.<br><br>Dr. Wilkinson – Will this have to go through Health Canada if this process is followed? Dr. Alimonti – discussions have taken place with Health Canada and we will abide by the U.S.A.'s regulations. In general Health Canada and the USA FDA harmonize their standards.<br><br>Dr. Wilkinson – Will the additional work be completed in time for this project? Dr. Alimonti – Yes; however, at the cGMP level only.<br><br>Dr. Wilkinson – What is the shelf life of the test batches? Dr. Alimonti advised that the batches are to be re-titred every year, must be stored at -80 in glass bottles (can not be placed in nitro).<br><br>Dr. Alimonti advised that the 10L test batch is larger and was used to optimize the cGMP production. The cGMP is a more expensive stock batch which will be used for clinical trials and toxicology studies.<br><br>Within future financial reports, BPS's in-kind contribution should be reflected as well to recognize their contribution to the project.<br><br>Dr. Wilkinson advised that the Centre for Security Science has a thrust on commercialization opportunities. Dr. Alimonti advised that Tekmura and another US firm had all funding ceased by the National Institute of Health ('NIH') due to budget cuts in the US, and because the vaccine is considered an orphan drug. The applications will be: military usage, ring vaccination or laboratory accidents.<br><br>Dr. Kobinger advised that pigs may be amplifying hosts. IDT attempted to vaccinate non-human primates in Africa; however, the vaccine delivery system failed. IDT has requested permission to PHAC's Ebola virus vaccine a test vaccine for IDT's new vaccine delivery system. Dr. Blandford advised that if this does occur, an agreement must be in place to limit Canada's liability.
2.	<u>Round table discussions</u><br><br>Dr. Wilkinson - how many doses will be available as a result of the 10L test batch? Dr. Alimonti advised that approximately 10k doses.<br><br>Dr. Alimonti will be holding a telecom with IDT and BPS next week to ensure project on track.

CRTI05-0078RTD Semi-Annual Project Review Committee Meeting
November 21, 2012
1015 Arlington Street, Winnipeg, Manitoba

	Meeting adjourned at 10:38 a.m.

PRC COMMITTEE RECOMMENDATION:

Proceed with the project in accordance with the Project Charter.

Prepared by: Cathi Johannson

RECOMMENDED FOR APPROVAL:

______________________________ Date:______________________
Dr. Judie Alimonti – Project Manager
Biologist, PHAC-NML

APPROVED:

______________________________ Date:______________________
Dr. Mike Drebot – Deputy Project Champion
Director, Science Technology & Core Services
PHAC-NML

______________________________ Date:______________________
Norm Yanofsky
Portfolio Manager, DRDC-CSS

Re: AW: SOp numbers purity/ID
Judie Alimonti to [redacted] 2012-11-07 03:06 PM

Hi [redacted]
I have confirmed with [redacted] (from his contact with the FDA) the following PCR AA testing that needs to be performed on the MSV.

AA **testing to keep**

1x: Detection of 9CFR Bovine Viruses

1x: Quantification Of Reverse Transcriptase Activity By Ultracentrifugation And Quantitative Fluorescent Product Enhanced Reverse Transcriptase (QFPERT) Assay

1x: Real time-PCR for the Detection of Human Viruses (FDA PTC and CPMP) + Hepatitis A and B19

1x: Real time-PCR Detection of Porcine Parvovirus

All of the rest of the PCR tests are not needed.

If you would like to provide a second quote for the cGMP batch then it would just be the following 2 assays.

1x: Quantification Of Reverse Transcriptase Activity By Ultracentrifugation And Quantitative Fluorescent Product Enhanced Reverse Transcriptase (QFPERT) Assay

1x: Real time-PCR for the Detection of Human Viruses (FDA PTC and CPMP) + Hepatitis A and B19

I already have the import permits for the vaccine.

Thanks for your help. I am glad to finally get this organized.

Judie

[redacted] [redacted] [redacted]

[redacted]

[redacted]

[redacted]

PROJECT UPDATES
D

CRTI05-0078RTD Semi-Annual Project Review Committee Meeting
June 22, 2011
1015 Arlington Street, Winnipeg, Manitoba

Attendees:
Dr. Frank Plummer (Project Champion)
Dr. Judie Alimonti (Project Manager)
Dr. Gary Kobinger (Deputy Project Manager)
Dr. Dorothea Blandford – PHAC
Norm Yanofsky (Portfolio Manager)
Cathi Johannson (Recording Secretary)
Al Frohwerk – PHAC

Via Teleconference:
[redacted]???

Regrets:
Daniele Cole

	Agenda Item / Discussion / Action
1.	Project Update<br><br>Dr. Plummer welcomed all attendees.<br><br>Dr. Alimonti provided a project briefing based on the attached presentation deck. It was recommended to the PRC that Jay Ramsay be brought into the project as an expert advisor – all parties agreed with the recommendation.<br><br>[redacted] provided a briefing on the progress of vaccine manufacture at IDT.
2.	Round table discussions<br><br>[redacted]<br><br>➢ Manufacturer's questions need to be addressed.<br>➢ BPS to proceed with a post-exposure application vs. preventative.<br>➢ BPS will be re-evaluating suggestions made by the regulators.<br><br>[redacted]<br><br>➢ IDT is proceeding at a rate in which they may be ready to proceed prior to FDA approval being received for specifications for favourable first review.<br>➢ [redacted] is to initiate discussions with FDA (to be sent first week of July).<br>➢ It appears very promising that IDT will be able to generate substantial stocks of material.<br><br>Dr. Plummer:<br><br>➢ When will it be appropriate to contact Health Canada?<br>➢ When do we want to approve for use in Canada – we should look at existing

regulations. Judie will start looking into the issue. There should be lots of harmonization between Canada/US and Europe. [redacted] advised that the FDA bases specifications on current knowledge; however, additional tests and/or investigations may be required. Discussions should be initiated immediately. If FDA advice is required before IDT proceeds, decisions need to be made immediately.

[redacted]

- [redacted]

Norm Yanofsky:

- Recommended another PRC meeting be held in November or perhaps a project team meeting which would provide updates on forces, FDA status, etc.

[redacted]

- Would like to meet in person. The timing of the meeting will depend on the specifications. This meeting would provide an opportunity to solve lots of outstanding issues.

[redacted]

- Can't foresee anything in the current specifications which wouldn't be approved by the FDA.
- Is prepared to travel if a face-to-face meeting is to be held (BPS/IDT/Health Canada/PHAC).

[redacted]

- Is available if assistance is needed.

Dr. Kobinger:

- Recommends that 10^8 post-exposure testing is done prior to the next meeting.

Meeting adjourned

KEY EMAILS A

Re: AW: SOp numbers purity/ID [redacted]
Judie Alimonti to [redacted] 2012-11-07 03:06 PM

Hi [redacted]
I have confirmed with [redacted] (from his contact with the FDA) the following PCR AA testing that needs to be performed on the MSV.

AA testing to keep

1x: Detection of 9CFR Bovine Viruses

1x: Quantification Of Reverse Transcriptase Activity By Ultracentrifugation And Quantitative Fluorescent Product Enhanced Reverse Transcriptase (QFPERT) Assay

1x: Real time-PCR for the Detection of Human Viruses (FDA PTC and CPMP) + Hepatitis A and B19

1x: Real time-PCR Detection of Porcine Parvovirus

All of the rest of the PCR tests are not needed.

If you would like to provide a second quote for the cGMP batch then it would just be the following 2 assays.

1x: Quantification Of Reverse Transcriptase Activity By Ultracentrifugation And Quantitative Fluorescent Product Enhanced Reverse Transcriptase (QFPERT) Assay

1x: Real time-PCR for the Detection of Human Viruses (FDA PTC and CPMP) + Hepatitis A and B19

I already have the import permits for the vaccine.

Thanks for your help. I am glad to finally get this organized.

Judie

[redacted]

[redacted]

[redacted]

Re: Production schedule
Judie Alimonti to [redacted] 2012-11-07 02:58 PM
Cc: [redacted]

H[redacted]

I have been thinking over your proposal to delay the downstream production and filling. I am a little concerned on a few aspects of the production. I know that you have shown the virus to be stable at 4C. But at which state of production did you demonstrate this, in the bulk cell harvest, after downstream processing? I don't think the stability will be the same for both. From my experience with many types of viruses I find that 4C is only good for a short time. I was nervous when you went to 2 weeks between production and harvest. Additionally, if we were to freeze and thaw the stock then we would most likely lose 1/2 to 1 log of the titre and possible also introduce more defective interfering particles. I am not sure how much virus you made this time. Will it be enough to accommodate a loss in titre of this magnitude? As such since the virus is already in production I think that we have to make the downstream processing and bottling a priority. You have already spent a lot of money and time optimising the production protocol and now we are introducing unknowns into the process that could have detrimental impacts on the final product. If problems arise with this production, we will not have another chance to repeat it. The grant funding this project runs out in March and you would not be paid for it and we will be without a vaccine.

So overall, I would like to see a priority put on this production and see if we can speed it up and get it bottled sooner. I would like to see you put pressure on your suppliers to get the material you need. I don't think that a bottling of January is a very good idea. I have spoken with others to make sure that I am not worrying too much but they have also had the same concerns. I think we will need to discuss this further.

Thanks

Judie
[redacted]

[redacted]

[redacted]

[redacted]

[redacted]

Fw: AW: teleconference
Judie Alimonti to [redacted] 2012-11-27 09:39 AM

Hi [redacted]

I am writing to you about the current cGMP manufacturing of the VSV-ZEBOV. As you know the current production has been stretched out 2 months longer than it should be. I am really concerned that we will not have an adequate product at the end of the production. This is also of great concern to me since the timeline is so tight now that we don't have time to repeat it. I believe that there is a real possibility that we will not have a vaccine by the end of March. I want to know what can be done to set this right. The bottling date should never have been changed. You should have pushed to have your suppliers get the material you needed for the production...or you should have had everything you needed before you started.

I haven't heard from you on this subject. I think we have to meet to make sure that this is handled properly, or see if we should stop this production and start over so it can be done properly. I am really frustrated that there is no concern from IDT on the problems with this production. I was always nervous that the product was held at 4C for two weeks in the last production. I was hoping that we wouldn't have a drop in titre or a decrease in the quality of the product because of it. Then there was a drop in titre. So as you can see, holding it at 4C for 2 months is a problem. As virologists we would never do that.

Now that this project is in peril I think that we need to have a backup plan. What happens if this production fails? What can be done to rectify the situation? I would like to hear from you.

Judie Alimonti, Ph.D.
Special Pathogens Program
Public Health Agency of Canada
1015 Arlington St
Winnipeg, Manitoba, Canada
R3E 3R2
Office Ph: 204-784-5998
Lab Ph: 204-789-5097
Fax: 204-789-2140
----- Forwarded by Judie Alimonti/HC-SC/GC/CA on 2012-11-27 09:26 AM -----

[redacted]

[redacted]

[redacted]

[redacted]

[redacted]

[redacted]

Gesendet: Montag, 26. November 2012 19:47
An: [redacted]
Betreff: teleconference

H[redacted]

I have emailed [redacted] and [redacted] about having a teleconference this week. As [redacted] is away until Dec 3 her email says I should contact you. I have concerns about the current VSVZEBOV production run and I want to discuss it sooner rather than later. Below is the email that I sent to them.

I haven't heard from you about when a good date and time would be for a teleconference regarding the current VSVZEBOV production. I still have concerns regarding the delay in downstream processing and bottling. I still don't agree with the January bottling date as it is keeping the product at 4C for too long. Additionally, it is cutting the QC short as well. I think this could have a potential negative impact on the final product and I would like to discuss it with you. Because we are running short on time until the deliverable date I also wanted to discuss any possible backup plans in case this production run fails. I would like [redacted] to participate in the discussion as it will impact the timeline of his future studies.

I am available on November 28 and 29 beginning from 8 am and later Winnipeg time. Let me know what works for you.

Judie Alimonti, Ph.D.
Special Pathogens Program
Public Health Agency of Canada
1015 Arlington St
Winnipeg, Manitoba, Canada
R3E 3R2
Office Ph: 204-784-5998
Lab Ph: 204-789-5097
Fax: 204-789-2140

Re: Production VSV-ZEBOV-GP in May

Judie Alimonti to [redacted] 2013-04-15 02:28 PM

H[redacted]
Thank you for the information.

It looks as though the difference between the two buffers are just within the statistical range of each other. I would say it is marginally better without the NaCl so I am okay with going with the original formation 10 mM Tris pH 7.2, 2.5 g/L rHA, (without the NaCl).

As far as the concentration of the product during the downstream processing I have wondered if the concentration step was the reason for the large loss of virus. It seems that you don't get the corresponding increase in titre at this step. Perhaps it shouldn't be concentrated as much. I remember in the 10L test batch the concentration step was only 4X rather than the 8-20 x. (I am going from memory so this may not be exactly correct). The 10L test worked really well. I would like to discuss it further and find out at what step are you losing the product. Did you conduct the buffer tests with more concentrated virus?

As for discarding the first CTM (1.6.12). No I had not heard that your QP had barred it. But I can understand if they want to discard it. So go ahead. From our perspective the titre is too low and the endotoxin and genomic levels too high so it wouldn't be suitable for us.

Can we arrange to have a teleconference soon. I would like to discuss the next batch and what can be done. I really want this next batch to work perfectly as we are ready to move forward with the clinical trials. I would like [redacted] to be involved as well since it impacts his clinical studies . If Andreas Neubert could participate I would be grateful as he has a lot of experience and insight, and we have had a lot of problems/delays with this project. Please provide me with a date and time suitable to your group. In general we have organized the start of the teleconferences for 8 or 9 am Winnipeg time which I believe is 3 or 4 your time. It would be best if we can have this meeting before the 26th.

Judie Alimonti, Ph.D.
Special Pathogens Program
Public Health Agency of Canada
1015 Arlington St
Winnipeg, Manitoba, Canada
R3E 3R2
Office Ph: 204-784-5998
Lab Ph: 204-789-5097
Fax: 204-789-2140

[redacted]

KEY EMAILS B

Re: Production [redacted]dule
Judi[redacted]i to [redacted]
Cc: [redacted]

2012-11-07 02:58 PM

H[redacted]

I have been thinking over your proposal to delay the downstream production and filling. I am a little concerned on a few aspects of the production. I know that you have shown the virus to be stable at 4C. But at which state of production did you demonstrate this, in the bulk cell harvest, after downstream processing? I don't think the stability will be the same for both. From my experience with many types of viruses I find that 4C is only good for a short time. I was nervous when you went to 2 weeks between production and harvest. Additionally, if we were to freeze and thaw the stock then we would most likely lose 1/2 to 1 log of the titre and possible also introduce more defective interfering particles. I am not sure how much virus you made this time. Will it be enough to accommodate a loss in titre of this magnitude? As such since the virus is already in production I think that we have to make the downstream processing and bottling a priority. You have already spent a lot of money and time optimising the production protocol and now we are introducing unknowns into the process that could have detrimental impacts on the final product. If problems arise with this production, we will not have another chance to repeat it. The grant funding this project runs out in March and you would not be paid for it and we will be without a vaccine.

So overall, I would like to see a priority put on this production and see if we can speed it up and get it bottled sooner. I would like to see you put pressure on your suppliers to get the material you need. I don't think that a bottling of January is a very good idea. I have spoken with others to make sure that I am not worrying too much but they have also had the same concerns. I think we will need to discuss this further.

Thanks

Judie
[redacted]

[redacted]

Re: AW: AW: AW: WG: vaccine shipment to PHAC
Judie Alimonti to [redacted] 2013-06-19 12:58 PM

The shipment has arrived safely. Please see the attached signed receipt for your records.

PHAC sign ship doc June 19,13.pdf

Judie Alimonti, Ph.D.

Special Pathogens Program | Programme des pathogènes spéciaux
National Microbiology Laboratory | Laboratoire national de microbiologie
Public Health Agency of Canada | Agence de la santé publique du Canada
Canadian Science Centre for Human and Animal Health | Centre scientifique canadien de la santé humaine et animale
Winnipeg, Canada R3E 3P6
judie.alimonti@phac-aspc.gc.ca
Telephone | Téléphone 204-784-5998 / Facsimile | Télécopieur 204-789-2140
Government of Canada | Gouvernement du Canada

Fw: AW: teleconference
Judie Alimonti to [redacted] 2012-11-27 09:39 AM

Hi [redacted]

I am writing to you about the current cGMP manufacturing of the VSV-ZEBOV. As you know the current production has been stretched out 2 months longer than it should be. I am really concerned that we will not have an adequate product at the end of the production. This is also of great concern to me since the timeline is so tight now that we don't have time to repeat it. I believe that there is a real possibility that we will not have a vaccine by the end of March. I want to know what can be done to set this right. The bottling date should never have been changed. You should have pushed to have your suppliers get the material you needed for the production...or you should have had everything you needed before you started.

I haven't heard from you on this subject. I think we have to meet to make sure that this is handled properly, or see if we should stop this production and start over so it can be done properly. I am really frustrated that there is no concern from IDT on the problems with this production. I was always nervous that the product was held at 4C for two weeks in the last production. I was hoping that we wouldn't have a drop in titre or a decrease in the quality of the product because of it. Then there was a drop in titre. So as you can see, holding it at 4C for 2 months is a problem. As virologists we would never do that.

Now that this project is in peril I think that we need to have a backup plan. What happens if this production fails? What can be done to rectify the situation? I would like to hear from you.

Judie Alimonti, Ph.D.
Special Pathogens Program
Public Health Agency of Canada
1015 Arlington St
Winnipeg, Manitoba, Canada
R3E 3R2
Office Ph: 204-784-5998
Lab Ph: 204-789-5097
Fax: 204-789-2140
----- Forwarded by Judie Alimonti/HC-SC/GC/CA on 2012-11-27 09:26 AM -----

[redacted]

[redacted]

Gesendet: Montag, 26. November 2012 19:47
An [redacted]
Betreff: teleconference

H[redacted]
I have emailed [redacted] and [redacted] about having a teleconference this week. As [redacted] is away until Dec 3 her email says I should contact you. I have concerns about the current VSVZEBOV production run and I want to discuss it sooner rather than later. Below is the email that I sent to them.

I haven't heard from you about when a good date and time would be for a teleconference regarding the current VSVZEBOV production. I still have concerns regarding the delay in downstream processing and bottling. I still don't agree with the January bottling date as it is keeping the product at 4C for too long. Additionally, it is cutting the QC short as well. I think this could have a potential negative impact on the final product and I would like to discuss it with you. Because we are running short on time until the deliverable date I also wanted to discuss any possible backup plans in case this production run fails. I would like [redacted] to participate in the discussion as it will impact the timeline of his future studies.

I am available on November 28 and 29 beginning from 8 am and later Winnipeg time. Let me know what works for you.

Judie Alimonti, Ph.D.
Special Pathogens Program
Public Health Agency of Canada
1015 Arlington St
Winnipeg, Manitoba, Canada
R3E 3R2
Office Ph: 204-784-5998
Lab Ph: 204-789-5097
Fax: 204-789-2140

Re: Production VSV-ZEBOV-GP in May

Judie Alimonti to [redacted] 2013-04-15 02:28 PM

H[redacted]
Thank you for the information.

It looks as though the difference between the two buffers are just within the statistical range of each other. I would say it is marginally better without the NaCl so I am okay with going with the original formation 10 mM Tris pH 7.2, 2.5 g/L rHA, (without the NaCl).

As far as the concentration of the product during the downstream processing I have wondered if the concentration step was the reason for the large loss of virus. It seems that you don't get the corresponding increase in titre at this step. Perhaps it shouldn't be concentrated as much. I remember in the 10L test batch the concentration step was only 4X rather than the 8-20 x. (I am going from memory so this may not be exactly correct). The 10L test worked really well. I would like to discuss it further and find out at what step are you losing the product. Did you conduct the buffer tests with more concentrated virus?

As for discarding the first CTM (1.6.12). No I had not heard that your QP had barred it. But I can understand if they want to discard it. So go ahead. From our perspective the titre is too low and the endotoxin and genomic levels too high so it wouldn't be suitable for us.

Can we arrange to have a teleconference soon. I would like to discuss the next batch and what can be done. I really want this next batch to work perfectly as we are ready to move forward with the clinical trials. I would like [redacted] to be involved as well since it impacts his clinical studies . If Andreas Neubert could participate I would be grateful as he has a lot of experience and insight, and we have had a lot of problems/delays with this project. Please provide me with a date and time suitable to your group. In general we have organized the start of the teleconferences for 8 or 9 am Winnipeg time which I believe is 3 or 4 your time. It would be best if we can have this meeting before the 26th.

Judie Alimonti, Ph.D.
Special Pathogens Program
Public Health Agency of Canada
1015 Arlington St
Winnipeg, Manitoba, Canada
R3E 3R2
Office Ph: 204-784-5998
Lab Ph: 204-789-5097
Fax: 204-789-2140

[redacted]

KEY EMAILS C

Re: AW: AW: AW: WG: vaccine shipment to PHAC

Judie Alimonti to: [redacted] 2013-06-19 12:58 PM

The shipment has arrived safely. Please see the attached signed receipt for your records.

PHAC sign ship doc June 19,13.pdf

Judie Alimonti, Ph.D.

Special Pathogens Program | Programme des pathogènes spéciaux
National Microbiology Laboratory | Laboratoire national de microbiologie
Public Health Agency of Canada | Agence de la santé publique du Canada
Canadian Science Centre for Human and Animal Health | Centre scientifique canadien de la santé humaine et animale
Winnipeg, Canada R3E 3P6
judie.alimonti@phac-aspc.gc.ca
Telephone | Téléphone 204-784-5998 / Facsimile | Télécopieur 204-789-2140
Government of Canada | Gouvernement du Canada

KEY EMAILS D

Re: AW: AW: MAb testing

Judie Alimonti to [redacted] 2011-07-04 01:17 PM

Hi [redacted]

I said I would get back to you in regards to the questions you asked below.

You Asked the following: **with responses in red**

Delivery of Preclinical Stocks: In the main contract stated that you will need 10L. If you really need 10L when should we deliver this material, which titer you will need, which testing is required (sterility, mycoplasma, mycobacteria ... something else for your preclinical study?) and which aliqot size do you need?

When I took over this project the 10L test batch was already part of the contract, which is why I just left it in. I am not sure why they decided on 10L. Perhaps at the time they weren't sure what the titre was going to be. Even at 1 ml per bottle it would amount to 10,000 vials. So you are right, we may not require this volume. Let me work up all of the experiments that we plan to do with this and I will see what volume might work. I will find out why they picked 10L and see if there is some reason why it has to be that high.

As to the titre... I think we are going to stick with 1E+08 pfu/ml at this time. We had talked about it in the PRC meeting and I think we had decided to stick with this titre in light of the fact that we are not sure exactly which dose we will need. Also, BPS thought that they could just increase the dose volume but this might still cause a problem as the total amount of host DNA administered would still be a concern. As to the aliquot...I think 1 ml minimum would work great. For 10L we may be able to do more...2 ml perhaps. When do you need to know exactly what volume is needed? As you are the expert..please tell me what is the normal volume size that you produce?

We talked about the testing that had to be done as well. Of course all of the testing that is mentioned in the contract, what you had put in the PRC presentation. However, at the PRC meeting there was an additional question about what other testing the FDA might ask for. Unfortunately we have to wait for BPS to have that meeting with the FDA before we can answer this.

Delivery time...I have to work out storage at this end. I think I should be able to have this organised by October.

From the MSV production we left approx. 1000 mL with a titer of approx. 1E+08 pfu/mL. Would this meet your requirements in view of volume, titer and testing? This material is almost ready for shipment.

I am assuming that you will be sending only 1-2 vials of the MSV. I just have to check with the import permit. I have the Health Canada one already but I just have to check with the CFIA and see if I have received it yet. As soon as I have it I will be ready for the shipment.

If the MSV do not meet you requirements we will produce the preclinical material (10L or less) in September 2011 + time for testing (6 to xx weeks depending on the testing needs).

Judie Alimonti, Ph.D.
Special Pathogens Program
Public Health Agency of Canada

summary of vaccine manufacture .
Judie Alimonti to: [redacted] 2012-07-19 01:26 PM

Hi [redacted]
I have some of the information for you.

I have talked to [redacted] and she said IDT is going to remake the cGMP batch. This will be made in Sept with filling the vials by the end of October. This means the release may be in the new year.

She is not sure why there was such a loss in titre between running the batch and filling the bottles but she is looking into it. They are going to run some studies. They may change the buffer so it includes sucrose...we will see when the studies are complete.

As for the adventitious agent testing, it looks like the normal procedure can be done for the in vivo studies in mice. In suckling mice there was some pathology if the titre was above 1x10^4 pfu. So I am establishing a protocol to remove the virus from the vaccine so that it is below 1x10^4 pfu when it is injected into suckling mice. On the other hand I have been unable to completely remove all virus from the vaccine. No matter how much neutralizing antibody I add it seems as though there is always one or two viruses left that can develop into CPE. Therefore we have decided to go with the PCR route for the adventitious agent testing for the in vitro portion.

Here are some of the questions that [redacted] wants us to ask the FDA. Her biggest concern is the testing strategy for the AA testing. We are going to use the virus removed vaccine for the in vivo tests on the MSV and the cGMP batch. So these assays are using the normal protocol However, based on her comment below it looks like she is asking if the in vivo tests are sufficient ??? and that the in vitro tests are unnecessary. However, I know she had suggested in another email to do PCR for the in vitro tests. So perhaps these questions could be phrased appropriately to the FDA. I think she also wants to know what other agents need to be tested in addtion to the usual aventitious agents that they test for. PLease see her comments below.

In regard to testing strategy ... This is something which must be discussed with the FDA ... we need to ask the right question to get confirmation the following strategy:

1. We need confirmation of the MCB testing ... according to the COA we should have sufficient testing for Phase I/II ... EOP testing will be for later clinical phase
2. We need confirmation on the human and simian testing PCR of the MCB
3. We will test the MSV with the same human and siman PCR as the MCB. For AA we will only do the test invivo (guinea pigs, mice, suckling mice (virus reduction required) and **no** invitro assay. But will offer the strategy for virus neutralisation for later clinical phase studies
4. For the CMT we have a complete serum free process, we will only do the AA in vivo and no AA and no invitro testing with the same argue as for the MSV.

Please talk to [redacted] and [redacted] rior your Pre-IND

I am providing you the manufacturing summary as an attachment. [redacted] lso sent along some information regarding the removal of the virus from the vaccine. I think there were some questions there for the FDA as well.

I am supposed to receive the other document you requested in a day or two.

Let me know if you need anything.

Judie

Strategy of rVSV ZEBOVGP-removal before testing of adventitious agents.docx

rVSV ZEBOV process flow for Pre-IND.docx

Re: IDT contract

Ron Bouchard to: Judie Alimonti, Dorothea Blandford 2013-11-25 12:37 PM
Cc: Ron Bouchard

History: This message has been replied to.

Dear Dorothea and Judie,

Please see attached Memo. If you have any questions, please don't hesitate to ask.

Cheers.

Yours truly,

Ron

RAB IDT Contract Memo Nov 25 2013.docx

Ron A. Bouchard, PhD, JD, LLM
Senior Advisor, Intellectual Property Management | Conseiller Principal en Administration de Propriété Intellectuelle
Office of Intellectual Property Management & Business Development | Bureau de la gestion de la propriété intellectuelle et du développement commercial
Public Health Agency of Canada | Agence de la santé publique du Canada
National Microbiology Laboratory | Laboratoire national de microbiologie
1015 Arlington Street, Winnipeg, Manitoba R3E 3R2 | 1015, rue Arlington Winnipeg (Manitoba) R3E 3R2
ron.bouchard@phac-aspc.gc.ca
Telephone | Téléphone 204-789-2062
Facsimile | Télécopieur 204-789-2097
Government of Canada | Gouvernement du Canada

Judie Alimonti Hi, BPS is in the process of making a pre-... 2013-11-25 08:06:27 AM

From:	Judie Alimonti/HC-SC/GC/CA
To:	Ron Bouchard/HC-SC/GC/CA@HWC
Cc:	Dorothea Blandford/HC-SC/GC/CA@HWC, Ron Bouchard/HC-SC/GC/CA@HWC
Date:	2013-11-25 08:06 AM
Subject:	Re: IDT contract

Hi,
BPS is in the process of making a pre-IND submission to the FDA but not to HC.

I am pretty busy monday and tuesday but I am avialable on wednesday before 11 and after 2 or on thursday morning.

Judie

Ron Bouchard	Hello Judie and Dorothea, Haven't heard fro...	2013-11-24 12:26:08 PM
Ron Bouchard	Hello All, I'm working my way through the doc...	2013-11-23 02:48:34 PM
Judie Alimonti	Hi Ron, I am sending you the IDT contrac...	2013-11-14 02:46:19 PM

KEY EMAILS E

Re: WG: Report for Method Suitability Test : Sterility (of the product rVSV-ZEBOV-GP MSV)

Judie Alimonti to [redacted] 2011-09-13 03:52 PM

Hi [redacted]

Thank you for reminding me to reply as your email got lost amongst many others. I have read over the information you have sent to me and I am sending you the signature page with my signature.

Thank you

suitability signature.pdf

Judie Alimonti, Ph.D.
Special Pathogens Program
Public Health Agency of Canada
1015 Arlington St
Winnipeg, Manitoba, Canada
R3E 3R2
Office Ph: 204-784-5998
Lab Ph: 204-789-5097
Fax: 204-789-2140

[redacted]

Hi [redacted]
We will be doing the tests and not BPS. Once we have the information I will send it to you.

Judie Alimonti, Ph.D.
Special Pathogens Program
Public Health Agency of Canada
1015 Arlington St
Winnipeg, Manitoba, Canada
R3E 3R2
Office Ph: 204-784-5998
Lab Ph: 204-789-5097
Fax: 204-789-2140

[redacted]

[redacted]

[redacted]

[redacted]

[redacted]

Test	SOP/ Method4)	Mate rial	Demand	Sample volume6), 8)
Test for purity[7)]	SOP to be defined (PHAC)	Final vial (MS V)	No wild type VSV specific GP	Same sample as identity test (PCR)
Test for identity (PCR)[7)]	SOP to be defined (PHAC)	Final vial (MS V)	Presence of insert ZEBOV-GP	1 vial
Identity by sequenc ing[7)]	SOP to be defined (PHAC)	Final vial (MS V)	Sequence identical to master sequence	1 vial
